# Slc25a21 in cisplatin-induced acute kidney injury: a new target for renal tubular epithelial protection by regulating mitochondrial metabolic homeostasis

**DOI:** 10.1038/s41419-024-07231-2

**Published:** 2024-12-18

**Authors:** Xin Su, Mi Bai, Yaqiong Shang, Yang Du, Shuang Xu, Xiuli Lin, Yunzhi Xiao, Yue Zhang, Huimei Chen, Aihua Zhang

**Affiliations:** 1 Department of Nephrology, Children’s Hospital of Nanjing Medical University, Guangzhou Road 72, Nanjing, 210008 China; 2 Nanjing Key Laboratory of Pediatrics, Children’s Hospital of Nanjing Medical University, Nanjing, 210008 China; 3 Jiangsu Key Laboratory of Pediatrics, Nanjing Medical University, Nanjing, 210029 China; 4 Centre for Computational Biology and Programme in Cardiovascular and Metabolic Disorders, Duke-NUS Medical School, 8 College Road, 169857 Singapore, Singapore

**Keywords:** Pathogenesis, Acute kidney injury

## Abstract

Acute kidney injury (AKI) is a significant global health issue, which is often caused by cisplatin therapy and characterized by mitochondrial dysfunction. Restoring mitochondrial homeostasis in tubular cells could exert therapeutic effects. Here, we investigated Slc25a21, a mitochondrial carrier, as a potential target for AKI intervention. Renal Slc25a21 expression is negatively associated with kidney function in both AKI patients and cisplatin-induced murine models. Sustaining renal expression of Slc25a21 slowed down AKI progression by reducing cellular apoptosis, necroptosis, and the inflammatory response, likely through its regulation of 2-oxoadipate conversion. Slc25a21 is highly expressed in proximal tubular epithelial cells, and its down-regulation contributes to compromised mitochondrial biogenesis and integrity, as well as impaired oxidative phosphorylation. Mechanistically, reduced Slc25a21 in AKI disrupts mitochondrial 2-oxoadipate transport, affecting related metabolites influx and the tricarboxylic acid cycle. These findings demonstrate a previously unappreciated metabolic function of Slc25a21 in tubular cells, and suggest that targeting mitochondrial metabolic homeostasis by sustaining Slc25a21 expression could be a potential novel therapeutic strategy for AKI.

## Introduction

Acute kidney injury (AKI) is defined as a rapid deterioration of renal function, manifested by an increase in serum creatinine level, with or without a decline in urine output [1, 2]. It is estimated that over 13.3 million people worldwide suffer from AKI annually, which leads to approximately 1.7 million deaths [3]. Notably, survivors of AKI are known to face a heightened risk of developing chronic kidney disease (CKD), with some cases progressing to end-stage renal disease (ESRD) [4]. Cisplatin, a cytotoxic chemotherapeutic drug, is one of the most common causes of AKI [5]. About 50–70% of patients receiving cisplatin chemotherapy may develop AKI [6]. Among renal cells, proximal tubular epithelial cells are particularly susceptible to damage from cisplatin, and they typically show distinct pathological changes such as tubular dilatation, brush border loss, and cell necrosis [7]. Despite various mechanisms for cisplatin-induced AKI have been proposed [5, 7–9], the precise underlying processes remain unclear and, most critically, effective interventions to prevent or reverse AKI remain very limited.

Recent research has identified mitochondrial dysfunction as a primary factor in the development of AKI [10, 11]. The kidney is the second most metabolically active organ in human body with an abundance of mitochondria, where ATP is predominantly synthesized via mitochondrial oxidative phosphorylation (OXPHOS) [12]. Specifically, glucose and lipid metabolism are instrumental in the generation of acetyl-CoA, a crucial substrate that fuels the tricarboxylic acid (TCA) cycle, thereby facilitating ATP synthesis [2, 13]. In AKI, mitochondrial damage disrupts the normal mitochondrial OXPHOS, leading to a significant alteration in the kidney’s metabolic state [14–16]. This reprogramming of energy metabolism results in decreased cellular ATP levels and a concurrent increase in reactive oxidative species (ROS). These metabolic perturbations trigger tubular cell apoptosis and necroptosis as well as inflammatory response, which exacerbate the pathogenesis of AKI [17, 18].

Given these metabolic disruptions in AKI, several therapeutic strategies targeting renal metabolism, which focus on restoring energy balance and mitochondrial homeostasis, have been proposed in AKI. For instance, the administration of glutamine has been shown to boost the TCA cycle, thereby partially restoring energy production and attenuating the kidney damage typically observed in AKI [19, 20]. Additionally, given its capacity to counteract the effects of acetyl-CoA depletion, sodium acetate supplementation has been also considered as a promising therapeutic avenue for ameliorating kidney injury [21, 22].

Mitochondrial 2-oxodicarboxylate carrier (ODC), also called SLC25A21, is essential for the translocation of 2-oxoadipate and 2-oxoglutarate across the mitochondrial inner membrane through a counter exchange mechanism [23]. 2-oxoadipate, also known as 2-oxoadipic acid, entering the mitochondria is converted into acetyl-CoA, facilitating ATP production. Our previous study showed that overexpression of SLC25A21 in a yeast mitochondrial dysfunction model, substantially enhanced mitochondrial respiration, associated with elevated levels of complex IV and TCA-mediated ATP production [24]. In contrast, SLC25A21 deficiency contributes to the accumulation of 2-oxoadipate, leading to mitochondrial dysfunction and neurotoxicity in spinal muscular atrophy–like disease [25].

In this study, we investigated the role of SLC25A21 as a novel target for regulating metabolic processes in renal tubular cells, and mitigating kidney dysfunction in AKI. We reported the reduced expression of Slc25a21 in both AKI patients and mice induced by cisplatin, and showed that down-regulation of Slc25a21 was associated with kidney dysfunction in AKI. Using both in vivo AKI murine models and in vitro cisplatin-exposed mouse proximal tubule cells (TKPTS) lines, combined with metabolomics analysis, we further demonstrated that maintaining renal Slc25a21 expression protects renal tubules from cisplatin-induced damage and alleviates cell apoptosis and necroptosis as well as inflammation, thereby preventing the progression of AKI. These studies highlight the role for Slc25a21 in facilitating mitochondrial 2-oxoadipate transport, regulating metabolites influx and enhancing energy metabolism in AKI. Our findings might pave the way to design novel therapeutic interventions in AKI, targeting mitochondrial homeostasis.

## Methods

### Patients

Renal biopsy samples were acquired from four patients diagnosed with acute tubular necrosis at Children’s Hospital of Nanjing Medical University, China. The AKI samples include four pediatric patients, with a median age of approximately 4 years, and a male-to-female ratio of 1:3. These children presented with AKI characterized by oliguria or anuria and elevated serum creatinine, caused by drug toxicity, sepsis, or ischemia. The pediatric patient details are provided in Table S1. Normal renal tissues, also obtained from para-carcinoma kidney tissues, were collected from five children (<14 years old; the male-to-female ratio is 3:2.) who underwent renal carcinoma resection.

### Animals

8-week-old male C57BL/6 J mice were purchased from GemPharmatech (Nanjing, China) and maintained under a 12-hour light/ 12-hour dark cycle with free access to food and water in the animal facility of Nanjing Medical University (Nanjing, China). Slc25a21^AAV9^ mice were generated by kidney multipoint in situ injection of adeno-associated virus 9 (AAV9) expressing Slc25a21 gene (pAAV-CMV-Slc25a21-HA-WPRE, 4 × 10^11^ virus genome copies), while AAV9 containing scrambled sequence served as control (NC^AAV9^, pAAV-CMV-MCS-WPRE) (OBiO, Shanghai, China). The surgery procedure was performed following the instruction of previously reported study [26]. To deliver the vector, 32 G, 50 µl syringes (Conpuvon Company, China) were used for multi-point in situ injection into the renal cortex. Slc25a21^DNA^ mice were generated through high-pressure tail-vein injection of 70 μg Slc25a21 plasmids within 7 sec. The empty vector (pcDNA3.1) served as negative control (NC^DNA^). Cisplatin-induced AKI model was obtained by intraperitoneal injection of 25 mg/kg cisplatin (Sigma-Aldrich) and the same volume of saline were injected into the controls [27]. Mice were sacrificed after 3 days of injection.

### Cell culture

Mouse proximal tubule cells (TKPTS; Cat# CRL-3361; RRID: CVCL_UJ13) lines were procured from ATCC, cultured in DMEM/F-12 medium (Gibco, 319-075-CL) supplemented with 7% Fetal Bovine Serum and 0.6% insulin. The cells were maintained at 37 °C with 5% CO2 and 95% air. The TKPTS were transfected with Slc25a21 plasmids or empty vector (pcDNA3.1 served as negative control) using JetPRIME® transfection reagent (Polyplus, 101000046) following the manufacturer’s instructions and then treated with cisplatin (10 µM) for 24 h. Cells were regularly monitored for mycoplasma via qPCR under ISO 17025, ensuring mycoplasma-free conditions.

### Cell viability assay

TKPTS cells were pre-seeded in a 96-well plate and transfected with Slc25a21 plasmids or empty vector (pcDNA3.1 as a negative control) after cisplatin (10 µM) treatment for 24 h. Cell viability was assessed using Cell Counting Kit-8 (CCK-8) (ApexBio, K1018) in accordance with the manufacturer’s instructions. Following the respective treatments, 10 μl of the CCK-8 solution was added to each well of the plates (care should be taken to avoid introducing bubbles into the wells, as they can interfere with absorbance) and incubated for 2 h at 37 °C. The absorbance was measured at 450 nm using a microplate reader. Cell viability was calculated using the following formula:

Cell viability (%) = [(As - Ab) / (Ac - Ab)] x 100%, where As is the absorbance of the tested vectors (absorbance of the well containing cells, culture medium, CCK-8, and tested vectors), Ab is the absorbance of the blank (absorbance of the well containing culture medium and CCK-8), and Ac is the absorbance of the control (absorbance of the well containing cells, culture medium, and CCK-8).

### Western blotting analysis

Total proteins from renal tissue (25–30 mg) or cultured cells (10^6^) were extracted using RIPA lysis buffer (Beyotime, P0013K) containing the protease inhibitor cocktail (Roche, 04693132001). The lysate concentrations were determined using the BCA protein assay kit (Thermo-Fisher, 23227) and separated by sodium dodecyl sulfate-polyacrylamide gel electrophoresis (SDS-PAGE). Following gel electrophoresis, the proteins were transferred onto polyvinylidene fluoride (PVDF) membranes, which were then blocked with 5% non-fat milk powder for 1 h at room temperature (RT). Subsequently, the membranes were incubated with primary antibodies overnight at 4 °C. The primary antibodies used in this study included: SLC25A21 antibody (Thermo Fisher Scientific, PA5-101672; RRID: AB_2851106); NGAL antibody (Abcam, ab63929; RRID: AB_1140965); Mouse KIM-1 Antibody (R&D Systems, AF1817; RRID: AB_2116446); BAX (Cell Signaling Technology, 2772; RRID: AB_10695870); Cleaved Caspase3 (Cell Signaling Technology, 9664; RRID: AB_2070042); P53 (Cell Signaling Technology, 9282; RRID: AB_331476); β-Actin Rabbit mAb (High Dilution) (ABclonal, AC026; RRID: AB_2768234); α-Tubulin Mouse mAb (ABclonal, AC012; RRID: AB_2768341); Mouse anti HA-tag mAb (ABclonal, AE008; RRID: AB_2770404); NDUFB8 Rabbit mAb (ABclonal, A19732; RRID: AB_2938519); SDHB Rabbit mAb (ABclonal, A1809; RRID: AB_2861883); UQCRC2 Rabbit mAb (ABclonal, A4366; RRID: AB_2863250); COXIV Rabbit pAb (ABclonal, A6564; RRID: AB_2767158); ATP5A1 Rabbit mAb (ABclonal, A11217; RRID: AB_2861524); Anti-mtTFA antibody - Mitochondrial Marker (Abcam, ab47517; RRID: AB_945799); PGC1α Rabbit mAb (ABclonal, A20995; RRID: AB_3096101); ACAA2 Rabbit mAb (ABclonal, A0664; RRID: AB_3096102); DHTKD1 Rabbit pAb (ABclonal, A8369; RRID: AB_2769161); Citrate synthetase Rabbit mAb (ABclonal, A23371; RRID: AB_3096103); OGDH Rabbit mAb (ABclonal, A22163; RRID: AB_3096104); SUCLG2 Rabbit pAb (ABclonal, A19872; RRID: AB_2862784); ACO2 Rabbit mAb (ABclonal, A4524; RRID: AB_2863288); MT-ND1 Rabbit mAb (ABclonal, A9743; RRID: AB_3096106); ATP6 Rabbit pAb (ABclonal, A8193; RRID: AB_2768510); MT-CYB Rabbit pAb (ABclonal, A17966; RRID: AB_2861768); COX1 Rabbit mAb (ABclonal, A23123; RRID: AB_3096107); Membrane Integrity WB Antibody Cocktail (Abcam, ab110414; RRID: AB_2687585). PIPK1/RIP Rabbit pAb (ABclonal, A7414; RRID: AB_ 2767944), PIPK3 Rabbit pAb (ABclonal, A25050; RRID: AB_3662682), MLKL Rabbit mAb (ABclonal, A26436; RRID: AB_3662683). The PVDF membranes were then incubated with HRP-tagged secondary antibodies (HRP-conjugated Mouse Anti-Rabbit IgG (H + L), AS079; RRID: AB_2864059; HRP-conjugated Goat Anti-Mouse IgG Light Chain, AS062; RRID: AB_2864056) for 1 h at RT. The bands were visualized using an enhanced chemiluminescence kit (Tanon, 180-501) on a Tanon 5200 Chemiluminescent Imaging System (Tanon, China). Band quantification was performed using Image J software (RRID: SCR_003070) and normalized to loading controls.

### Quantitative real-time polymerase chain reaction (qRT-PCR)

Total RNA from kidney tissues (25–30 mg) and cultured cells (10^6^) was extracted using Trizol reagent (TaKaRA Biotechnology, 9109) following the manufacturer’s instructions. Subsequently, 1 μg of total RNA was reverse transcribed to cDNA using HiScript II Q RT SuperMix for qRT-PCR (Vazyme Biotechnology, R222-01) according to the manufacturer’s protocol. The resulting cDNA was then subjected to qRT-PCR amplification using SYBR Green master mix (Vazyme, q111-02/03) on a LightCycler®96 system (Roche, Germany). The temperature conditions were executed according to the manufacturer’s instructions for the SYBR Green master mix. Relative mRNA expression levels were calculated using the 2^-∆∆Ct^ method and normalized to β-Actin expression. Primer sequences used in the study were obtained from the Primer Bank and are listed in Table S2.

### Mitochondrial DNA copy number

Total DNA was extracted from Kidney tissue using DNeasy Tissue Kit (QIAGEN Sciences, 69506) following the manufacturer’s instructions. The primer designed by Primer 5 software was listed in Table S2. qRT-PCR was performed for the determination of mtDNA copy number. Relative mtDNA copy number was calculated using the 2^-∆∆Ct^ method and normalized by Rsp18.

### Oxygen Consumption Rate (OCR) measurement

The measurement of oxygen consumption rate was conducted using the Seahorse XF-96 Extracellular Flux Analyzer (Seahorse Bioscience, Copenhagen, Denmark). Briefly, TKPTS cells (10^4^) were initially were pre-seeded in a Seahorse assay 96-well plate and transfected with Slc25a21 plasmids or empty vector (pcDNA3.1 as a negative control). After 6 h, cisplatin (10 µM) was added to the cell culture medium for 24 h. On the day of the experiment, the cell medium was replaced by Seahorse assay medium, and cells were incubated at 37 °C in a CO_2_-free incubator for 1 h prior to assessing the basal OCR. Half an hour before the experiment, the sensor was placed into the XF-96 instrument, and calibration was initiated. After calibration, the calibration fluid plate was replaced with the cell plate, and measurements began. Basal oxygen consumption was recorded for 20 min, and the inhibitors were subsequently introduced as follows [1]: Oligomycin (1 µM), which blocks the proton channel of the Fo portion of ATP synthase, thereby inhibiting ATP synthesis, was employed to assess the coupling of ATP synthesis with the respiratory rate [2]. Carbonyl cyanide 4-trifluoromethoxy-phenylhydrazone (FCCP, 0.5 µM), a compound that dissipates the proton gradient across the mitochondrial membrane, was used to determine the maximal respiration rate [3]. Rotenone (ROT, 0.5 µM) and antimycin A (0.5 µM), inhibitors of Complex I and Complex III, respectively, were employed to measure the non-mitochondrial oxygen consumption rate. All these compounds were automatically injected into each well by the XF-96 instrument. The determined cellular bioenergetics parameters were the following: basal and maximal respiration.

### Reactive oxygen species (ROS) measurement

The assessment of ROS production was determined using MitoSOX^TM^ Red mitochondrial superoxide indicator for live-cell imaging (Invitrogen, M36008) according to the manufacturer’s instructions. Briefly, TKPTS cells were pre-seeded in a 12-well plate and transfected with Slc25a21 plasmids or empty vector (pcDNA3.1 as a negative control) after cisplatin (10 µM) treatment for 24 h. 5 µM MitoSOX^TM^ reagent was applied to cover the cells, followed by incubation for 10 min at 37 °C, protected from light. The cells were then washed three times with warm buffer, and ROS levels were determined by flow cytometry at 510/580 nm.

### ELISA assay of IL-18 and TNFα

The concentrations of IL-18 and TNFα in the serum of mice measured by Mouse IL-18 ELISA Kit (ABclonal, RK00104) and Mouse TNFα ELISA Kit (ExCell Bio, EM008-96) in accordance with the manufacturer’s instructions. Briefly, prepared all necessary reagents, working standards, and samples. Washed each well for 40 s, then repeated. Added 100 μl of standard/sample for each well. Utilized the provided adhesive sealer to cover the wells and incubated the plate at 37 °C for 2 h. Prepared the concentrated biotin conjugate antibody (30x) working solution. Repeated the aspiration/wash process, then added 100 μl of the working biotin conjugate antibody to each well and incubated at 37 °C for 1 h. Prepared the streptavidin-HRP concentrated (30x) working solution. Repeated the aspiration/wash process, then added 100 μl of the working streptavidin-HRP to each well and incubated at 37 °C for 1 h. Repeated the aspiration/wash process. Added 100 μl of TMB Substrate to each well, followed by a 15–20 min incubation at 37 °C in the dark. Added 50 μl of stop solution, then measured the optical density of each well within 5 min using a microplate reader at 450 and 630 nm.

### Determination of ATP content

The ATP content in kidney tissue (25–30 mg) was determined using an Enhanced ATP Assay Kit (Beyotime, S0027) according to the manufacturer’s instructions. After complete tissue lysis, the lysate was centrifuged at 4 °C, 12,000 × *g* for 5 min, and the supernatant was collected for subsequent analysis. The ATP concentration was determined by preparing an ATP standard curve and measuring the concentration of the supernatant accordingly. Additionally, the protein concentration of the supernatant was measured using the BCA protein assay kit (Thermo-Fisher, 23227), and the ATP content was calculated per one µg of protein.

### Serum creatinine (SCr) and blood urea nitrogen (BUN) analysis

Venous blood was collected from mice in an EP tube containing anticoagulant, followed by centrifugation at 5000 rpm/min for 30 min to separate plasma. The upper serum was then obtained. Serum creatinine levels were analyzed using the Beckman Creatinine Analyzer II (DXC800; Beckman Coulter). Blood urea nitrogen was measured using a BUN Colorimetric Detection Kit (EIABUN; Invitrogen, Carlsbad, CA).

### Periodic acid-Schiff (PAS) staining

Kidney tissues obtained from mice were fixed in 4% paraformaldehyde, embedded in paraffin, and transversely sectioned to a thickness of 3 μm for renal pathology assessment. The sections were then subjected to dewaxing and rehydration using the following sequence: xylene I for 10 min; xylene II for 10 min; 100% ethanol I for 3 min; 100% ethanol II for 3 min; 95% ethanol for 3 min; 80% ethanol for 3 min; 70% ethanol for 3 min; distilled water for 1 min.

Renal sections underwent oxidation with 1% periodate at 37 °C for 10 min, followed by rinsing with distilled water. Subsequently, the sections were incubated with Schiff dye at 42 °C for 40 min and rinsed with tap water for 10 min. Hematoxylin staining was carried out at 37 °C for 3 min, followed by rinsing with a solution of 1% HCl and ethanol for 2 sec. After removing HCl, the sections were returned to a blue state for 1 min and rinsed with tap water for 10 min.

Dehydration steps included 70% ethanol for 3 min, 80% ethanol for 3 min, 90% ethanol for 3 min, 100% ethanol for 3 min, and xylene I, II, III for 3 min each. Finally, the renal sections were sealed with neutral resin. Photomicrographs of the renal sections were captured using an Olympus BX51 microscope (Olympus, Center Valley, PA, USA) and analyzed by a pathologist in a blinded manner. The tubular injury score was assigned as follows: 0 for normal histology, 1 for mild injury (5-25% injury), 2 for moderate injury (25-50% injury), 3 for severe injury (50–75% injury), and 4 for critical injury (more than 75% injury). At least 10 fields were examined and scored for each slide.

### Immunohistochemical (IHC) analysis

Mouse kidney tissues were fixed in 4% paraformaldehyde, embedded in paraffin, and transversely sectioned to a thickness of 3 μm for renal pathology slides. After deparaffinization, renal sections were washed three times with PBS and subjected to antigen retrieval by boiling in Citrate Antigen Retrieval Solution (Beyotime, P0083) for 20 min, followed by cooling to RT. Subsequent steps were conducted using the Universal Two-Step Test Kit (Mouse/Rabbit Enhanced Polymer test system) (ZSGB Bio, PV-9000) following the manufacturer’s instructions. The sections were incubated overnight at 4 °C with primary antibodies against SLC25A21 (Thermo Fisher Scientific, PA5-101672; RRID: AB_2851106), Mouse anti HA-tag mAb (ABclonal, AE008; RRID: AB_2770404) and 8-OHdG (Santa Cruz, sc-66036; RRID: AB_832272). Peroxidase conjugate localization was determined using a DAB kit (ZLI-9018, zsbio). Renal sections were photographed with an Olympus BX51 microscope (Olympus, Center Valley, PA, USA), and the signals were analyzed using Image J software (RRID: SCR_003070).

### Immunofluorescence staining

Immunofluorescence staining was also performed on paraffin-embedded renal sections, following the same deparaffinization, antigen retrieval, and rehydration steps as the IHC staining process. The renal sections were incubated overnight at 4 °C with the primary antibody SLC25A21 Antibody (Thermo Fisher Scientific, PA5-101672), HA-Tag (C29F4) Rabbit mAb (Cell Signaling Technology, 3724S; RRID: AB_3662684), Aquaporin/AQP2 (E-2) (Santa Cruz, sc-515770; RRID: AB_2810957) and E-cadherin Antibody (G-10) (Santa Cruz, sc-8426; RRID: AB_626780). The next day, Alexa Fluor 488-conjugated Donkey anti-rabbit IgG (H + L) (ABclonal, AS035 RRID: AB_2768318) was used as a secondary antibody. Nuclei were counterstained with DAPI (Beyotime, P0131). Lotus tetragonolobus lectin (LTL) fluorescence (Vector Laboratories, ZG1023) was simultaneously incubated with the primary antibody. Images were acquired using an Olympus BX51 microscope (Olympus, Center Valley, PA, USA).

For immunocytofluorescence, cells were seeded on polylysine-coated glass. Initially, 100 nM Mitotracker (Invitrogen, M7512) was incubated for 30 min at 37 °C. After treatment, the cells were fixed in cold acetone for 5 min, permeabilized with Triton X-100 (Beyotime, P0096) for 10 min, and blocked with 5% BSA for 1 hour at RT. The subsequent steps were identical to immunofluorescence staining in kidney tissues.

### Apoptosis analysis

Terminal deoxynucleotidyl transferase-mediated dUTP nick-end labeling (TUNEL) Assay: The assessment of apoptosis in renal tissue was determined using TUNEL BrightGreen Apoptosis Detection Kit (Vazyme Biotechnology, A112-01) according to the manufacturer’s instructions. Briefly, mouse kidney tissues were fixed in 4% paraformaldehyde, embedded in paraffin, and transversely sectioned to a thickness of 3 μm for renal pathology slides. After deparaffinization, renal sections were washed three times with PBS and incubated with the Proteinase K solution for 20 min. Added 100 µl 1× equilibration buffer and equilibrated at RT for 10–30 min. Prepared the TdT reaction mixture in the dark during equilibration. After equilibration, removed 1 × equilibration buffer, and then dropped 50 μl of TdT reaction mixture on the sample. Incubated the plate at 37 °C for 60 min and washed twice with PBS, and incubated at RT for 5 min each time. Counterstained the sample in the dark with DAPI (Beyotime, P0131). Renal sections of TUNEL expression were photographed with an Olympus BX51 microscope (Olympus, Center Valley, PA, USA), and the signals were analyzed using Image J software (RRID: SCR_003070).

Annexin V-FITC/PI staining: The assessment of apoptosis in the renal tubular cells was determined using Annexin V-FITC Apoptosis Detection Kit (BD Biosciences, 556547) according to the manufacturer’s instructions. After transfection with Slc25a21 plasmids and empty vector following cisplatin treatment, the cells were washed with PBS twice and then double-stained with FITC and PI. Analyzed by flow cytometry within 1 h.

### Mitochondria and cytosol separation

Mitochondria were isolated from mouse kidney tissues using the Minute^TM^ Mitochondria Isolation Kit for Mammalian Cells and Tissues (Invent, MP-007) following the manufacturer’s instructions. Briefly, kidney tissues were homogenized in 250 μl buffer A, centrifuged at 16,000 × *g* for 30 s. The filter was discarded, and the pellet was resuspended, followed by centrifugation at 700 × *g* for 1 min. The resulting supernatant was carefully transferred to a fresh 1.5 ml tube and then centrifuged at 16,000 × *g* for 30 min. The obtained supernatant is the cytosol for downstream detection. For mitochondria isolation, after the initial centrifugation at 700 × *g* for 1 min, 300 μl of buffer B was added to the tube, followed by centrifugation at 16,000 × *g* for 30 min. The supernatant was discarded, and the pellet was resuspended in 200 μl of buffer B, followed by centrifugation at 8000 × *g* for 5 min. The resulting supernatant was transferred to a fresh 2.0 ml tube; 1.6 ml of cold PBS was added, and the mixture was centrifuged at 16,000 × *g* for 30 min. The supernatant was discarded, and the pellet, representing isolated mitochondria, was retained for further detection.

### Untargeted metabolomics analysis

This assay was carried out in PANOMIX company, Suzhou, China. Accurately weighed an appropriate amount of kidney tissues from mice, added 200 μl 50% acetonitrile solution containing 2-Amino-3-(2-chloro-phenyl)-propionic acid, prepared for LC-MS detection. The LC analysis was conducted on a Vanquish UHPLC System (Thermo Fisher Scientific, USA). For LC-ESI ( + )-MS analysis, the mobile phases comprised 0.1% formic acid in acetonitrile (v/v) and 0.1% formic acid in water (v/v). Mass spectrometric detection of metabolites was performed on Orbitrap Exploris 120 (Thermo Fisher Scientific, USA) with ESI ion source. Simultaneous MS1 and MS/MS (Full MS-ddMS2 mode, data-dependent MS/MS) acquisition was employed. Raw data were initially converted to mzXML format using MS Convert in ProteoWizard software package (v3.0.8789) and processed with XCMS for feature detection, retention time correction, and alignment. Ropls software was employed for all multivariate data analyses and modeling. Prior to the actual principal component analysis (PCA), data matrices were pre-processed using the Pareto scaling. Orthogonal partial least squares discriminant analysis (OPLS-DA) employing the software SIMCA (v. 13.0, 2011, Umetrics, Umea, Sweden) was performed. OPLS-DA facilitated the identification of different metabolites through variable importance on projection (VIP) analysis. VIP > 1 were statistically significant metabolites. The identified differential metabolites were subjected to pathway analysis by MetaboAnalyst 5.0.

### Detection of levels of 2-oxoadipate and TCA cycle intermediates (citrate, fumarate and malate) in the mitochondria and cytoplasm

This assay was carried out in BIOTREE accompany, Shanghai, China. Mitochondria and cytoplasm were isolated from mouse kidney tissues using the Minute^TM^ Mitochondria Isolation Kit for Mammalian Cells and Tissues (Invent, MP-007) following the manufacturer’s instructions, respectively. After the extraction of metabolites from mitochondria and cytoplasm, and preparation of standard solutions, all samples and calibration solutions were detected by HPIC-MS/MS. The HPIC separation was performed using a Dionex ICS-6000 HPIC System (Thermo Fisher Scientific, USA). This assay utilized a Triple Quad 6500+ System (AB SCIEX) equipped with an electrospray ionization (ESI) interface. The injection volume was set at 5 μl. For each target compound, the ion pair with the highest signal intensity in terms of the parent ion-daughter ion was chosen for qualitative analysis. The Y-axis of the calibration curve represented the peak area of the target compound, while the X-axis represented the concentration of the target compound (µmol/l). The concentration of the target metabolite per milligram of kidney tissue (µmol/l/mg): C_Final metabolite_ =C_Target metabolite_×V_Final extraction_/V_Injection_. The ratio of 2-oxoadipate and TCA cycle intermediates (citrate, fumarate and malate) between the mitochondria and the cytoplasm was calculated by dividing the mitochondrial concentrations by the respective cytoplasmic concentrations.

### Datasets used for the analysis

Three bulk-RNA seq datasets were used in this study. Bulk-RNA seq data (GSE30718) [28] contained 26 AKI patients and 11 healthy individuals. The GSE30122 [29] were derived from human renal tubular or glomerular *SLC25A21* expression from 12 and 10 healthy subjects, respectively. It also contained the information of glomerular filtration rate (GFR) for each sample. The GSE145085 [30] was derived from human iPSC-derived kidney organoids treated with/without cisplatin. In addition, we used a mouse kidney single-cell RNA sequencing dataset (GSE197266) [31], including 2 cisplatin-induced AKI mice and 2 normal control mice. The original cell annotation provided by authors was used for downstream analysis.

### Statistical analysis

All data are presented as means ± standard error of the mean (SEM) using GraphPad Prism 10.0 (GraphPad Software, RRID:SCR_002798). One-way analysis of variance (ANOVA) or unpaired Student’s *t* test (2-tailed) were employed to test for significant differences as indicated. *P* < 0.05 was considered statistically significant, unless otherwise indicated.

## Results

### Decreased renal expression of SLC25A21 is associated with AKI and cisplatin-induced kidney damage

**Fig. 1:**
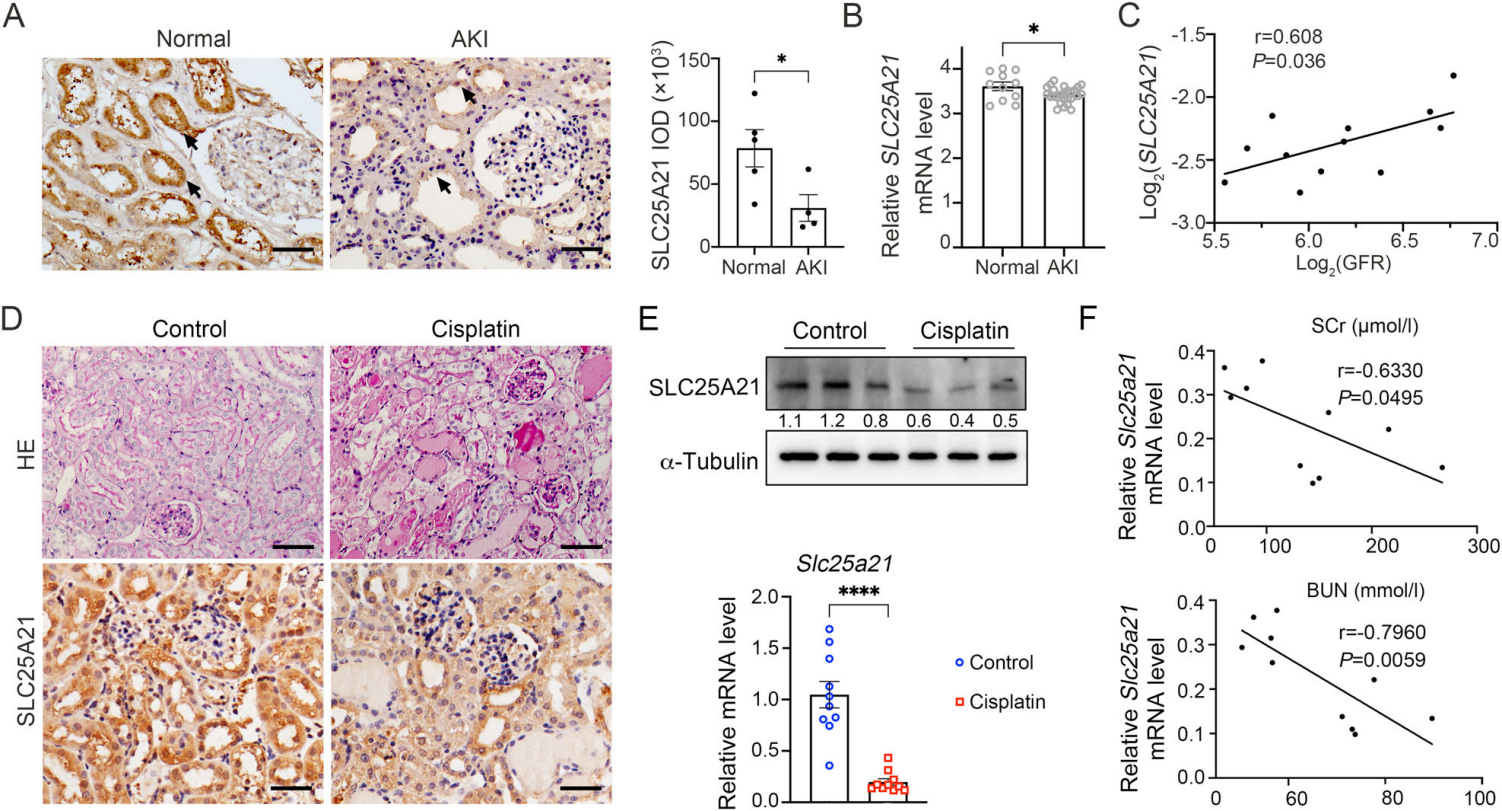
Decreased renal expression of SLC25A21 is associated with AKI and cisplatin-induced kidney damage. **A** Representative immunohistochemistry images of SLC25A21 from human kidney biopsy samples with AKI (*left)* and semi-quantitative IOD analysis of SLC25A21 expression (*right*). The details of AKI patients (*n* = 4) were described in Table S1, and normal samples from para-kidney carcinoma kidney tissues (*n* = 5) were served as controls. Arrows indicates renal tubules. IOD, integral optical density. Scale bars, 50 µm. **B** The mRNA levels of *SLC25A21* in the kidney from both AKI patients and healthy controls (data were derived from GSE30718) [28]. **C** Pearson correlation between tubular *SLC25A21* expression and glomerular filtration rate (GFR) (derived from GSE30122) [29]. **D** Representative Periodic Acid-Schiff (PAS) staining images from cisplatin-induced AKI and control mice, illustrating tubular dilatation and atrophy (*upper*). Scale bars, 50 µm. Representative immunohistochemistry images of SLC25A21 from cisplatin-induced AKI and control mice (*down*). Scale bars, 50 µm. **E** The expression of Slc25a21 in the kidneys from cisplatin-induced AKI and control mice. Representative western blotting for Slc25a21 protein levels (*upper*); *Slc25a21* mRNA expression changes were determined by RT-qPCR (*down*). *n* = 10, each group, and values are reported as mean ± SEM. **F** Pearson correlation analysis between *Slc25a21* mRNA expression relative to control in the kidney and Serum creatinine (SCr, *upper*), as well as Blood Urea Nitrogen (BUN, *down*) in cisplatin-induced AKI mice (*n* = 10). In each case, data were presented as means ± SEM and statistical significance is assessed by the unpaired Student’s t-test. * indicates *P* < 0.05; *****indicates *P* < 0.0001 compared between two groups. See also Fig. S1.

To evaluate the association between SLC25A21 and AKI, we first examined the expression of SLC25A21 in the human kidney (Fig. 1A). Immunohistochemistry (IHC) staining shows a clear distribution of SLC25A21 in normal kidneys, especially within the renal tubules, and SLC25A21 was significantly diminished in AKI kidneys (*P* < 0.05, Fig. 1A). Bulk-RNA sequencing analysis confirmed the reduction in renal *SLC25A21* expression in AKI compared with controls (*P* < 0.05, Fig. 1B) [28]. A significant reduction in *SLC25A21* mRNA expression was further detected in cisplatin-treated kidney organoids compared to normal controls (Figure S1A) [30]. Moreover, the tubular expression of *SLC25A21* in the kidney was positively correlated with the levels of glomerular filtration rate (GFR) (r = 0.608; *P* = 0.036, Fig. 1C), while glomerular *SLC25A21* expression was not correlated with GFR levels (Fig. S1B) [29].

We constructed cisplatin-induced AKI mice models, which showed kidney dysfunction with severe tubular dilatation and epithelial cells degeneration (Fig. 1D*upper*). Cisplatin administration also resulted in pronounced cell apoptosis, necroptosis, and increased inflammatory response in cisplatin-injured kidneys compared to controls (Fig. S1C-I). Furthermore, cisplatin-injured kidneys presented a significant down-regulation of SLC25A21 expression in both protein and mRNA levels (Fig. 1D*down* and Fig. S1J; Fig. 1E). The protein and mRNA level of *Slc25a21* was negatively correlated with kidney function parameters (Serum Creatinine (SCr) and Blood Urea Nitrogen (BUN)) in the cisplatin-induced AKI mouse models (Fig. 1F, Fig. S1K).

### AAV9-sustaining Slc25a21 renal expression attenuated cisplatin-induced AKI

**Fig. 2:**
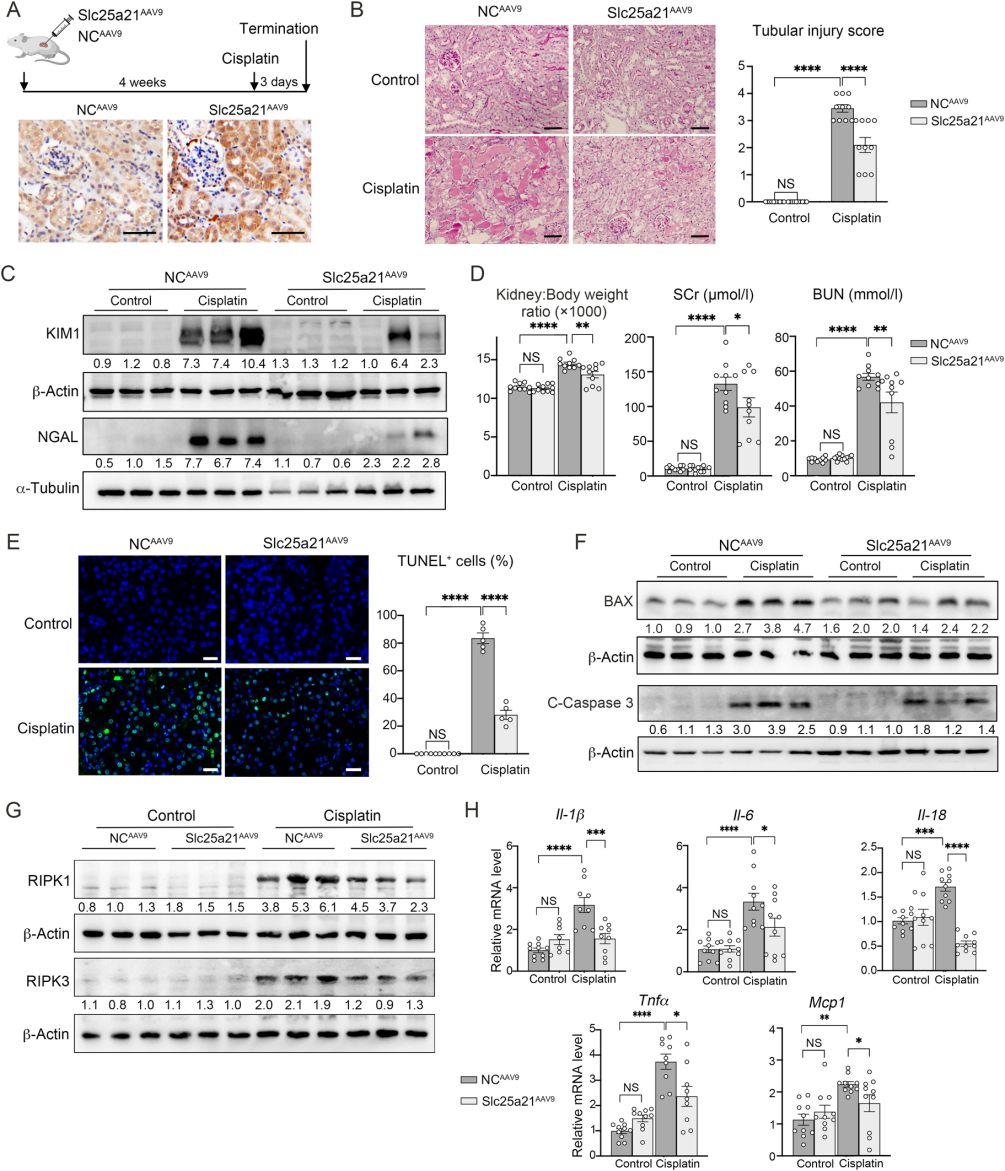
AAV9-sustaining Slc25a21 renal expression attenuated cisplatin-induced AKI. **A** Experimental schematic of mice receiving AAV9-Slc2521 (Slc25a21^AAV9^) or AAV9-scramble (NC^AAV9^) in situ injection in the kidneys, followed by cisplatin treatment (25 mg/kg) for three days to induce AKI (*upper*). Representative immunohistochemistry images of Slc25a21-targeted HA from Slc25a21^AAV9^ and NC^AAV9^ mice (*down*). Scale bars, 50 µm. **B** Representative Periodic Acid-Schiff (PAS) staining image in Slc25a21^AAV9^ and NC^AAV9^ mice following cisplatin or saline injection. *left:* Representative PAS staining for AKI lesions in the kidneys. Scale bars, 50 µm. *right:* semi-quantitative analysis of tubular injury score in the kidneys. *n* = 10, each group and values are reported as mean ± SEM. **C** Representative western blotting for KIM1 and NGAL protein levels in Slc25a21^AAV9^ and NC^AAV9^ mice after cisplatin or saline injection. **D** The ratio of kidney to body weight and concentrations of Serum SCr and BUN in Slc25a21^AAV9^ and NC^AAV9^ mice after cisplatin or saline injection. *n* = 10, each group, and values are reported as mean ± SEM. **E** Representative immune images for TUNEL expression in Slc25a21^AAV9^ and NC^AAV9^ mice after cisplatin or saline injection (*left*), with quantification of TUNEL-positive cells per field (*right*). 200× field images were recorded to calculate the mean for each mouse kidney, represented with one dot. *n* = 5, each group and values are reported as mean ± SEM. Scale bars, 50 µm. **F** Representative western blotting for BAX and Cleaved-Caspase 3 (C-Caspase 3) expression level in the kidneys from both Slc25a21^AAV9^ and NC^AAV9^ mice after cisplatin or saline injection. **G** Representative western blotting for RIPK1 and RIPK3 expression level in the kidneys from both Slc25a21^AAV9^ and NC^AAV9^ mice after cisplatin or saline injection. **H** Expression of inflammatory markers (*Il-1β, Il-6*, *Il-18, Tnfα* and *Mcp1*), determined by RT-qPCR, in the kidneys from Slc25a21^AAV9^ and NC^AAV9^ mice after cisplatin or saline injection. *n* = 9–10, each group, and values are reported as mean ± SEM. In each case, data were presented as means ± SEM and statistical significance is assessed by One-way ANOVA analysis of variance of Tukey’s multiple comparisons test. * indicates *P* < 0.05; ** indicates *P* < 0.01; *** indicates *P* < 0.001; **** indicates *P* < 0.0001; NS indicates not significant. See also Fig. S2, S3.

To elucidate the role of Slc25a21 in the kidney, we used the AAV9 system with kidney multi-point in situ injection in the renal cortex (Slc25a21^AAV9^, Fig. 2A). Compared with AAV9-scramble (a scramble sequence) controls (NC^AAV9^), AAV9-Slc25a21 delivery enhanced Slc25a21 expression in renal tubules, tracked through IHC staining with SLC25A21-targeted HA (Fig. 2A, Fig. S2A), and partially restored the expression of Slc25a21 in AKI kidneys (Fig. S2B, C). Furthermore, Slc25a21^AAV9^ cisplatin-induced AKI mice showed decreased tubular dilatation and epithelial cells atrophy, which was associated with a lower tubular injury score (Fig. 2B). These mice also exhibited significantly decreased expression of kidney injury molecule 1 (KIM1) and Neutrophil gelatinase associated lipocalin (NGAL) (Fig. 2C, Fig. S2D), lower kidney to body weight ratio, and decreased levels of SCr and BUN (Fig. 2D), suggesting a functional improvement in tubular injury and kidney damage with respect to NC^AAV9^ AKI mice. Both levels of SCr and BUN were strongly negatively correlated with the expression of Slc25a21 in cisplatin-induced AKI mouse kidneys following AAV application (Fig. S2E), with correlation coefficients of r = –0.92 (*P* = 0.0004) and r = –0.76 (*P* = 0.0176), respectively. These findings suggest that maintaining the expression of Slc25a21 is associated with protection against cisplatin-induced kidney dysfunction.

Slc25a21^AAV9^ mice exhibited decreased cell apoptosis in the kidneys following cisplatin injection compared to NC^AAV9^ AKI mice, as shown by the lower TUNEL-positive cells (Fig. 2E) and expression of BAX and Cleaved-Caspase 3 (Fig. 2F, Fig. S2F). In addition to cell apoptosis, we also evaluated the necroptosis in cisplatin-induced AKI. The protein expression of necroptosis markers RIPK1 and RIPK3 was significantly reduced in Slc25a21^AAV9^ AKI mice compared to NC^AAV9^ AKI mice (Fig. 2G, Fig. S2G). The inflammatory response was also attenuated in the Slc25a21^AAV9^ AKI kidney, which had lower renal expression of Interleukin-1 (IL-1β), Interleukin-6 (IL-6), Interleukin-18 (IL-18), Tumor necrosis factor α (TNFα) and Monocyte chemoattractant protein 1 (MCP1) than NC^AAV9^ mice after cisplatin treatment (Fig. 2H), which was mirrored by a reduction in circulating IL-18 and TNFα levels (Fig. S2H). We used another systemic delivery method of Slc25a21 by high-pressure tail-vein injection of plasmids (Slc25a21^DNA^), which confirmed that maintaining Slc25a21 expression attenuates cisplatin-induced renal injury and dysfunction, as well as tubular apoptosis, necroptosis, and inflammatory response (Fig. S3). Thus, preservation of Slc25a21 expression protects against cisplatin-induced AKI in mice.

### Rescuing Slc25a21 ameliorates cisplatin-induced acute tubular injury in vitro

**Fig. 3:**
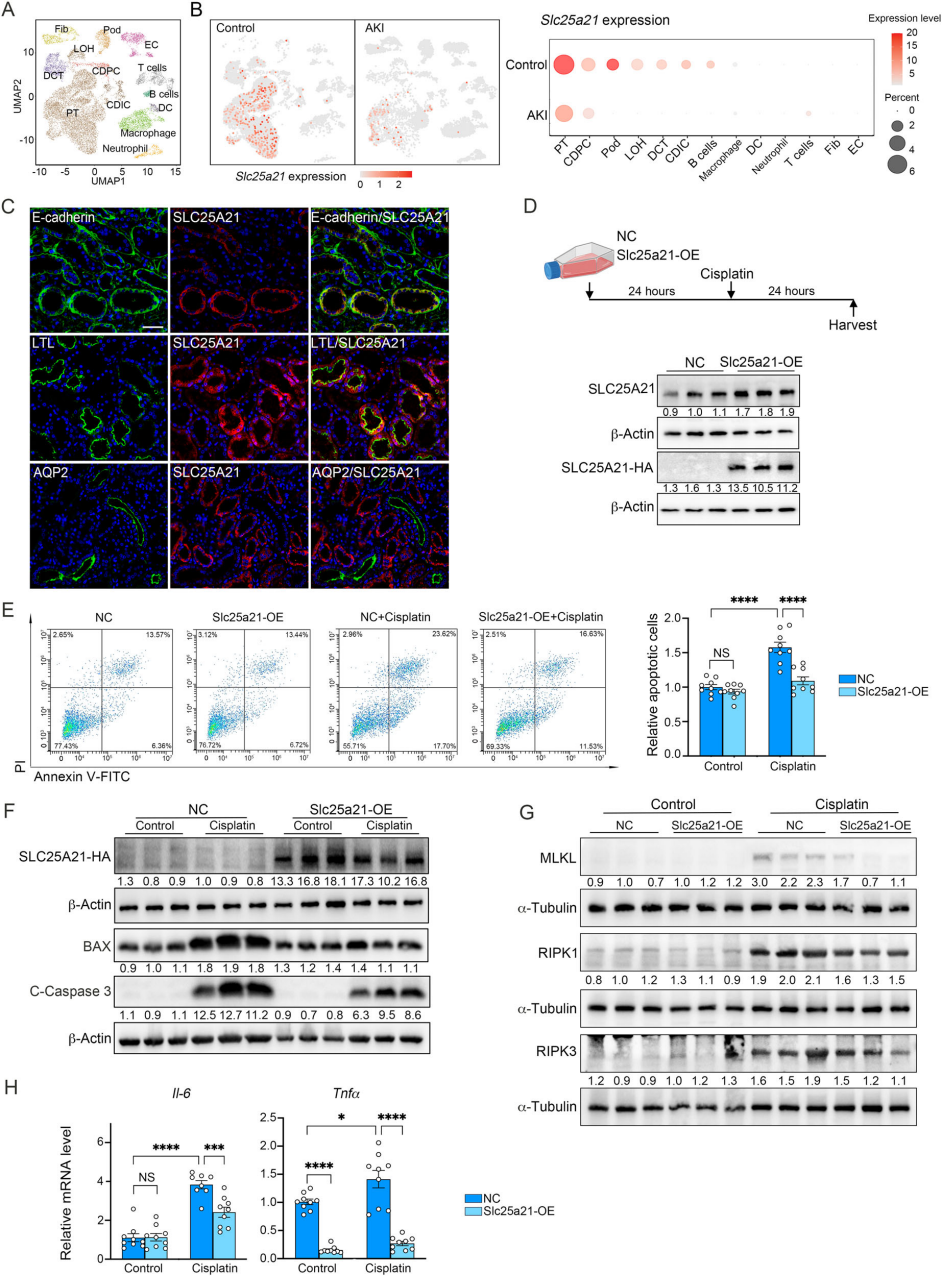
Rescuing Slc25a21 ameliorates cisplatin-induced acute tubular injury in vitro. **A** Uniform manifold approximation and projection (UMAP) plots of cisplatin-induced AKI datasets based on single-cell RNA sequencing showing 13 subclusters of kidney tissue (GSE197266) [31]. **B** The expression of Slc25a21 in each subcluster in cisplatin-induced AKI and control mice (*left*) and Slc25a21 was mainly expressed in proximal renal tubular cells (*right*). T cells T lymphocytes, B cells B lymphocytes, EC endothelial cells, DC dendritic cells, PT proximal renal tubular cells, LOH Loop of Henle cells, DCT Distal convoluted tubule cells, CDIC collecting duct-intercalated cells, CDPC: collecting duct-principal cells, Pod podocyte, Fib fibroblasts. **C** Representative immunofluorescence image depicting co-staining of SLC25A21 with E-cadherin, Lotus tetragonolobus lectin (LTL), AQP2 in the human kidneys. Green presents tubular makers; red presents SLC25A21; blue presents DAPI. Scale bars, 50 µm. **D** Experimental schematic of Slc25a21-OE or normal control (NC) plasmids transfection into the TKPTS cells for 24 h, exposed by cisplatin (10 µM) for 24 h (*upper*). Representative western blotting for Slc25a21 and Slc25a21-tagged HA protein levels (*down*). **E** Representative flow cytometry images of cellular apoptosis in Slc25a21-OE and NC cells after cisplatin (10 µM) treatment for 24 h (*left*). Relative apoptotic cells (including apoptotic and even dead cells, *right*). *n* = 9, each group, and values are reported as mean ± SEM. **F** Representative western blotting for Slc25a21-tagged HA, BAX and C-Caspase 3 protein expression in Slc25a21-OE and NC cells after cisplatin (10 µM) treatment for 24 h. C-Caspase 3: Cleaved Caspase 3. **G** Representative western blotting for MLKL, RIPK1 and RIPK3 protein expression in Slc25a21-OE and NC cells after cisplatin (10 µM) treatment for 24 h. **H** Expression of inflammatory markers (*Il-6* and *Tnfα*) in TKPTS cells, determined by RT-qPCR, in Slc25a21-OE and NC cells after cisplatin (10 µM) treatment for 24 h. *n* = 8–9, each group, and values are reported as mean ± SEM. In each case, data were presented as means ± SEM and statistical significance is assessed by One-way ANOVA analysis of variance of Tukey’s multiple comparisons test. * indicates *P* < 0.05; *** indicates *P* < 0.001; **** indicates *P* < 0.0001; NS indicates not significant. See also Fig. S4.

Our data suggested that renal overexpression of Slc25a21 mitigates tubular injury in AKI (Fig. 2B); we therefore investigated whether renal tubular cells were directly regulated by Slc25a21. We first showed the distribution of Slc25a21 in the kidney and analyzed a public single-cell RNA sequencing (ScRNA-seq) dataset derived from cisplatin-induced AKI kidneys (Fig. 3A) [31]. Slc25a21 was mainly enriched in the tubular cells, including proximal tubules (PT), distal convoluted tubule cells (DCT) and Loop of Henle cells (LOH). Among these cell-types, Slc25a21 was primarily expressed in the PT cell compartment. Compared with controls, Slc25a21 expression was dramatically decreased in PT derived from AKI kidneys (Fig. 3B). The immunofluorescence co-staining Lotus tetragonolobus lectin (LTL, a marker for proximal renal tubules), Aquaporin 2 (AQP2, a marker for collecting ducts) and E-cadherin for renal tubules with SLC25A21, further confirmed the renal PT localization of SLC25A21. SLC25A21 is expressed in some tubular cells (Fig. 3C*upper*), mostly in the LTL positive cells (proximal tubular cells), but rarely in the AQP2 positive tubules (collecting ducts). In addition, following AAV9 administration exogenous SLC25A21 presents a similar distribution with native protein of SLC25A21 (Fig. S4A).

Using an in vitro AKI model of kidney proximal tubular epithelial cells (TKPTS), we showed a dose- and time-dependent reduction in Slc25a21 expression in tubular epithelial cells after cisplatin treatment (Fig. S4B, C). To further explore the role of Scl25a21 in tubular cells, we increased the expression of Slc25a21 in these cisplatin-induced TKPTS cells through transfection with Slc25a21 plasmids (Slc25a21-OE, Fig. 3D, Fig. S4D). Rescue of Slc25a21 expression significantly increased tubular cell viability following cisplatin treatment (Fig. S4E). Compared to normal control (NC) cells, we also observed a decreased proportion of apoptotic cells (Fig. 3E) and reduced expression of apoptosis markers (e.g. BAX and C-Caspase 3), as well as necroptosis markers (e.g. MLKL, RIPK1 and RIPK3) after cisplatin induction (Fig. 3F, G, Fig. S4F, G). The mRNA expression of inflammatory markers, *Il-6* and *Tnfα*, was also reduced upon Slc25a21 plasmids transfection (Fig. 3H). Taken together, these data show that tubular cells are a major cell type in the kidney regulated by Slc25a21 during AKI, and Slc25a21 directly modulates cellular apoptosis, necroptosis and inflammatory response following cisplatin-induced acute tubular injury.

### Slc25a21 is important for preserving mitochondrial homeostasis during AKI

**Fig. 4:**
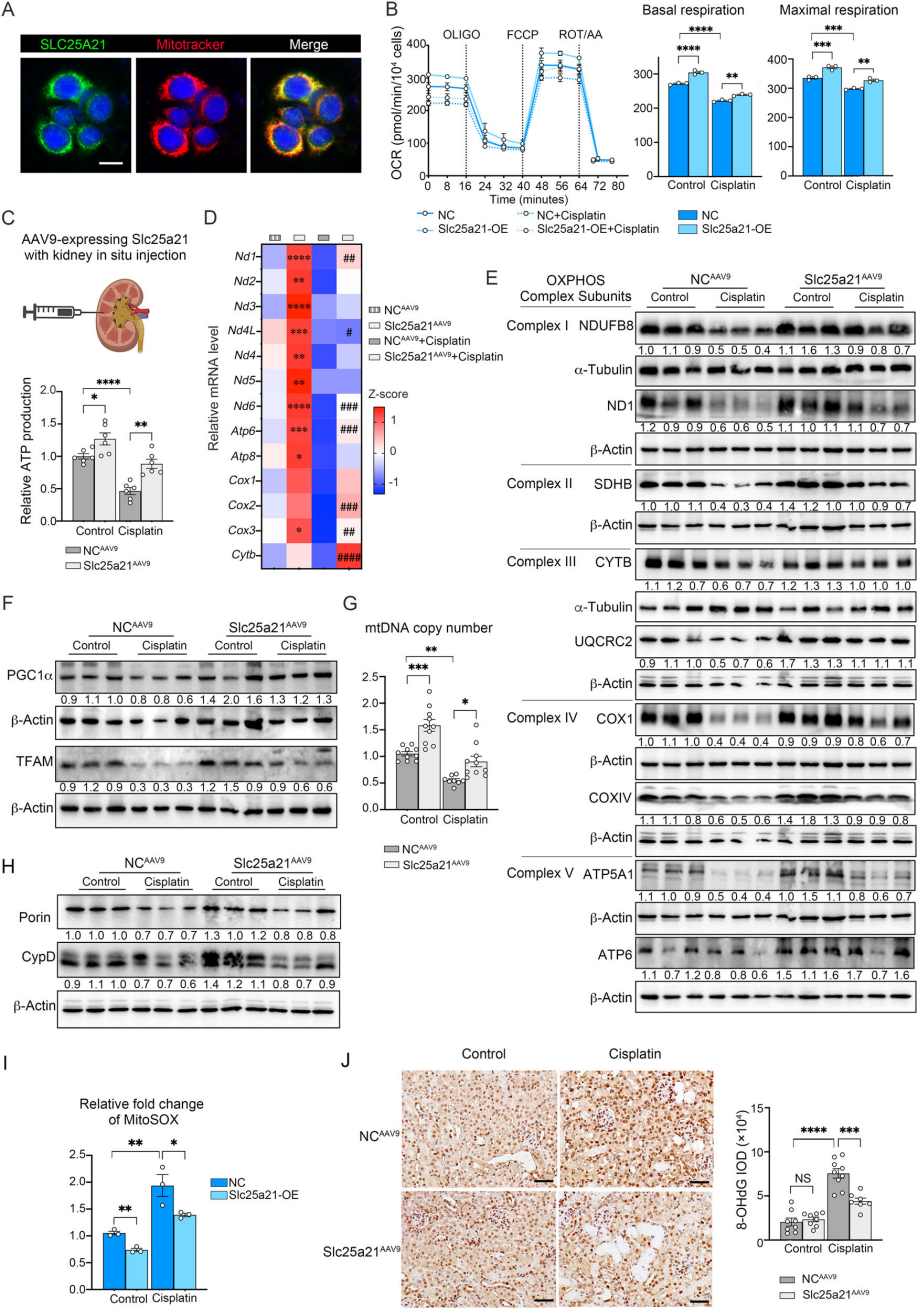
Slc25a21 is important for preserving mitochondrial homeostasis during AKI. **A** Representative immunofluorescence image of co-staining of SLC25A21 with Mitotracker revealed the mitochondrial localization of SLC25A21 in TKPTS cells. Green presents SLC25A21; red presents Mitotracker; blue presents DAPI. Scale bars, 20 µm. **B** The rate of oxygen consumption was assessed using the Seahorse XF-96 Extracellular Flux Analyzer in Slc25a21-OE and normal control (NC) cells after cisplatin (10 µM) treatment for 24 h (*left*). OLIGO oligomycin, FCCP Carbonyl cyanide 4-(trifluoromethoxy)phenylhydrazone, ROT rotenone, AA antimycin A. Oxygen consumption rate for basal and maximal respiration was analyzed (*right*). *n* = 6, each group, and values are reported as mean ± SEM. **C** Relative ATP production in the kidneys of Slc25a21^AAV9^ and NC^AAV9^ mice after cisplatin or saline addition. *n* = 6, each group, and values are reported as mean ± SEM. **D** Heatmap showing mRNA expression of the 13 mitochondrially encoded genes in Slc25a21^AAV9^ and NC^AAV9^ mice after cisplatin or saline addition, determined by RT-qPCR (*n* = 10). Data are presented as Z-score. *indicates Slc25a21^AAV9^ *vs* NC^AAV9^, #indicates Slc25a21^AAV9^+Cisplatin vs NC^AAV9^+Cisplatin. **E** Representative western blotting for protein expression levels of enzymes involved in mitochondrial OXPHOS in the kidneys of Slc25a21^AAV9^ and NC^AAV9^ mice after cisplatin or saline addition. NDUFB8 NADH dehydrogenase (ubiquinone) 1 beta subcomplex subunit 8, ND1 NADH-ubiquinone oxidoreductase chain 1, SDHB Succinate dehydrogenase (ubiquinone) iron-sulfur subunit, CYTB Cytochrome b; UQCRC2 Cytochrome b-c1 complex subunit 2, COX1 Cytochrome c oxidase subunit 1, COX IV cytochrome c oxidase subunit 4 isoform 1, ATP5A1 ATP synthase subunit alpha, ATP6 ATP synthase subunit a. **F** Representative western blotting for PGC1α and TFAM protein expression levels of enzymes associated with mitochondrial biogenesis in the kidneys of Slc25a21^AAV9^ and NC^AAV9^ mice after cisplatin or saline addition. **G** The mtDNA copy number in the kidneys of Slc25a21^AAV9^ and NC^AAV9^ mice after cisplatin or saline addition measured by qRT-PCR. *n* = 8–10, each group, and values are reported as mean ± SEM. **H** Representative western blotting for Porin and CypD protein expression levels of enzymes associated with mitochondrial integrity in the kidneys of Slc25a21^AAV9^ and NC^AAV9^ mice after cisplatin or saline addition. CypD: cyclophilin D. **I** Relative fold change of MitoSOX detected in Slc25a21-OE and NC cells after cisplatin (10 µM) treatment for 24 h. *n* = 3, each group, and values are reported as mean ± SEM. **J** Representative immunohistochemistry images of 8-OHdG expression in the kidneys of Slc25a21^AAV9^ and NC^AAV9^ mice after cisplatin or saline addition (*left*) and semi-quantitative IOD analysis of 8-OHdG expression (*right*). *n* = 7–9, each group, and values are reported as mean ± SEM. Scale bars, 50 µm. IOD integral optical density. In each case, data were presented as means ± SEM and statistical significance is assessed by One-way ANOVA analysis of variance of Tukey’s multiple comparisons test. * indicates *P* < 0.05; ** indicates *P* < 0.01; *** indicates *P* < 0.001; **** indicates *P* < 0.0001; # indicates *P* < 0.05; ## indicates *P* < 0.01; ### indicates *P* < 0.001; #### indicates *P* < 0.0001; NS indicates not significant. See also Fig. S5.

Consistent with previous reports that identified Slc25a21 as a mitochondrial carrier in yeast and human cells [23, 24], we showed the mitochondrial sub-cellular localization of Slc25a21 in the tubular cells (TKPTS) by Mitotracker (Fig. 4A). Cisplatin-treated tubular cells showed reduced Slc25a21 expression in the mitochondria (Fig. S5A). We then investigated the potential mitochondrial function of Slc25a21 in tubular epithelial cell, as well as its role in AKI.

Maintaining Slc25a21 expression was associated with preserved mitochondrial respiratory function in Slc25a21-OE tubular cells upon cisplatin treatment, when compared with NC cells (Fig. 4B). AAV9-Slc25a21 administration in the kidney partially restored impaired ATP production in cisplatin-induced AKI kidneys (Fig. 4C). Together with the respiratory dysfunction during AKI, there was a decrease in the renal expression of 13 mitochondrial genes involved in the oxidative phosphorylation (OXPHOS) complex, impacting all OXPHOS subunits (Fig. 4D, E, Fig. S5B). Following cisplatin treatment, maintaining Slc25a21 expression preserved the expression of these OXPHOS genes at both mRNA and protein levels (Fig. 4D, E, Fig. S5B). Similarly, expression of these mitochondrial OXPHOS genes was restored in AKI kidney by high-pressure tail-vein injection of Slc25a21 plasmids (Fig. S5C, D) and by transfection of Slc25a21 plasmids in acutely injured TKPTS cell lines (Fig. S5E).

In addition to respiratory ATP synthesis, mitochondrial biogenesis and integrity are also important for mitochondrial homeostasis. Peroxisome proliferator-activated receptor gamma co-activator-1 alpha (PGC1α) and mitochondrial transcription factor A (TFAM) are two key proteins involved in mitochondrial biogenesis pathway [13, 32]. The expression of PGC1α and TFAM in the kidney is significantly suppressed by cisplatin injection, and rescued by Slc25a21 expression induced with renal AAV9 administration (Fig. 4F, Fig. S5F). The reduction in mitochondrial DNA (mtDNA) copy number in AKI kidneys was also ameliorated by maintaining Slc25a21 expression (Fig. 4G). Porin and cyclophilin D (CypD) are components of the mitochondrial outer membrane and mediators of the mitochondrial permeability transition pore, which play a significant role in maintaining mitochondrial integrity. The expression levels of Porin and CypD in the kidneys were reduced following cisplatin-induced AKI, which was rescued by AAV9-Slc25a21 injection (Fig. 4H, Fig. S5G).

Additionally, the disturbance of mitochondrial homeostasis was accompanied by the release of reactive oxygen species (ROS) in AKI (Fig. 4I-J). MitoSOX staining showed that mitochondrial ROS levels in TKPTS cells were significantly increased following cisplatin treatment, and in vitro rescue of Slc25a21 suppressed oxidative stress in these tubular cells (Fig. 4I). The levels of 8-OHdG, a biomarker of oxidative DNA damage, were increases in AKI kidneys, and alleviated by AAV9-Slc25a21 intervention (Fig. 4J).

### Slc25a21 facilitates mitochondrial 2-oxoadipate transport and metabolism

**Fig. 5:**
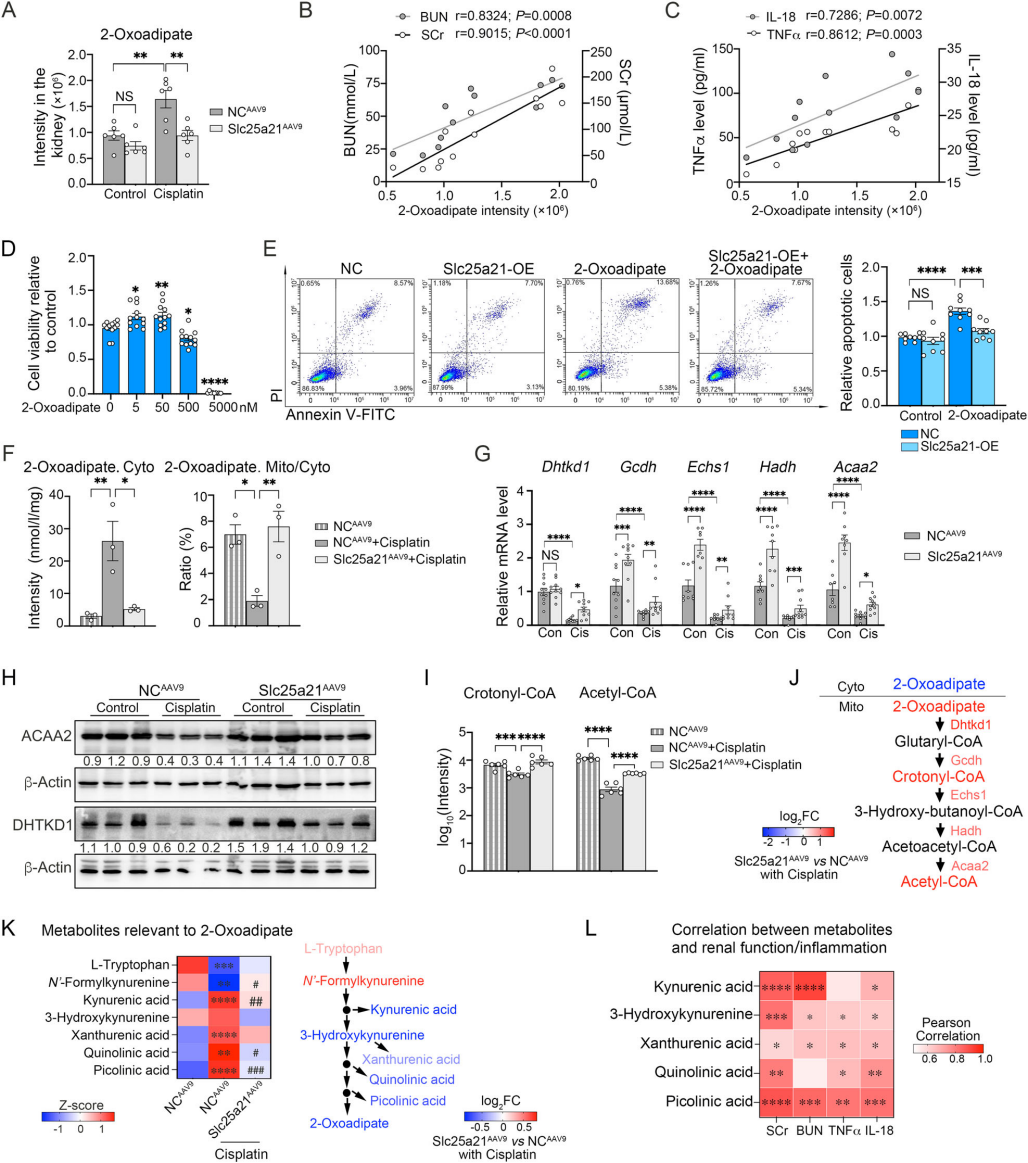
Slc25a21 facilitates mitochondrial 2-oxoadipate transport and metabolism. **A** Assessment of 2-oxoadipate level in the kidneys of Slc25a21^AAV9^ and NC^AAV9^ mice after cisplatin or saline addition, determined by HPLC-MSMS. *n* = 6, each group, and values are reported as mean ± SEM. **B** Pearson correlation analysis between 2-oxoadipate intensity and the concentrations of SCr as well as BUN in the kidneys of Slc25a21^AAV9^ and NC^AAV9^ mice after cisplatin addition (*n* = 12). **C** Pearson correlation analysis between 2-oxoadipate intensity and the concentrations of TNFα as well as IL-18 in the kidneys of Slc25a21^AAV9^ and NC^AAV9^ mice after cisplatin addition (*n* = 12). **D** Relative cell viability detected by TKPTS cells exposed to a different gradient of 2-oxoadipate concentrations for 24 h. *n* = 9–12, each group, and values are reported as mean ± SEM. * indicates 2-oxoadipate-treated TKPTS cells *vs* normal control cells. **E** Representative flow cytometry images of cellular apoptosis measured in Slc25a21-OE and normal control (NC) cells after 2-oxoadipate (500 nM) addition for 24 h (*left*) and relative apoptotic cells to control (including apoptotic and even dead cells, *right*). *n* = 9, each group, and values are reported as mean ± SEM. **F** The intensity of 2-oxoadipate in the cytoplasm (*left*) and the ratio of 2-oxoadipate between the mitochondria and the cytoplasm in Slc25a21^AAV9^ and NC^AAV9^ mice after cisplatin or saline addition, determined by HPIC-MSMS (*right*). *n* = 3, each group, and values are reported as mean ± SEM. **G**, **H** The expression of enzymes related to 2-oxoadipate metabolism in the kidneys of Slc25a21^AAV9^ and NC^AAV9^ mice after cisplatin or saline addition. **G** mRNA expression changes were determined by RT-qPCR. *n* = 8–10, each group, and values are reported as mean ± SEM. *Dhtkd1*: Dehydrogenase E1 and transketolase domain containing 1; *Gcdh*: Glutaryl-CoA dehydrogenase; *Echs1*: Enoyl-CoA hydratase, short chain 1; *Hadh*: Hydroxyacyl-CoA dehydrogenase; *Acaa2*: Acetyl-CoA Acyltransferase 2. (H) Representative western blotting for representative protein (ACAA2 and DHTKD1) levels. **I** The levels of metabolites (crotonyl-CoA and acetyl-CoA) from 2-oxoadipate metabolism were detected by HPLC-MSMS in the kidneys of Slc25a21^AAV9^ and NC^AAV9^ mice after cisplatin or saline addition. *n* = 6, each group, and values are reported as mean ± SEM. **J** Schematic of 2-oxoadipate conservation into acetyl-CoA summarizing the metabolic changes observed Slc25a21^AAV9^ following cisplatin administration compared to normal controls in AKI. **K** Heatmap visualizing differentially expressed metabolites relevant to 2-oxoadipate in Slc25a21^AAV9^ and NC^AAV9^ mice after cisplatin or saline addition, determined by HPLC-MSMS (*left*, *n* = 6). Data are presented as Z-score. *indicates Slc25a21^AAV9^ vs NC^AAV9^, #indicates Slc25a21^AAV9^+Cisplatin vs NC^AAV9^+Cisplatin. Schematic of tryptophan metabolism summarizing the metabolic changes observed Slc25a21^AAV9^ following cisplatin administration compared to normal controls in AKI (*right*). **L** Pearson correlation analysis between metabolites relevant to 2-oxoadipate (Kynurenic acid, 3-Hydroxykynurenine, Xanthurenic acid, Quinolinic acid and Picolinic acid) and the concentrations of SCr, BUN, TNFα as well as IL-18 in Slc25a21^AAV9^ and NC^AAV9^ mice after cisplatin addition (*n* = 12). *indicates *P* values of Pearson correlation. In each case, data were presented as means ± SEM and statistical significance is assessed by One-way ANOVA analysis of variance of Tukey’s multiple comparisons test. * indicates *P* < 0.05; ** indicates *P* < 0.01; *** indicates *P* < 0.001; **** indicates *P* < 0.0001; # indicates *P* < 0.05; ## indicates *P* < 0.01; ### indicates *P* < 0.001; #### indicates *P* < 0.0001; NS indicates not significant. See also Fig. S6.

Given that one of the main functions of Slc25a21 is to transport 2-oxodicarboxylate across inner mitochondrial membrane into mitochondria [23], we speculate that Slc25a21 might regulate the progression of AKI by mediating the influx of 2-oxoadipate into tubular cells. To test this hypothesis, we first assessed the concentration of 2-oxoadipate in AKI kidneys. Cisplatin treatment significantly increased the whole levels of 2-oxoadipate in AKI kidneys, which was suppressed by the rescue of Slc25a21 through AAV9 administration (Fig. 5A). Importantly, high levels of 2-oxoadipate in the kidney positively correlated with AKI-induced kidney dysfunction (Fig. 5B) and pro-inflammatory response (Fig. 5C). Moreover, in vitro exposure to moderate levels of 2-oxoadipate led to increased cell viability in TKPTS cells (Fig. 5D), whereas high 2-oxoadipate concentration (500 nM) resulted in the increased cellular apoptosis and decreased cell viability (Fig. 5D, E). Notably, sustaining Slc25a21 expression significantly attenuated apoptosis in TKPTS cells induced by 2-oxoadipate at high concentration. Specifically, increased 2-oxoadipate levels in cisplatin-induced kidneys were detected mainly in the cytoplasm of tubular cells, and associated with a lower mitochondrial to cytoplasmic ratio (Fig. 5F). However, maintaining the level of Slc25a21 by AAV9 introduction reversed the mitochondrial to cytoplasmic ratio of 2-oxoadipate (Fig. 5F). These findings demonstrate that decreased Slc25a21 expression in AKI hindered the mitochondrial transport of 2-oxoadipate, leading to its cytoplasmic accumulation and resulting in acute kidney injury.

2-oxoadipate can be also converted into acetyl-CoA after entering the mitochondrial matrix [23]. During cisplatin-induced AKI, the expression of representative enzymes responsible for converting 2-oxoadipate to acetyl-CoA was significantly reduced (Fig. 5G), which would be consistent with the reduction in mitochondrial transport of 2-oxoadipate under these conditions (Fig. 5A, F). We observed that the levels of these enzymes were increased following Slc25a21 administration via AAV9, such as DHTKD1 and ACAA2 (Fig. 5G, H, Fig. S6A). Consequently, AAV9-Slc25a21 administration also increased the levels of acetyl-CoA in AKI kidneys compared to AKI controls, together with crotonyl-CoA, another intermediary metabolite derived from the conversion of 2-oxoadipate into acetyl-CoA (Fig. 5I). The regulation of Slc25a21 on 2-oxoadipate conversation was confirmed in a separate in vivo AKI kidney model (following high-pressure tail-vein injection of Slc25a21 plasmids), and by in vitro acutely injured TKPTS cells (Fig. S6B–D). Thus, these data would be consistent with a mechanism where sustaining Slc25a21 expression rescue the metabolic pathway of 2-oxoadipate following its mitochondrial transport (Fig. 5J).

We also investigated potential differences in the metabolite influx between AAV9-Slc25a21 and AAV9-NC treated-AKI kidneys, and found that several metabolites involved in tryptophan metabolism were regulated by Slc25a21 (Fig. 5K). AKI triggers the upregulation of these metabolites, which was reversed by Slc25a21. The perturbation of these metabolites was shown to be associated with renal dysfunction and inflammatory response in AKI progression (Fig. 5L). Since 2-oxoadipate is an intermediate in the metabolism of tryptophan [23, 25], these findings suggest a role of Slc25a21 in regulating the metabolic pathway that contributes to 2-oxoadipate production.

### TCA cycle enriched in the metabolism regulated by Slc25a21 in AKI

**Fig. 6:**
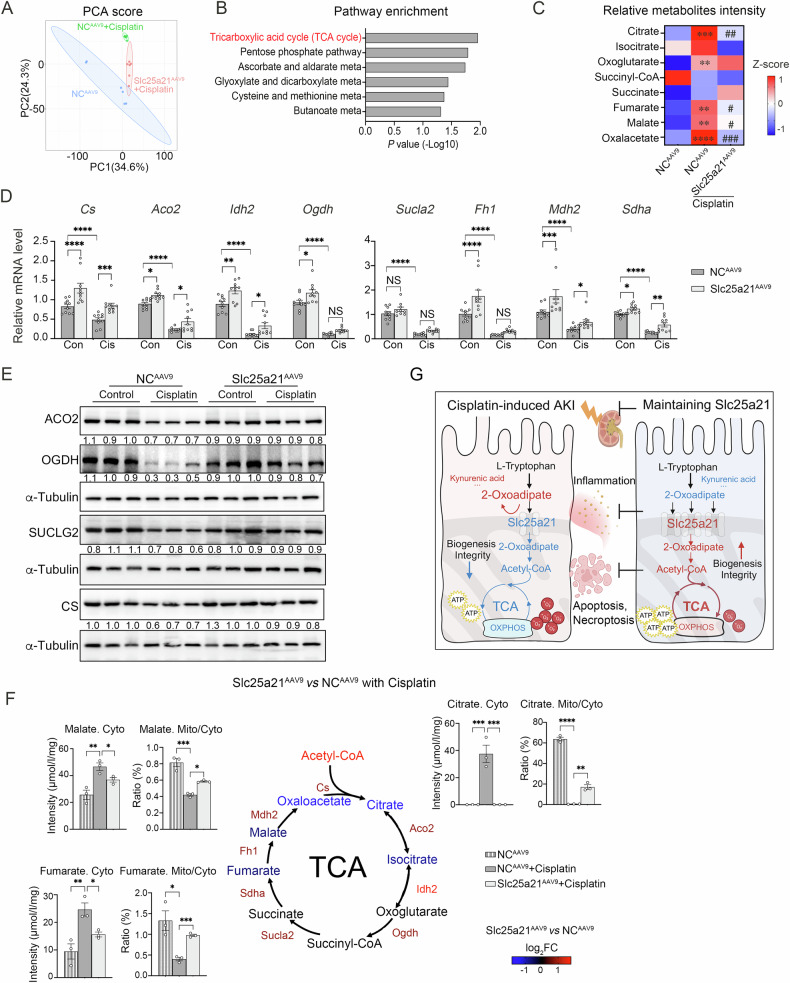
TCA cycle enriched in the metabolism regulated by Slc25a21 in AKI. **A** Principal components analysis (PCA) score depicting metabolite profiles in Slc25a21^AAV9^ and NC^AAV9^ mice after cisplatin or saline administration (*n* = 6). **B** KEGG enrichment analysis of significantly different metabolites (VIP > 1) between Slc25a21^AAV9^ and NC^AAV9^ mice following cisplatin addition analyzed by MetaboAnalyst 5.0. The *P* value of displayed pathway in the figure is less than 0.05 and calculated using MetaboAnalyst 5.0. **C** Heatmap showing differentially expressed metabolites in the TCA cycle in Slc25a21^AAV9^ and NC^AAV9^ mice following cisplatin or saline addition (*n* = 6), determined by HPLC-MSMS. Data are presented as Z-score. *indicates Slc25a21^AAV9^ vs NC^AAV9^, #indicates Slc25a21^AAV9^+Cisplatin vs NC^AAV9^+Cisplatin. **D**, **E** The expression of enzymes associated with the TCA cycle in the kidneys of Slc25a21^AAV9^ and NC^AAV9^ mice following cisplatin or saline addition. **D** mRNA expression changes were determined by RT-qPCR. *n* = 9–10, each group, and values are reported as mean ± SEM. *Cs*: Citrate synthase; *Aco2*: Aconitase 2; *Idh2*: Isocitrate dehydrogenase; *Ogdh*: Oxoglutarate dehydrogenase; *Sucla2*: Succinate-CoA ligase ADP-forming subunit β; *Fh1*: Fumarate hydratase 1; *Mdh2*: Malate dehydrogenase 2; *Sdha*: Succinate dehydrogenase flavoprotein subunit. **E** Representative western blotting for representative protein (ACO2, OGDH, SUCLG2 and CS) levels. **F** The levels of citrate, fumarate and malate in the cytoplasm and the ratio of citrate, fumarate and malate between the mitochondria and the cytoplasm in Slc25a21^AAV9^ and NC^AAV9^ mice following cisplatin or saline addition, determined by HPIC-MSMS. *n* = 3, each group, and values are reported as mean ± SEM. Schematic of TCA cycle summarizing the metabolic changes observed Slc25a21^AAV9^ following cisplatin treatment compared to normal controls in AKI (*middle*). **G** Schematic of proposed mechanism of maintaining Slc25a21 mitigated renal tubular injury induced by cisplatin. Preservation of Slc25a21 expression facilitated the TCA cycle by enhancing 2-oxoadipate transportation and metabolism, improved mitochondrial homeostasis along with elevated ATP synthesis and reduced ROS production, as well as supported mitochondrial biogenesis and integrity, thereby ameliorating cellular apoptosis, necroptosis and inflammatory response. Red indicates up-regulation; blue indicates down-regulation. This figure was created by BioRender website. In each case, data were presented as means ± SEM and statistical significance is assessed by One-way ANOVA analysis of variance of Tukey’s multiple comparisons test. * indicates *P* < 0.05; ** indicates *P* < 0.01; *** indicates *P* < 0.001; **** indicates *P* < 0.0001; # indicates *P* < 0.05; ## indicates *P* < 0.01; ### indicates *P* < 0.001; #### indicates *P* < 0.0001; NS indicates not significant. See also Fig. S7.

To further understand the regulation of Slc25a2 in metabolic profile during AKI, we profiled the global metabolomic changes associated with Slc25a21 and detected 497 metabolites in cisplatin-induced AKI kidneys (Fig. 6A). Principal component analysis (PCA) identified 100 metabolites with variable importance in projection (VIP) scores greater than 1, which can separate Slc25a21 AAV9-treated from control AKI kidneys (Fig. 6A and Table S3). Kyoto Encyclopedia of Genes and Genomes (KEGG) pathway enrichment analysis revealed that these metabolites were significantly associated with energy metabolism pathways, such as the tricarboxylic acid cycle (TCA cycle) and pentose phosphate pathway etc. (Fig. 6B).

We focused on the TCA cycle, which occurs in the mitochondria and provides energy in aerobic conditions together with acetyl-CoA [33]. The TCA cycle is also the major pathway for interconversion of 4- and 6-carbon compounds in the cell, including citrate, fumarate and oxaloacetate [34]. In keeping with the observed changes in TCA cycle metabolites (Fig. 6C), the gene expression of representative enzymes participating in the TCA cycle in AKI kidney was increased in Slc25a21^AAV9^ compared with controls (NC^AAV9^) (Fig. 6D, E, Fig. S7A). Particularly, a significant increase in the levels of representative metabolites of TCA cycles, citrate, fumarate and malate, was observed in the cytoplasm during AKI; however, maintaining Slc25a21 expression increased the metabolites ratio of mitochondria to cytoplasm (Fig. 6F). The regulation of Slc25a21 on representative enzymes involved in TCA cycle was confirmed in a separate in vivo AKI kidney model following high-pressure tail-vein injection of Slc25a21 plasmids, and in vitro using acutely injured TKPTS cells (Fig. S7B–D).

Here, we first demonstrated the localization of Slc25a21 in renal tubular mitochondria. We then showed a reduction in Slc25a21 expression in AKI-affected kidneys in both humans and mice, which results in compromised mitochondrial transport of 2-oxoadipate. The resultant cytoplasmic accumulation of 2-oxoadipate and relevant metabolic changes will directly or indirectly lead to the disturbance of mitochondrial homeostasis, mainly presenting decreased respiratory ATP generation, as well as compromised mitochondrial biogenesis, integrity, and an increase in ROS. Consequently, these alterations trigger tubular apoptosis, necroptosis as well as inflammatory responses, and ultimately, kidney dysfunction of AKI. Importantly, maintaining Slc25a21 expression preserves mitochondrial homeostasis and metabolic balance in tubular cells, which protects against the progression of AKI (Fig. 6G).

## Discussion

In this study, we report the association of Slc25a21 in renal tubular cells with the progression of cisplatin-induced AKI. Cisplatin suppresses Slc25a21 expression in tubular cells, leading to a disturbance in mitochondrial homeostasis and subsequent cell apoptosis, necroptosis as well as inflammation, contributing to AKI progression. Mechanistically, the reduction of Slc25a21 hampers the mitochondrial transport of 2-oxoadipate, which results in metabolic disturbance and mitochondrial energy alterations. We propose that maintaining Slc25a21 could be a new approach for metabolic intervention, aiming to rescue mitochondrial homeostasis and protect against tubular injury, and then prevent the progression of AKI.

Mitochondrial homeostasis refers to the balance and regulation of mitochondrial number, function, and quality within cells [35]. The kidney is one of the most metabolically active organs in the human body and requires substantial mitochondrial homeostasis to perform its proper functions [13]. Several kidney diseases are reported to be associated with disruptions in mitochondrial homeostasis [36, 37], with mitochondrial dysfunction being a major hallmark that affects the production of adenosine triphosphate (ATP) and leads to renal dysfunction [38]. Specifically, the proximal tubules are highly sensitive to energy fluctuations, as the first exhibiting signs of injury compared to other renal cell types [39]. The disruption of mitochondrial homeostasis is also a critical component in the pathogenesis of AKI [32, 40, 41], presenting damaged OXPHOS complex and mitochondrial respiration, impaired mitochondrial biogenesis and integrity, a deficit in cellular energy production and leading to tubular cell apoptosis and necroptosis [42–44]. Therapeutically, the modulation of homeostatic mechanisms was reported to protect against mitochondrial dysfunction in AKI [45–50]. Formoterol, as a potent, highly specific, and long-acting β2-adrenergic agonist, is shown to induce renal mitochondrial biogenesis. After injury has occurred, Formoterol works by restoring mitochondrial function and accelerating the recovery of renal function [51–53]. Mitochondria-targeted peptide (MTP) was designed to target the inner mitochondrial membrane, and reduce attenuated acute mitochondrial dysfunction and cell apoptosis in the kidneys [54]. Here, we provide proof of principle that maintaining Slc25a21 can be considered as a potential therapeutic approach to ameliorate AKI by restoring mitochondrial homeostasis. This approach counteracts cisplatin-induced mitochondrial dysfunction, improves cell respiration and ATP synthesis in tubular cells, and supports mitochondrial biogenesis and structural integrity.

Mitochondrial homeostasis is widely recognized as involving the metabolic cascades of energy substrates, ensuring the availability of sufficient energy (ATP) [55–57]. Aerobic respiration is the primary method of ATP production in tubular cells, starting with the conversion of glucose into pyruvate through glycolysis. Pyruvate is then transformed into acetyl-CoA, a crucial substrate for the TCA cycle, which facilitates ATP synthesis [58]. In addition to glucose, fatty acids are also major substrates for energy metabolism. The process of breaking down fatty acids via β-oxidation maximizes the production of acetyl-CoA through further oxidation [59]. The chemical reaction that initiates each “turn” of the TCA cycle is the condensation of the metabolite oxaloacetate (OAA) with acetyl-CoA to generate citrate. Subsequent reactions oxidize citrate to produce ATP molecules, depending on the specific enzymes involved at each step [33].

Acetate is a key metabolite for normal kidney development. Reductions in acetyl-CoA metabolism hinder normal kidney development, whereas supplementation stimulates it [60]. AKI induces progressive pyruvate depletion in the kidneys, while exogenous pyruvate administration leads to modest ATP increases and offer protection against kidney injury [61]. In addition, modulating the pyruvate carriers and decreasing mitochondrial pyruvate uptake is a central adaptive response following AKI, which enhances alternative metabolic pathways such as the pentose phosphate pathway and attenuates AKI severity [62]. During AKI, the intermediate metabolites and key enzymes related to citrate metabolism in the TCA cycle are consequently impacted, coupled with oxidative phosphorylation-driven ATP production. Slc25a21 is also an energy metabolic carrier. It transports 2-oxoadipate produced by the catabolism of cytosolic lysine (as well as tryptophan and hydroxylysine) into the mitochondrial matrix, where 2-oxoadipate is decarboxylated and further metabolized to provide a source of acetyl-CoA [23, 63]. Similar to other pathways modulating acetyl-CoA, sustaining Slc25a21 further enhanced the TCA cycle, presenting increased the expression of key enzymes, such as Citrate synthase (Cs) and Oxoglutarate dehydrogenase (Ogdh), and increased levels of citrate and malate in the mitochondria. Although the detailed functional and regulatory interplay between cytosolic lysine and the metabolism of glucose and fatty acids in coordinating energy supply is not fully understood [64], key metabolites such as acetyl-CoA, pyruvate, and 2-oxoadipate have proven to be crucial in maintaining mitochondrial homeostasis.

Furthermore, the Slc25a21-mediated mitochondrial transport of 2-oxoadipate is linked to tryptophan metabolic flux, as 2-oxoadipate is product of tryptophan metabolism. The maintenance of Slc25a21 leads to a strong decrease in the levels of the several metabolites produced by tryptophan metabolism, including Kynurenic acid, 3-Hydroxykynurenine, and Picolinic acid. However, dysregulation of tryptophan metabolism and elevated levels of these intermediates, have been observed in plasma and kidney tissues during AKI [65–71]. These metabolites have also been implicated in the inflammatory response in kidneys and further aggravated kidney dysfunction [72–76]. In addition, overproduction of ROS, the by-products of abnormal OXPHOS, is reported contribute to renal inflammation [77, 78]. Maintaining Slc25a21 restores mitochondrial homeostasis, and simultaneously reduces excessive ROS production. This reduction is beneficial for alleviating inflammatory responses in the injured kidneys. Thus, our data suggest a role of Slc25a21 in modulating inflammation during cisplatin-induced AKI.

Some limitations of our study should be highlighted. With respect to tubular cells as the main target of cisplatin-induced kidney injury and Slc25a21 being predominantly enriched in proximal tubular cells, in our study we have focused on renal tubular cells. However, our model of AAV9 system used multi-point in situ injection in the renal cortex to maintain the expression of Slc25a21, and non-specific promotor may lead to expression of exogenous Slc25a21 in multiple renal cells. Although the exogenous Slc25a21 following AAV9 application was mainly expressed in the PT cells (Figure S4A), we could not exclude the role of Slc25a21 in other cells which also contribute to AKI progression. Moreover, the mechanism by which cisplatin modulates Slc25a21 expression in renal tubular cells is not explored in this study. Liu et al. [79]. reported that lncRNA SLC25A21-AS1 regulates the stability of SLC25A21 mRNA in the cytoplasm. Additionally, the transcriptional regulation of another Slc25 family member, SLC25A13, was shown to involve USF1 as a main factor and FOXA2 as an activator in liver cells [80]. Understanding the genetic and epigenetic regulation of Slc25a21 expression in kidneys might provide more direct evidence of Slc25a21 as a target for cisplatin-induced AKI.

In conclusion, our study suggests novel therapeutic strategies for AKI by maintaining Slc25a21 expression to restore mitochondrial metabolic homeostasis. This strategy might effectively counteract tubular apoptosis, necroptosis and decrease inflammatory response, thereby mitigating AKI progression. Importantly, Slc25a21 ameliorates metabolic energy deficits in AKI by mediating the mitochondrial transport of 2-oxoadipate, supporting the concept that modulation of fuel metabolites is crucial for maintaining mitochondrial homeostasis in healthy kidney. Thus, the findings presented in our study provide new insights into mitochondrial metabolism in AKI, offering a promising perspective for interventions involving fuel metabolite supplementation and transportation.

## Supplementary information

Supplemental Material Table S1

Supplemental Material Table S2

Supplemental Material Table S3

Supplemental Material-supplementary information

Supplemental Material Original western blots

## Data Availability

The original data will be provided by the corresponding author upon reasonable request.
