# Supplementary material for: Slc25a21 in cisplatin-induced acute kidney injury: a new target for renal tubular epithelial protection by regulating mitochondrial metabolic homeostasis
Source: Cell Death Dis. 2024 Dec 18;15(12):891. doi: 10.1038/s41419-024-07231-2 (PMC11655545; doi:10.1038/s41419-024-07231-2)
Supplement: Supplementary file 4 — Supplemental Material-supplementary information [file 41419_2024_7231_MOESM4_ESM.docx]

**SUPPLEMENTARY INFORMATION**

**Slc25a21 in cisplatin-induced acute kidney injury: a new target for renal tubular epithelial protection by regulating mitochondrial metabolic homeostasis**

Xin Su^1,2,3, *^, Mi Bai^1,2,3^, Yaqiong Shang^1,2,3^, Yang Du^1,2,3^, Shuang Xu^1,2,3^, Xiuli Lin^1,2,3^, Yunzhi Xiao^4^, Yue Zhang^1,2,3^, Huimei Chen^4, *^, Aihua Zhang^1,2,3, *^

^1^Department of Nephrology, Children’s Hospital of Nanjing Medical University, Guangzhou Road 72, Nanjing 210008, China.

^2^Nanjing Key Laboratory of Pediatrics, Children’s Hospital of Nanjing Medical University, Nanjing 210008, China.

^3^Jiangsu Key Laboratory of Pediatrics, Nanjing Medical University, Nanjing 210029, China.

^4^Centre for Computational Biology and Programme in Cardiovascular and Metabolic Disorders, Duke-NUS Medical School, 8 College Road, 169857 Singapore.

^*^Correspondence to: zhaihua@njmu.edu.cn (A.Z), huimei.chen@duke-nus.edu.sg (H.C)，su_xin1201@163.com (X.S)


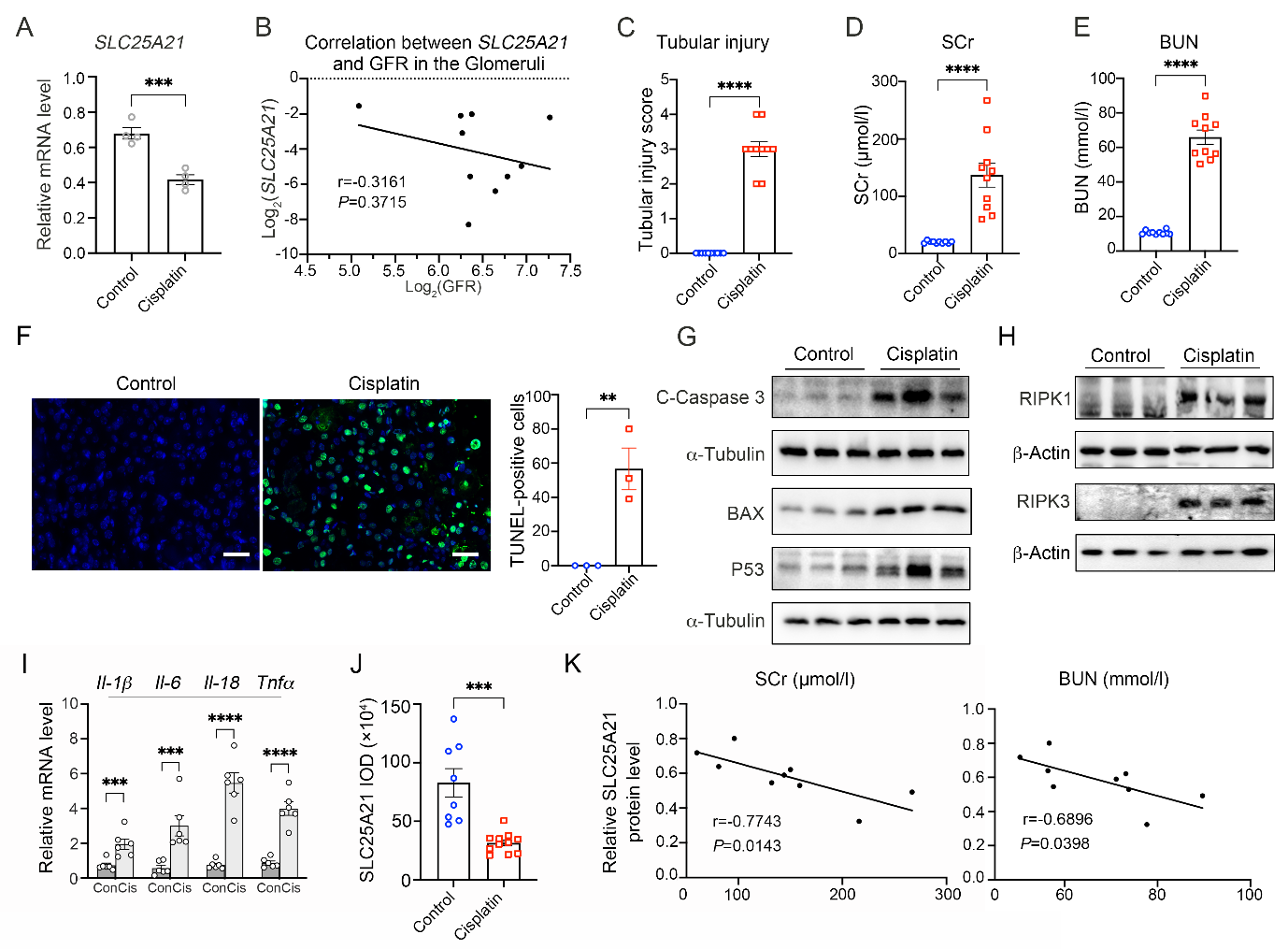


**Figure S1. Decreased renal expression of SLC25A21 is associated with AKI and cisplatin-induced kidney damage. Related to Figure 1.**

(A) The mRNA levels of *SLC25A21* from publicly available dataset of human iPSC-derived kidney organoids treated with/without cisplatin (GSE145085) (1).

(B) Pearson correlation between glomerular *SLC25A21* expression and glomerular filtration rate (GFR) in a healthy donor cohort (n=10, GSE30122) (2).

(C) The tubular injury score was analyzed based on Periodic Acid-Schiff (PAS) staining in cisplatin-induced AKI and control mice. n=10, each group, and values are reported as mean ± SEM.

(D) Concentrations of Serum creatinine (SCr) and (E) Blood Urea Nitrogen (BUN) determined in cisplatin-induced AKI and control mice. n=10, each group, and values are reported as mean ± SEM.

(F) Representative immune images for TUNEL expression in cisplatin-induced AKI and control kidneys (*left*), with quantification of TUNEL-positive cells per field (*right*). 200 × field images were recorded to calculate the mean for each mouse kidney, represented with one dot. n=3, each group. Scale bars, 50 µm.

(G) Representative western blotting for C-Caspase 3, Bax and P53 protein expression levels in cisplatin-induced AKI and control mice. C-Caspase 3: Cleaved-Caspase 3.

(H) Representative western blotting for RIPK1 and RIPK3 protein expression levels in cisplatin-induced AKI and control mice.

(I) Expression of inflammatory markers (*Il-1β, Il-6*, *Il-18, and Tnfα*), determined by RT-qPCR, in the kidneys from cisplatin-induced AKI and control mice. n=6, each group, and values are reported as mean ± SEM.

(J) The semi-quantitative IOD analysis of SLC25A21 expression in cisplatin-induced AKI and control mice. IOD, integral optical density.

(K) Pearson correlation analysis between Slc25a21 protein expression relative to control in the kidney and Serum creatinine (SCr, *left*), as well as Blood Urea Nitrogen (BUN, *right*) in cisplatin-induced AKI mice (n=9).

In each case, data were presented as means ±SEM and statistical significance is assessed by the unpaired Student’s t-test. ** indicates *P* < 0.01; **** indicates *P* < 0.0001 compared between two groups.


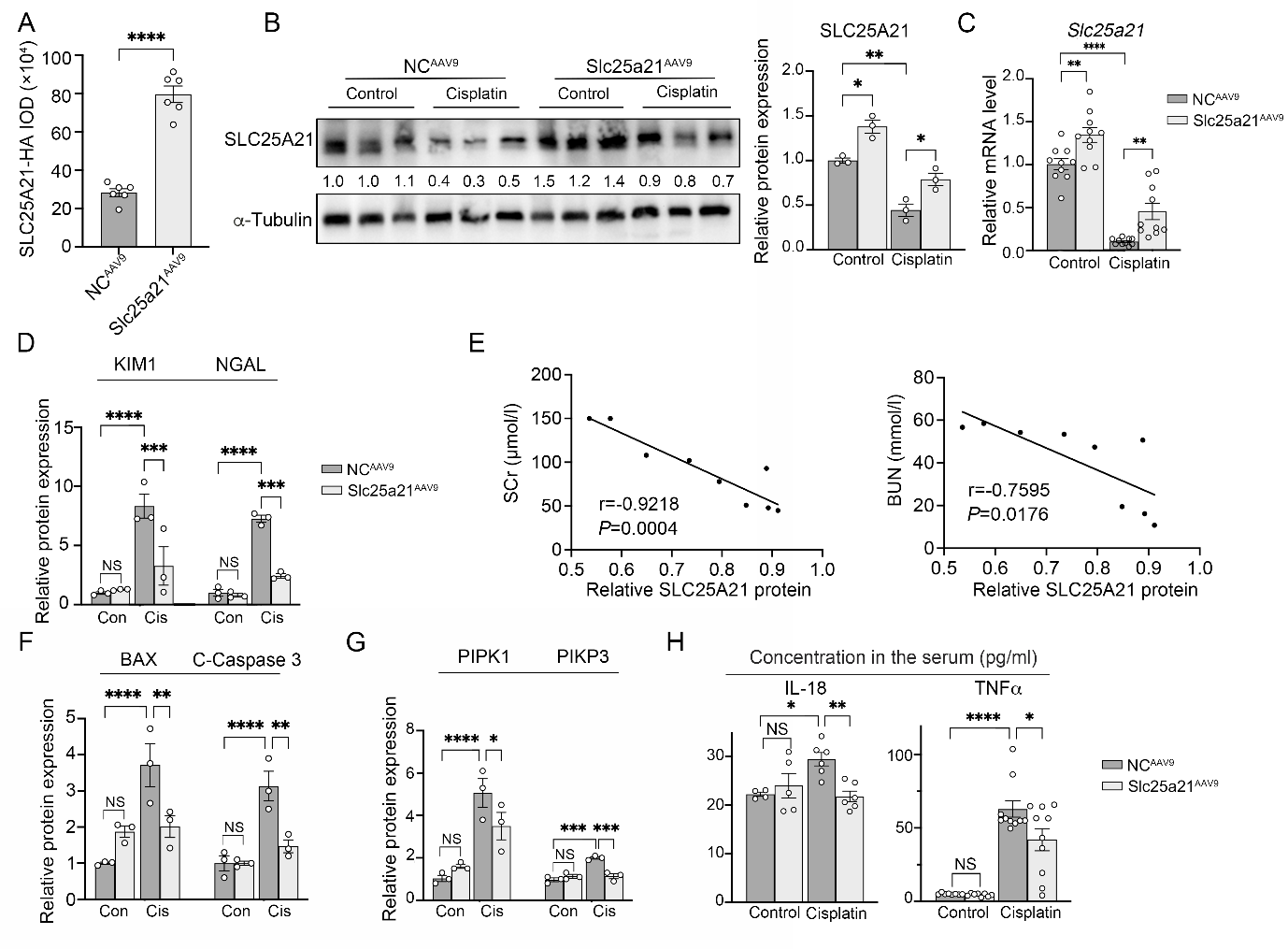


**Figure S2**. **AAV9-sustaining Slc25a21 renal expression attenuated cisplatin-induced AKI. Related to Figure 2.**

(A) The semi-quantitative IOD analysis of Slc25a21-targeted HA expression in Slc25a21^AAV9^ and NC^AAV9^ mice. IOD, integral optical density.

(B) The expression of Slc25a21 in the kidneys from both Slc25a21^AAV9^ and NC^AAV9^ mice following cisplatin or saline injection. *left*: Representative western blotting for Slc25a21 protein level; *right*: The densitometric analysis for Slc25a21 protein expression.

(C) *Slc25a21* mRNA expression changes in the kidneys from both Slc25a21^AAV9^ and NC^AAV9^ mice following cisplatin or saline injection were determined by RT-qPCR. n=10, each group, and values are reported as mean ± SEM.

(D) The densitometric analysis for KIM1 and NGAL protein expression in the kidneys from both Slc25a21^AAV9^ and NC^AAV9^ mice following cisplatin or saline injection. n=3, each group, and values are reported as mean ± SEM.

(E) Pearson correlation analysis between increased relative Slc25a21 protein expression in the kidney and Serum creatinine (SCr, *left*), as well as Blood Urea Nitrogen (BUN, *right*) in Slc25a21^AAV9^ AKI mice (n=9).

(F) The densitometric analysis for BAX and Cleaved-Caspase 3 (C-Caspase 3) protein expression in the kidneys from both Slc25a21^AAV9^ and NC^AAV9^ mice following cisplatin or saline injection. n=3, each group, and values are reported as mean ± SEM.

(G) The densitometric analysis for RIPK1 and RIPK3 protein expression in the kidneys from both Slc25a21^AAV9^ and NC^AAV9^ mice following cisplatin or saline injection. n=3, each group, and values are reported as mean ± SEM.

(H) Serum concentrations of IL-18 and TNFα from Slc25a21^AAV9^ and NC^AAV9^ mice after cisplatin or saline injection, determined by ELISA assay. n=4-10, each group, and values are reported as mean ± SEM.

In each case, data were presented as means ±SEM and statistical significance is assessed by One-way ANOVA analysis of variance of Tukey's multiple comparisons test or the unpaired Student’s t-test. * indicates *P* < 0.05; ** indicates *P* < 0.01; *** indicates *P* < 0.001; **** indicates *P* < 0.0001; NS indicates not significant.

**Slc25a21 preservation protected against cisplatin-induced AKI though high-pressure tail-vein injection of plasmids**

To further elucidate the role of Slc25a21 in cisplatin-induced AKI, we introduced Slc25a21 plasmids into mice through high-pressure tail-vein injection, designated as Slc25a21^DNA^ (Fig. S3A). As depicted in Fig. S3B, both the protein and mRNA expression of Slc25a21 in the kidneys significantly increased following the administration of Slc25a21 plasmids, indicating the efficiency of this gene delivery approach in achieving Slc25a21 overexpression in the kidneys. Subsequent Slc25a21 plasmid-delivery, these mice received 25 mg/kg cisplatin injection. Consistent with the results obtained by the kidneys *in situ* injection of AAV9-Slc25a21, introducing Slc25a21 plasmids also markedly attenuated renal injury and improved renal function in AKI mice compared to those injected with negative control plasmids (NC^DNA^), as evidenced by diminished tubular dilatation based on PAS staining results (Fig. S3C); decreased NGAL protein expression according to western blotting results (Fig. S3D); the reduced ratio of kidney to body weight and lower concentrations of SCr and BUN in the mouse serum (Fig. S3E). Moreover, fewer TUNEL-positive cells were observed in Slc25a21^DNA^ mice following cisplatin administration (Fig. S3F). The up-regulated levels of cell apoptosis markers (C-Caspase 3, BAX and P53) and cell necroptosis markers (MLKL, RIPK1 and RIPK3) were obviously reduced upon Slc25a21 preservation (Fig. S3G-H). Additionally, the mRNA expression of inflammatory markers (Interleukin-1β (*Il-1β*), *Il-6*, *Il-18* and *Tnfα*), along with the circulating concentrations of IL-18 and TNFα in the serum, were decreased in AKI kidneys upon Slc25a21 preservation (Fig. S3I-J). Collectively, these findings underscore the significance of Slc25a21 preservation in mitigating cisplatin-induced acute kidney injury.

**
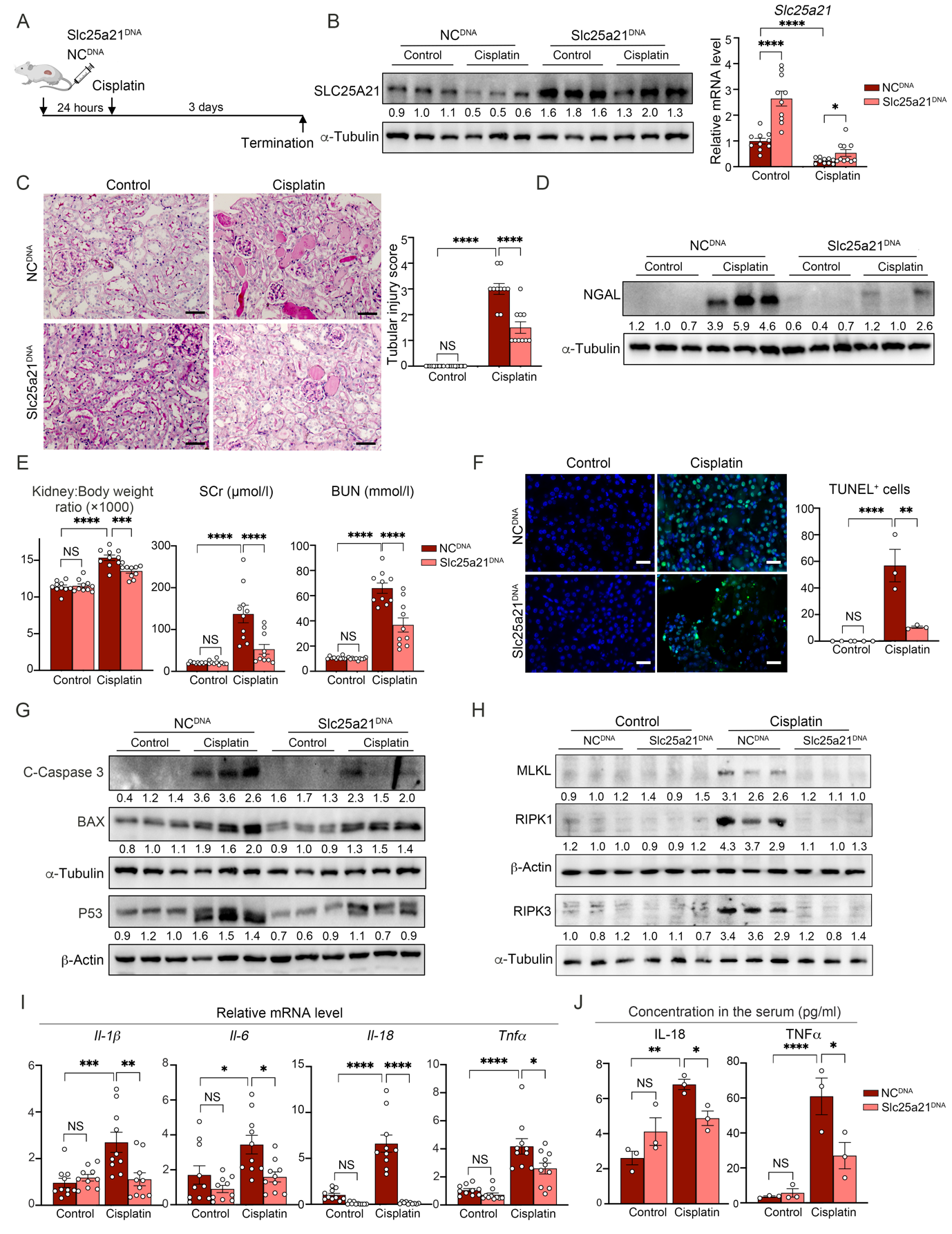
**

**Figure S3**. **Slc25a21 preservation protected against cisplatin-induced AKI though high-pressure tail-vein injection of plasmids. Related to Figure 2.**

(A) Experimental schematic of mice receiving Slc25a21^DNA^ or NC^DNA^ plasmids introduction into the mouse kidneys via high-pressure tail-vein injection within 7 s, followed by cisplatin treatment (25 mg/kg) for three days to induce AKI.

(B) The expression of Slc25a21 in the kidneys from both Slc25a21^DNA^ and NC^DNA^ mice following cisplatin or saline addition. *left*: Representative western blotting for Slc25a21 protein level; *right:* mRNA expression changes were determined by RT-qPCR. n=10, each group, and values are reported as mean ± SEM.

(C) Representative Periodic Acid-Schiff (PAS) staining image in Slc25a21^DNA^ and NC^DNA^ mice following cisplatin or saline addition. *left*: Representative PAS staining for AKI lesions in the kidneys. Scale bars, 50 µm. *right*: semi-quantitative analysis of tubular injury score in the kidneys. n=10, each group.

(D) Representative western blotting for NGAL protein level in Slc25a21^DNA^ and NC^DNA^ mice after cisplatin or saline injection.

(E) The ratio of kidney to body weight and concentrations of SCr and BUN in Slc25a21^DNA^ and NC^DNA^ mice following cisplatin or saline administration. n=10, each group, and values are reported as mean ± SEM.

(F) Representative immune images for TUNEL expression in Slc25a21^DNA^ and NC^DNA^ mice after cisplatin or saline injection (*left*), with quantification of TUNEL-positive cells per field (*right*). 200 × field images were recorded to calculate the mean for each mouse kidney, represented with one dot. n=3, each group, and values are reported as mean ± SEM. Scale bars, 50 µm.

(G) Representative western blotting image for C-Caspase 3, BAX and P53 protein expression levels in Slc25a21^DNA^ and NC^DNA^ mice following cisplatin or saline administration. C-Caspase 3: Cleaved-Caspase 3.

(H) Representative western blotting image for MLKL, RIPK1 and RIPK3 protein expression levels in Slc25a21^DNA^ and NC^DNA^ mice following cisplatin or saline administration.

(I) Expression of inflammatory markers (*Il-1β*, *Il-6*, *Il-18* and *Tnfα*), determined by RT-qPCR, in the kidneys from Slc25a21^DNA^ and NC^DNA^ mice following cisplatin or saline administration. n=10, each group, and values are reported as mean ± SEM.

(J) Serum concentrations of IL-18 and TNFα from Slc25a21^DNA^ and NC^DNA^ mice after cisplatin or saline injection, determined by ELISA assay. n=3, each group, and values are reported as mean ± SEM.

In each case, data were presented as means ±SEM and statistical significance is assessed by One-way ANOVA analysis of variance of Tukey's multiple comparisons test. * indicates *P* < 0.05; ** indicates *P* < 0.01; *** indicates *P* < 0.001; **** indicates *P* < 0.0001; NS indicates not significant.

**
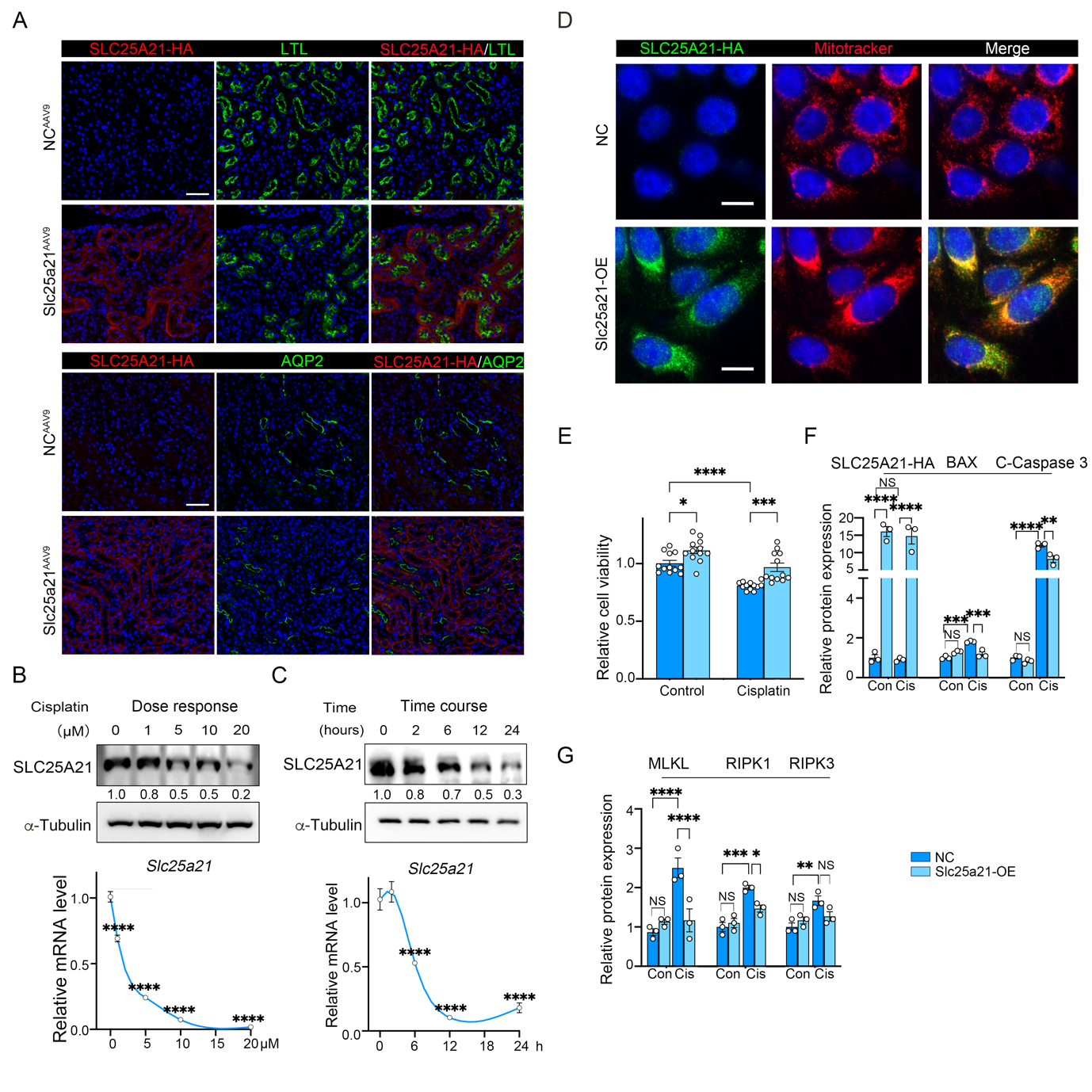
**

**Figure S4. Rescuing Slc25a21 ameliorates cisplatin-induced acute tubular injury *in vitro*. Related to Figure 3.**

(A) Representative immunofluorescence image depicting co-staining of Slc25a21-targeted HA with Lotus tetragonolobus lectin (LTL) and AQP2 in the Slc25a21^AAV9^ and NC^AAV9^ mice. Green presents LTL or AQP2; red presents SLC25A21-HA; blue presents DAPI. Scale bars, 50 µm.

(B) The expression of Slc25a21 *in vitro,* detected by TKPTS cells in a cisplatin-dose-dependent manner. *upper*: Representative western blotting for Slc25a21 protein level, determined by TKPTS cells exposed to 0, 1, 5, 10, 20 µM cisplatin for 24 h; *down*: *Slc25a21* mRNA expression changes were determined by RT-qPCR in TKPTS cells treated with a different gradient of cisplatin dose response for 24 h. n=6, each group, and values are reported as mean ± SEM. * indicates exposed-cisplatin TKPTS cells *vs* normal control cells.

(C) The expression of Slc25a21 *in vitro,* detected by TKPTS cells in a cisplatin-time-dependent manner. *upper*: Representative western blotting for Slc25a21 protein level, determined by TKPTS cells exposed to cisplatin (10 µM) for 0, 2, 6, 12, 24 h; *down*: *Slc25a21* mRNA expression changes were determined by RT-qPCR in TKPTS cells treated with cisplatin (10 µM) for a different time course. n=6, each group, and values are reported as mean ± SEM. * indicates exposed-cisplatin TKPTS cells *vs* normal control cells.

(D) Representative immunofluorescence image of co-staining of Slc25a21-targeted HA with Mitotracker revealed the mitochondrial localization of exogenous Slc25a21 in TKPTS cells. Green presents SLC25A21-HA; red presents Mitotracker; blue presents DAPI. Scale bars, 20 µm.

(E) The ratio of cell viability relative to control detected in Slc25a21-OE and NC cells after cisplatin (10 µM) treatment for 24 h. n=12, each group, and values are reported as mean ± SEM.

(F) The densitometric analysis for SLC25A21-HA, BAX and C-Caspase 3 protein expression in Slc25a21-OE and NC cells after cisplatin (10 µM) treatment for 24 h.

(G) The densitometric analysis for MLKL, RIPK1 and RIPK3 protein expression in Slc25a21-OE and NC cells after cisplatin (10 µM) treatment for 24 h.

In each case, data were presented as means ±SEM and statistical significance is assessed by One-way ANOVA analysis of variance of Tukey's multiple comparisons test or the unpaired Student’s t-test. * indicates *P* < 0.05; ** indicates *P* < 0.01; *** indicates *P* < 0.001; **** indicates *P* < 0.0001; NS indicates not significant.


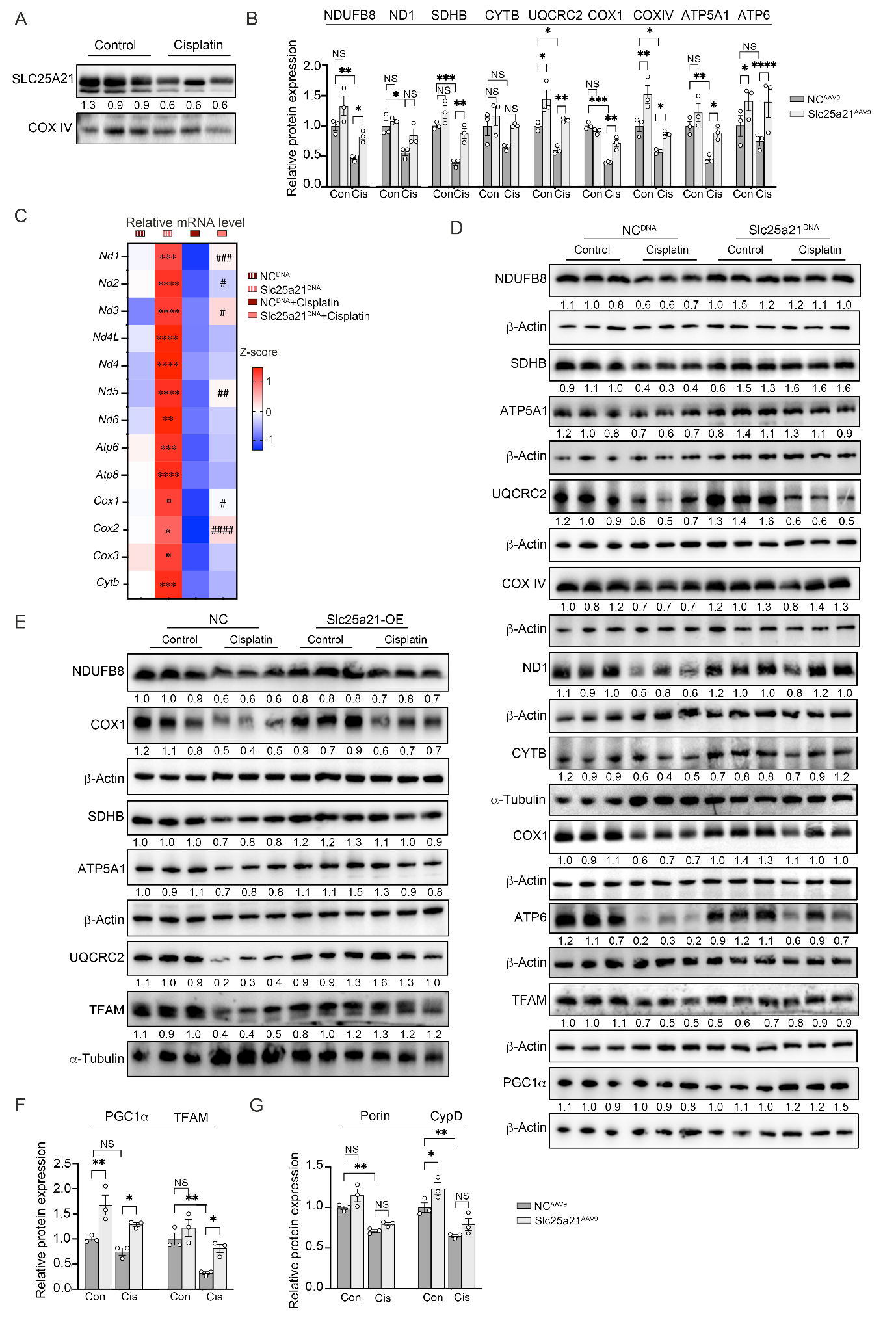


**Figure S5. Slc25a21 is important for preserving mitochondrial homeostasis during AKI. Related to Figure 4.**

(A) Representative western blotting for Slc25a21expression level in the mitochondria of cisplatin-induced AKI and control mice.

(B) The densitometric analysis for protein expression of enzymes involved in mitochondrial OXPHOS in the kidneys of Slc25a21^AAV9^ and NC^AAV9^ mice after cisplatin or saline addition.

(C) Heatmap showing mRNA expression of the 13 mitochondrially encoded genes in Slc25a21^DNA^ and NC^DNA^ mice after cisplatin or saline addition, determined by RT-qPCR (n=10). Data are presented as Z-score and statistical significance is assessed by One-way ANOVA analysis of variance of Tukey's multiple comparisons test. * indicates *P* < 0.05; ** indicates *P* < 0.01; *** indicates *P* < 0.001; **** indicates *P* < 0.0001; # indicates *P* < 0.05; ## indicates *P* < 0.01; ### indicates *P* < 0.001; #### indicates *P* < 0.0001. * indicates Slc25a21^DNA^ *vs* NC^DNA^, # indicates Slc25a21^DNA^+Cisplatin *vs* NC^DNA^+Cisplatin.

(D) Representative western blotting for protein expression levels of enzymes involved in mitochondrial OXPHOS and biogenesis in Slc25a21^DNA^ and NC^DNA^ mice after cisplatin or saline injection.

(E) Representative western blotting for protein expression levels of enzymes relevant to mitochondrial OXPHOS and biogenesis in Slc25a21-OE and normal control (NC) cells after cisplatin (10 µM) treatment for 24 h.

(F) The densitometric analysis for PGC1α and TFAM protein expression in the kidneys of Slc25a21^AAV9^ and NC^AAV9^ mice after cisplatin or saline addition.

(G) The densitometric analysis for Porin and CypD protein expression in the kidneys of Slc25a21^AAV9^ and NC^AAV9^ mice after cisplatin or saline addition.

In each case, data were presented as means ±SEM and statistical significance is assessed by One-way ANOVA analysis of variance of Tukey's multiple comparisons test. * indicates *P* < 0.05; ** indicates *P* < 0.01; *** indicates *P* < 0.001; **** indicates *P* < 0.0001; NS indicates not significant.

**
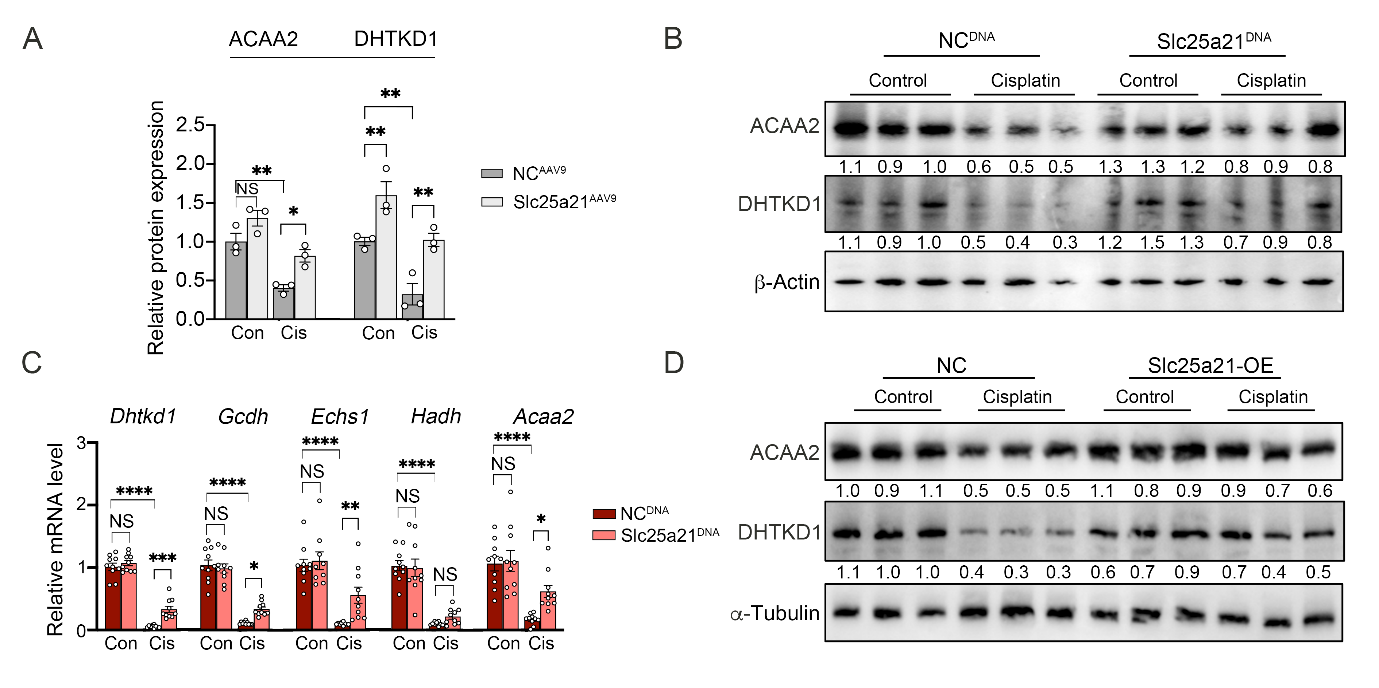
**

**Figure S6. Slc25a21 facilitates mitochondrial 2-oxoadipate transport and metabolism. Related to Figure 5.**

(A) The densitometric analysis for ACAA2 and DHTKD1 protein expression in the kidneys of Slc25a21^AAV9^ and NC^AAV9^ mice after cisplatin or saline addition.

(B) Representative western blotting for protein expression levels of key enzymes involved in 2-oxoadipate metabolism in Slc25a21^DNA^ and NC^DNA^ mice after cisplatin or saline injection.

(C) mRNA expression of enzymes associated with 2-oxoadipate metabolism in Slc25a21^DNA^ and NC^DNA^ mice after cisplatin or saline addition, determined by RT-qPCR (n=10). Data were presented as means ±SEM and statistical significance is assessed by One-way ANOVA analysis of variance of Tukey's multiple comparisons test. * indicates *P* < 0.05; ** indicates *P* < 0.01; *** indicates *P* < 0.001; NS indicates not significant.

(D) Representative western blotting for protein expression levels of enzymes associated with 2-oxoadipate metabolism in Slc25a21-OE and NC cells after cisplatin (10 µM) treatment for 24 h.

In each case, data were presented as means ±SEM and statistical significance is assessed by One-way ANOVA analysis of variance of Tukey's multiple comparisons test. * indicates *P* < 0.05; ** indicates *P* < 0.01; *** indicates *P* < 0.001; **** indicates *P* < 0.0001; NS indicates not significant.


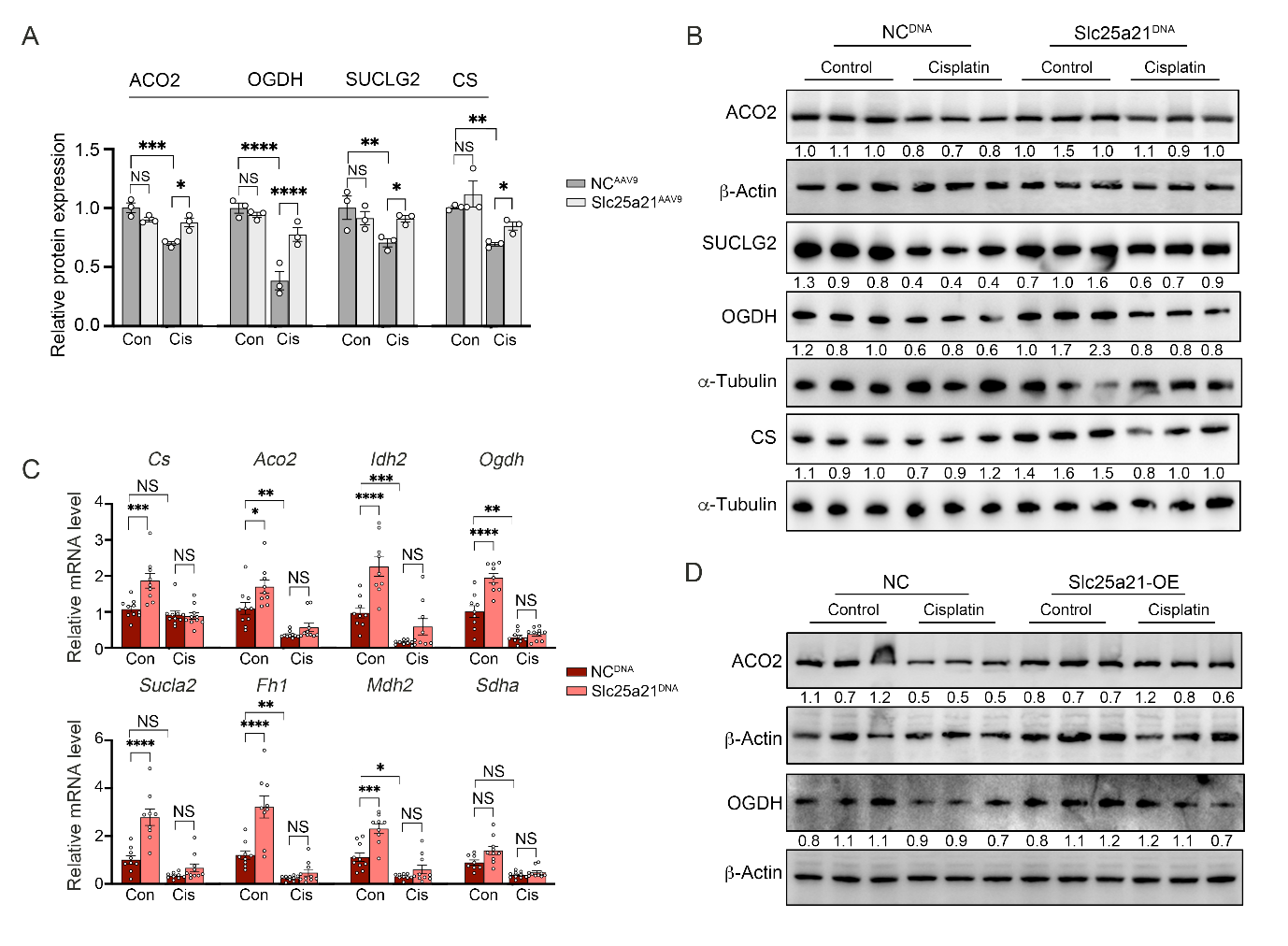


**Figure S7. TCA cycle enriched in the metabolism regulated by Slc25a21 in AKI. Related to Figure 6.**

(A) The densitometric analysis for ACO2, OGDH, SUCLG2 and CS protein expression in the kidneys of Slc25a21^AAV9^ and NC^AAV9^ mice after cisplatin or saline addition.

(B) Representative western blotting for protein expression levels of key enzymes associated with the TCA cycle in Slc25a21^DNA^ and NC^DNA^ mice after cisplatin or saline injection.

(C) mRNA expression of enzymes associated with the TCA cycle in Slc25a21^DNA^ and NC^DNA^ mice after cisplatin or saline addition, determined by RT-qPCR (n=8-10). Data were presented as means ±SEM and statistical significance is assessed by One-way ANOVA analysis of variance of Tukey's multiple comparisons test. * indicates *P* < 0.05; *** indicates *P* < 0.001; **** indicates *P* < 0.0001; NS indicates not significant.

(D) Representative western blotting for protein expression levels of enzymes associated with the TCA cycle in Slc25a21-OE and NC cells after cisplatin (10 µM) treatment for 24 h.

In each case, data were presented as means ±SEM and statistical significance is assessed by One-way ANOVA analysis of variance of Tukey's multiple comparisons test. * indicates *P* < 0.05; ** indicates *P* < 0.01; *** indicates *P* < 0.001; **** indicates *P* < 0.0001; NS indicates not significant.

REFERENCES

1. Digby JLM, Vanichapol T, Przepiorski A, Davidson AJ, Sander V. Evaluation of cisplatin-induced injury in human kidney organoids. Am J Physiol Renal Physiol. 2020;318(4):F971-F8.

2. Woroniecka KI, Park AS, Mohtat D, Thomas DB, Pullman JM, Susztak K. Transcriptome analysis of human diabetic kidney disease. Diabetes. 2011;60(9):2354-69.
