# Supplementary material for: Slc25a21 in cisplatin-induced acute kidney injury: a new target for renal tubular epithelial protection by regulating mitochondrial metabolic homeostasis
Source: Cell Death Dis. 2024 Dec 18;15(12):891. doi: 10.1038/s41419-024-07231-2 (PMC11655545; doi:10.1038/s41419-024-07231-2)

Figure 1E

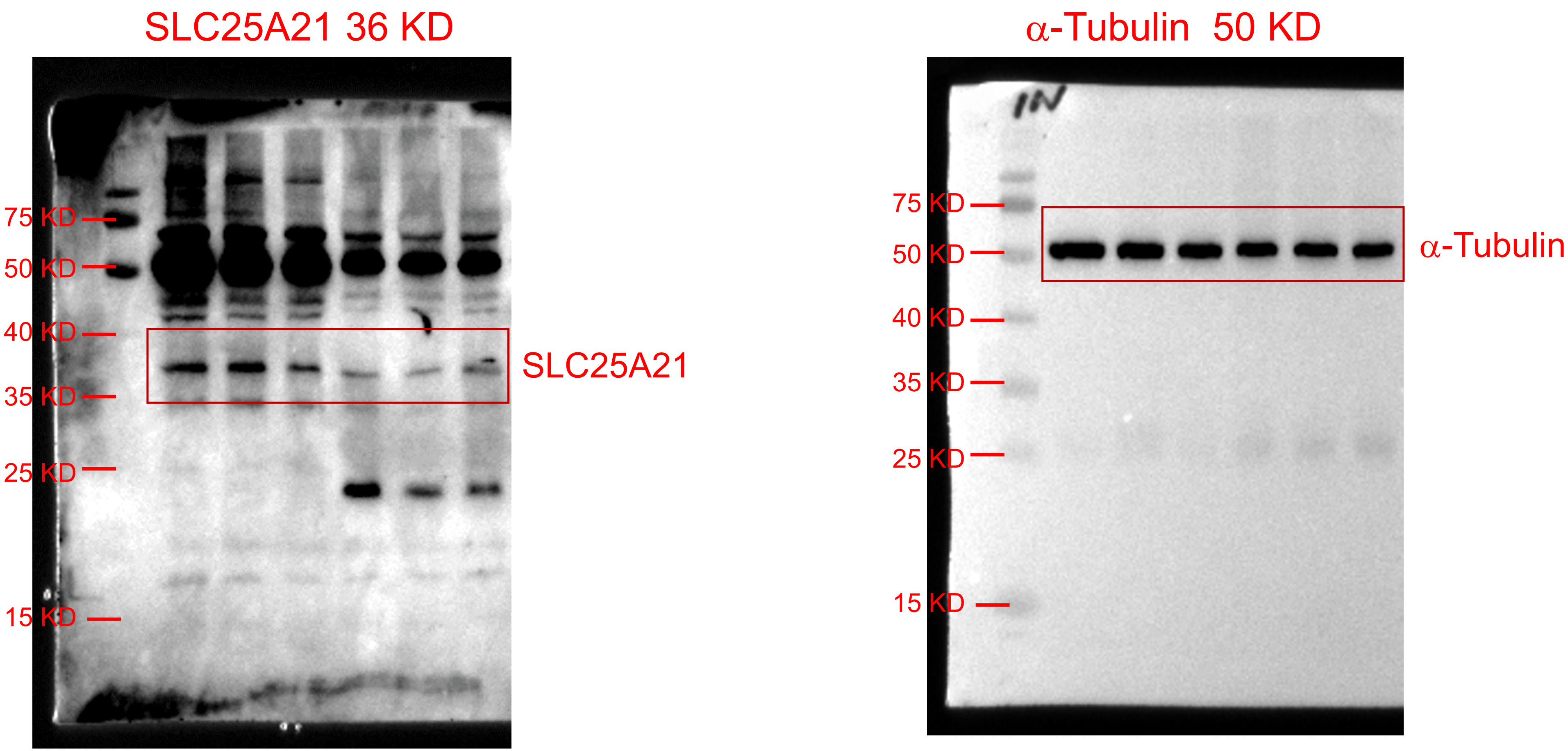
SLC25A21 36 KD
75 KD
50 KD
40 KD
35 KD
25 KD
15 KD
SLC25A21
α-Tubulin 50 KD
75 KD
50 KD
40 KD
35 KD
25 KD
15 KD
α-Tubulin

Figure 2C

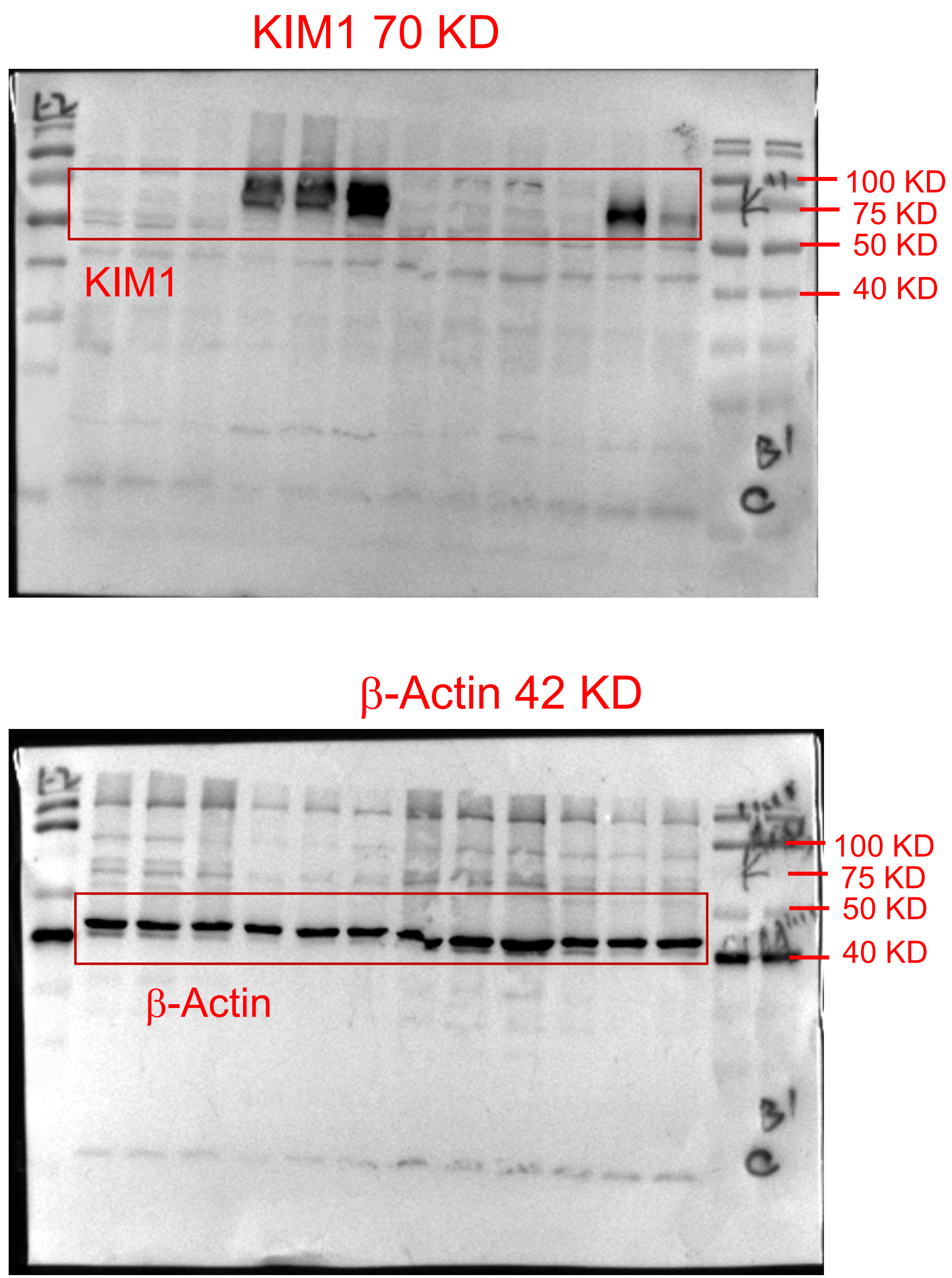

Figure 2C

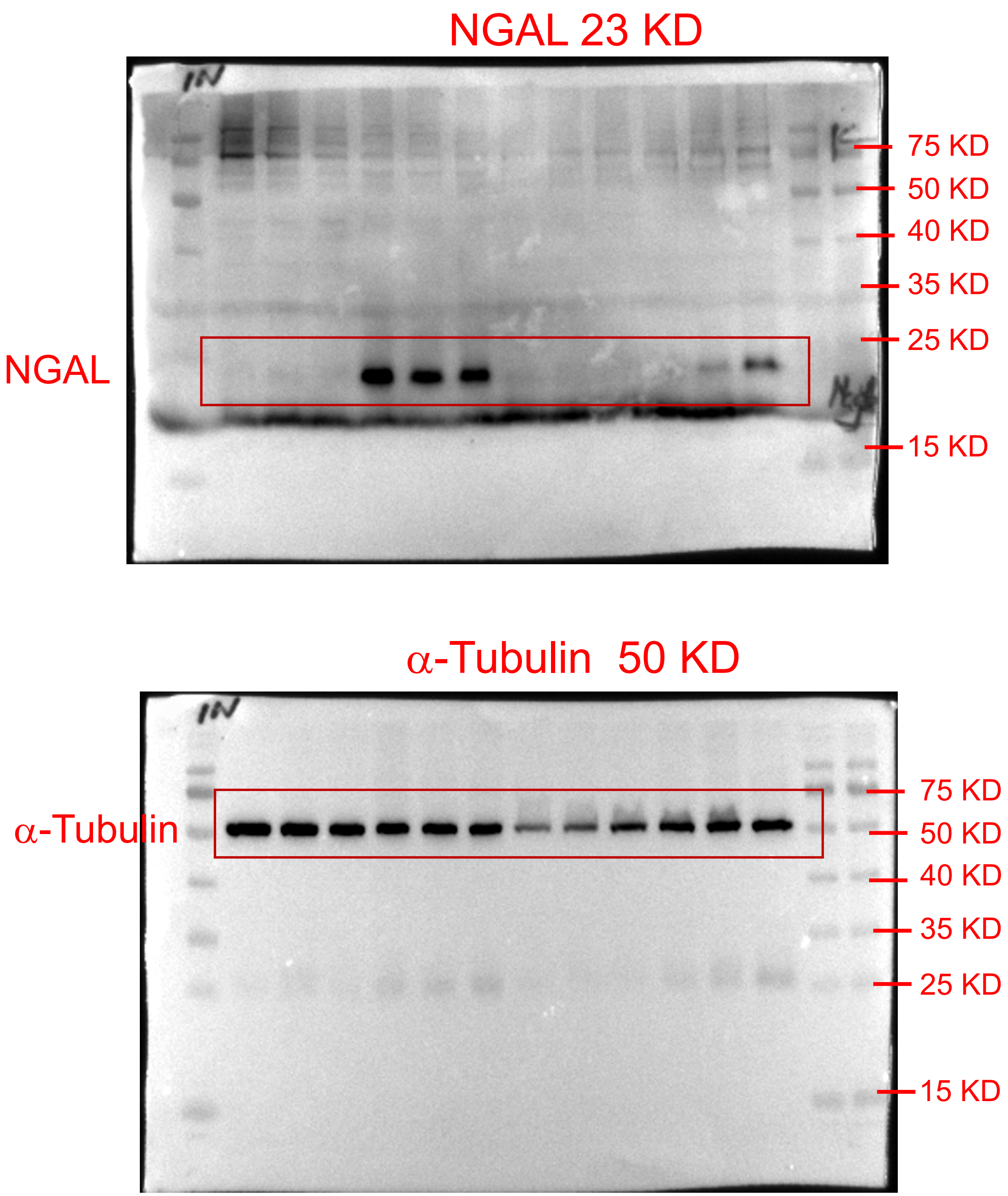

Figure 2F

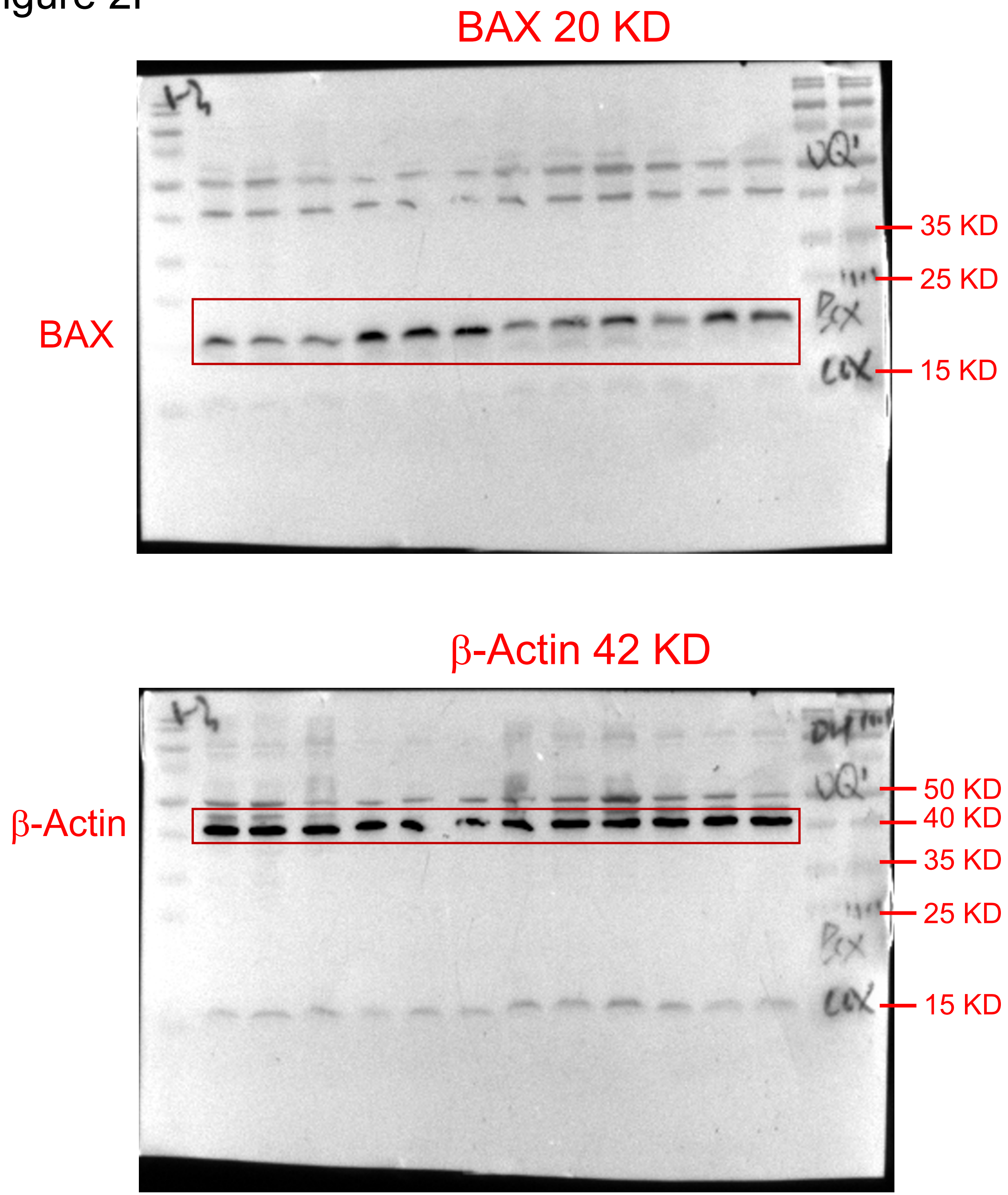

Figure 2F

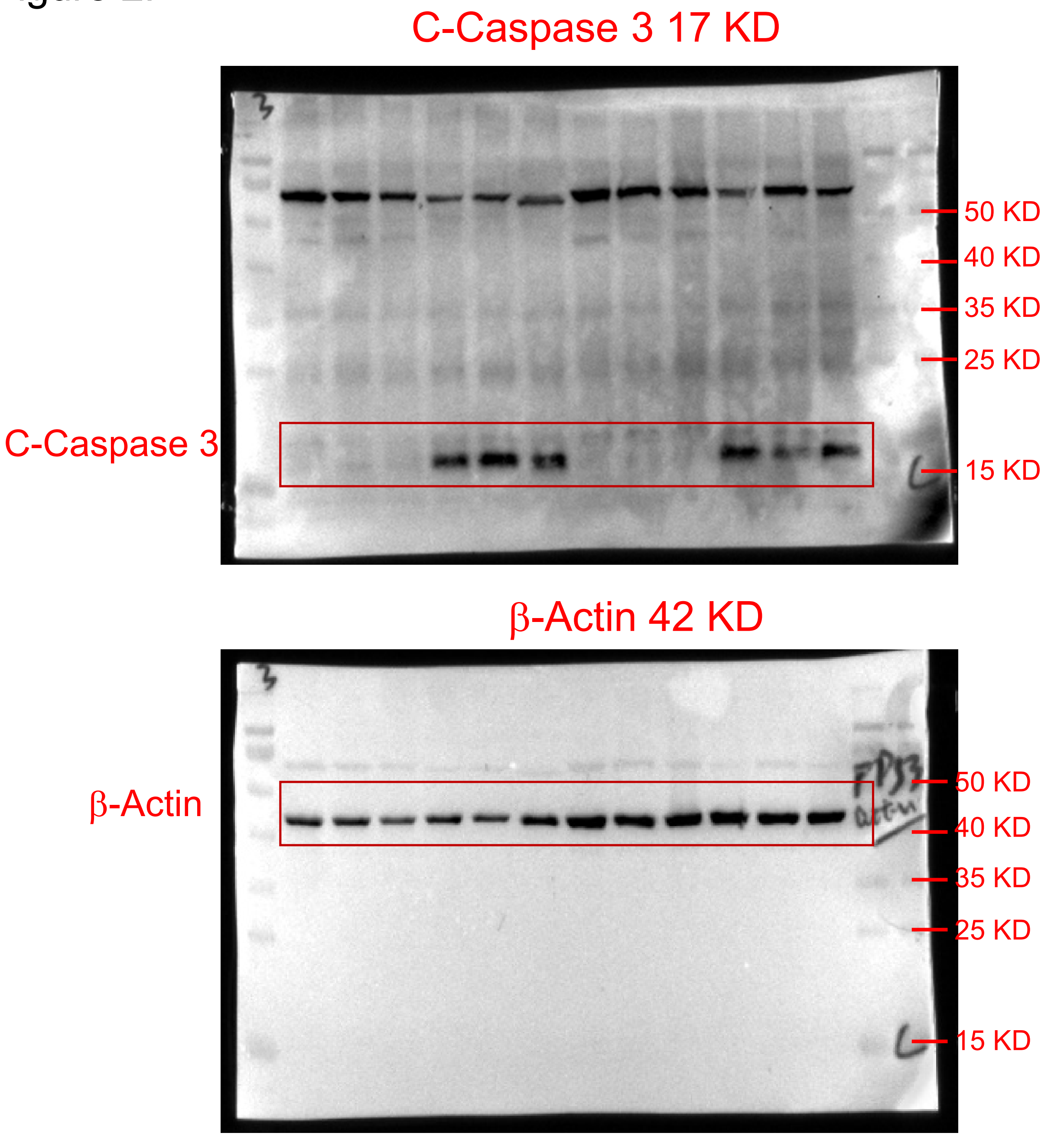

Figure 2G

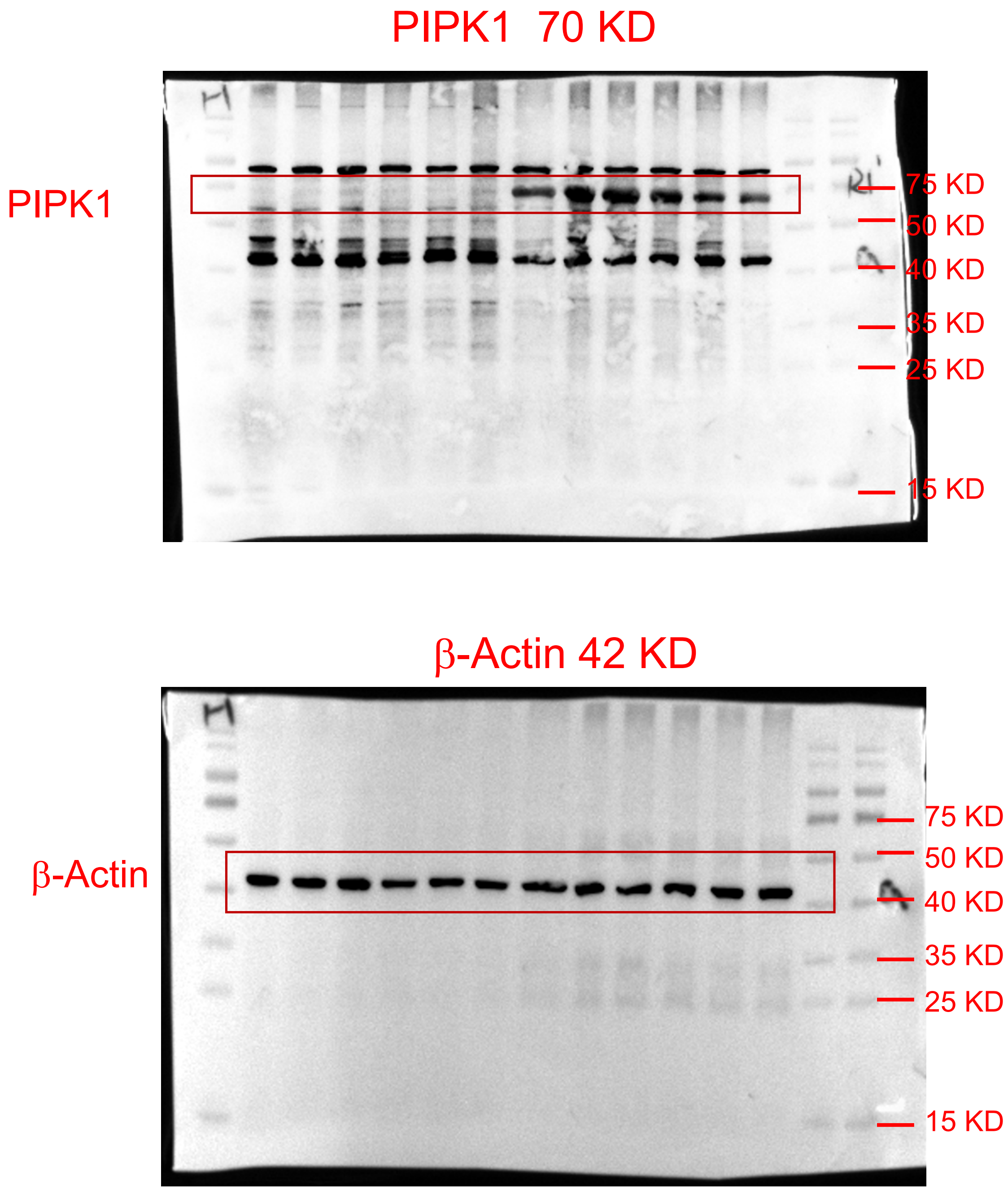

Figure 2G

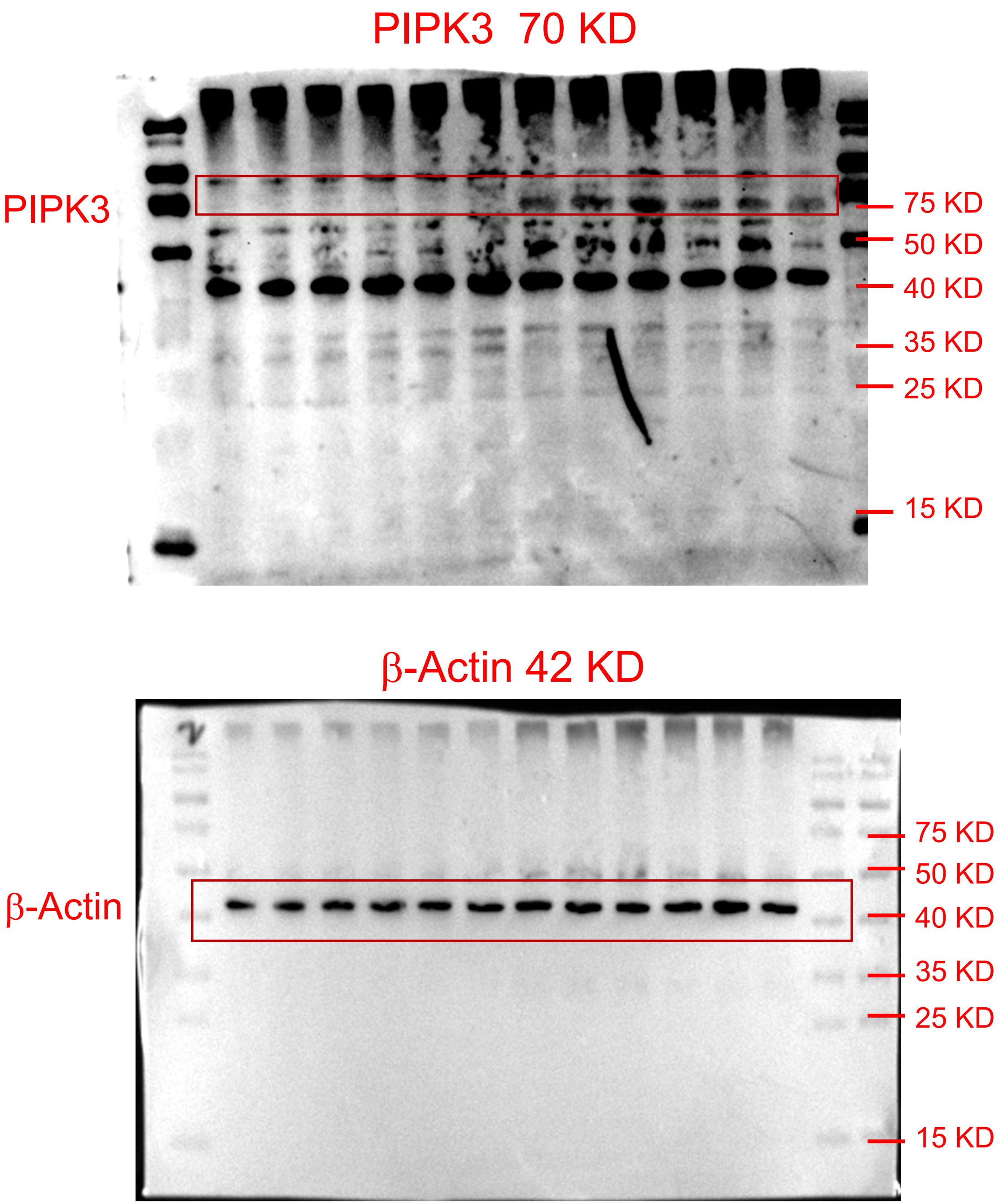

Figure 3D

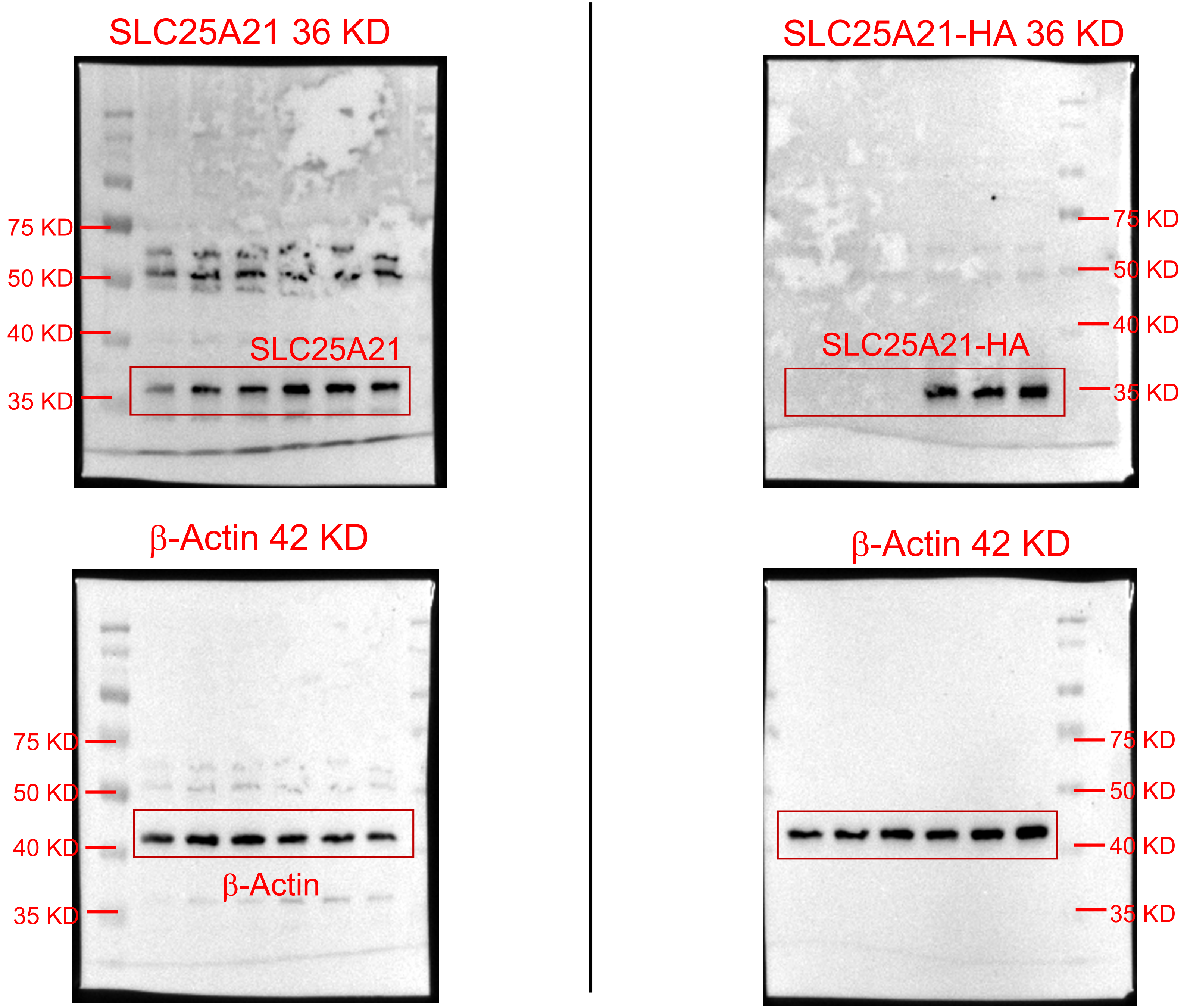
SLC25A21 36 KD
75 KD
50 KD
40 KD
35 KD
SLC25A21
SLC25A21-HA 36 KD
75 KD
50 KD
40 KD
35 KD
SLC25A21-HA
β-Actin 42 KD
75 KD
50 KD
40 KD
35 KD
β-Actin
β-Actin 42 KD
75 KD
50 KD
40 KD
35 KD

Figure 3F

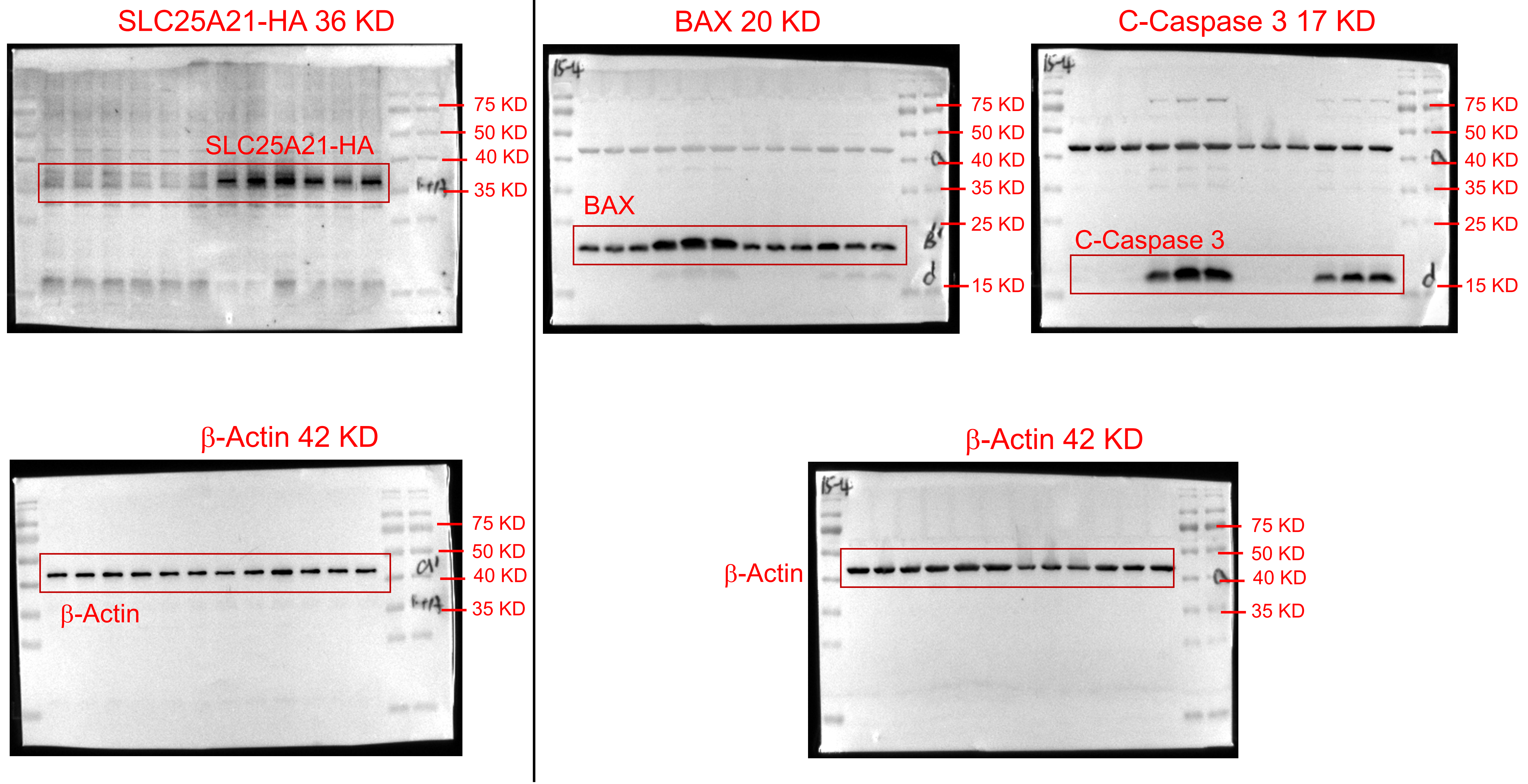
SLC25A21-HA 36 KD
SLC25A21-HA
75 KD
50 KD
40 KD
35 KD
BAX 20 KD
BAX
75 KD
50 KD
40 KD
35 KD
25 KD
15 KD
C-Caspase 3 17 KD
C-Caspase 3
75 KD
50 KD
40 KD
35 KD
25 KD
15 KD
β-Actin 42 KD
β-Actin
75 KD
50 KD
40 KD
35 KD
β-Actin 42 KD
β-Actin
75 KD
50 KD
40 KD
35 KD

Figure 3G

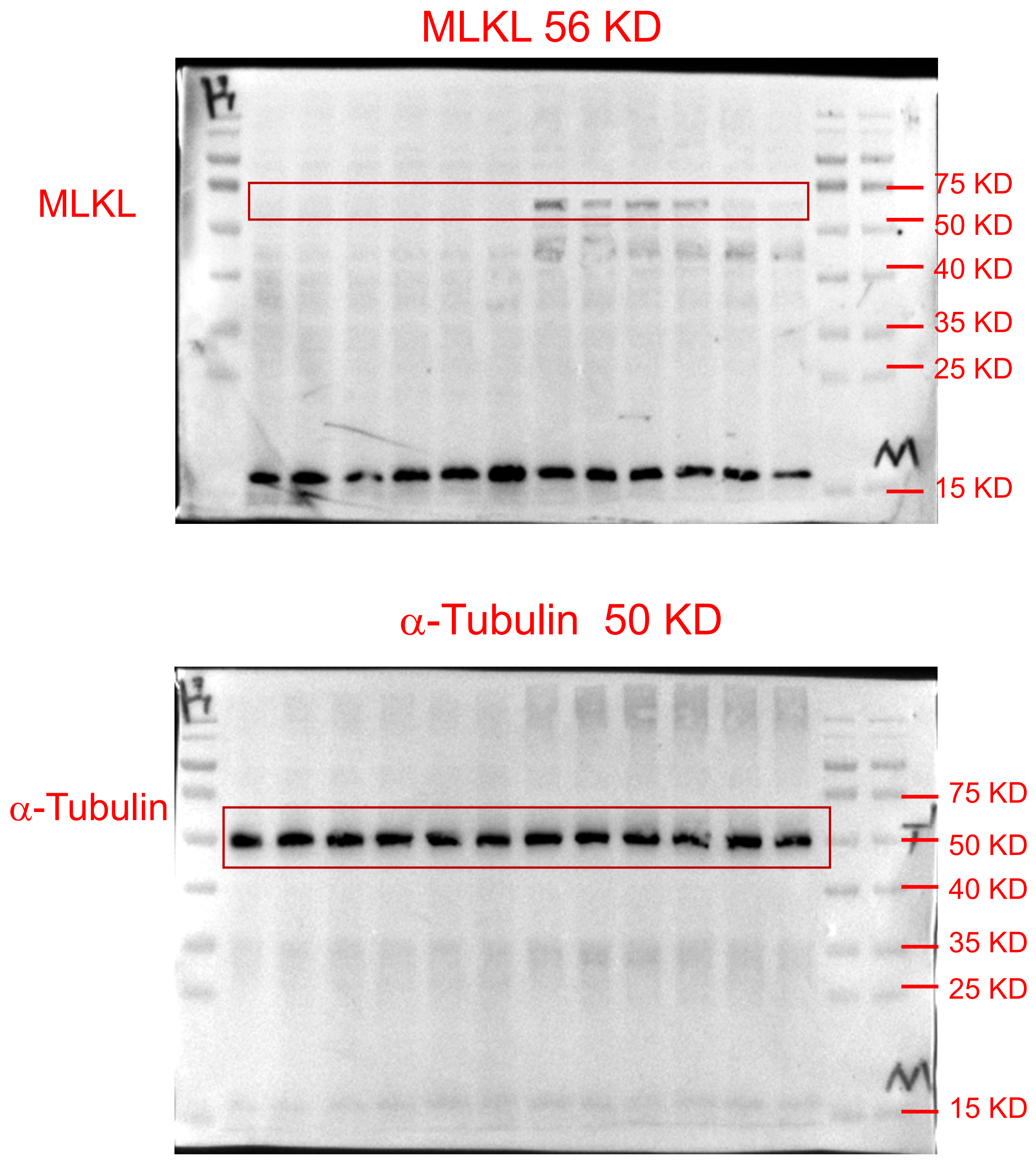

Figure 3G

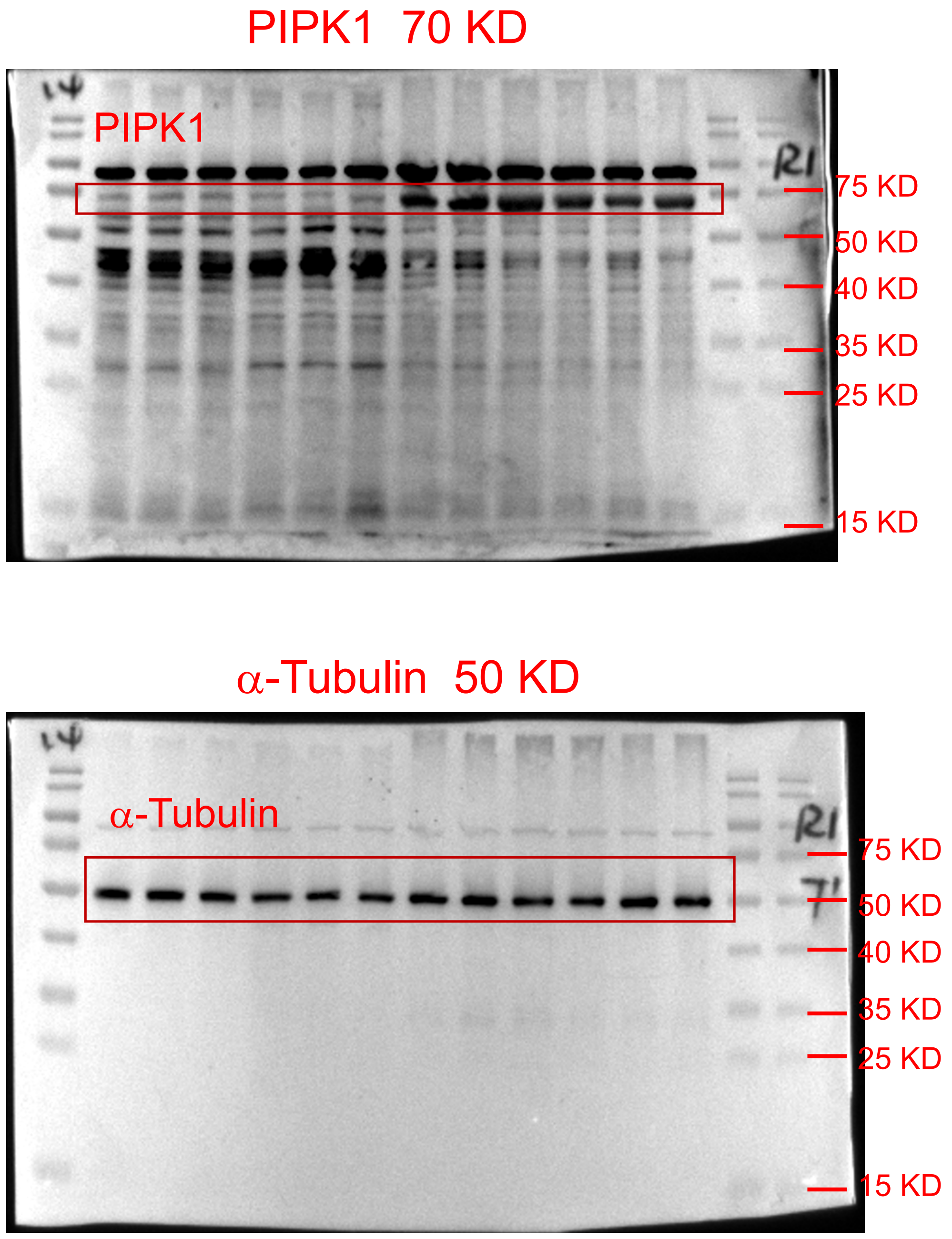

Figure 3G

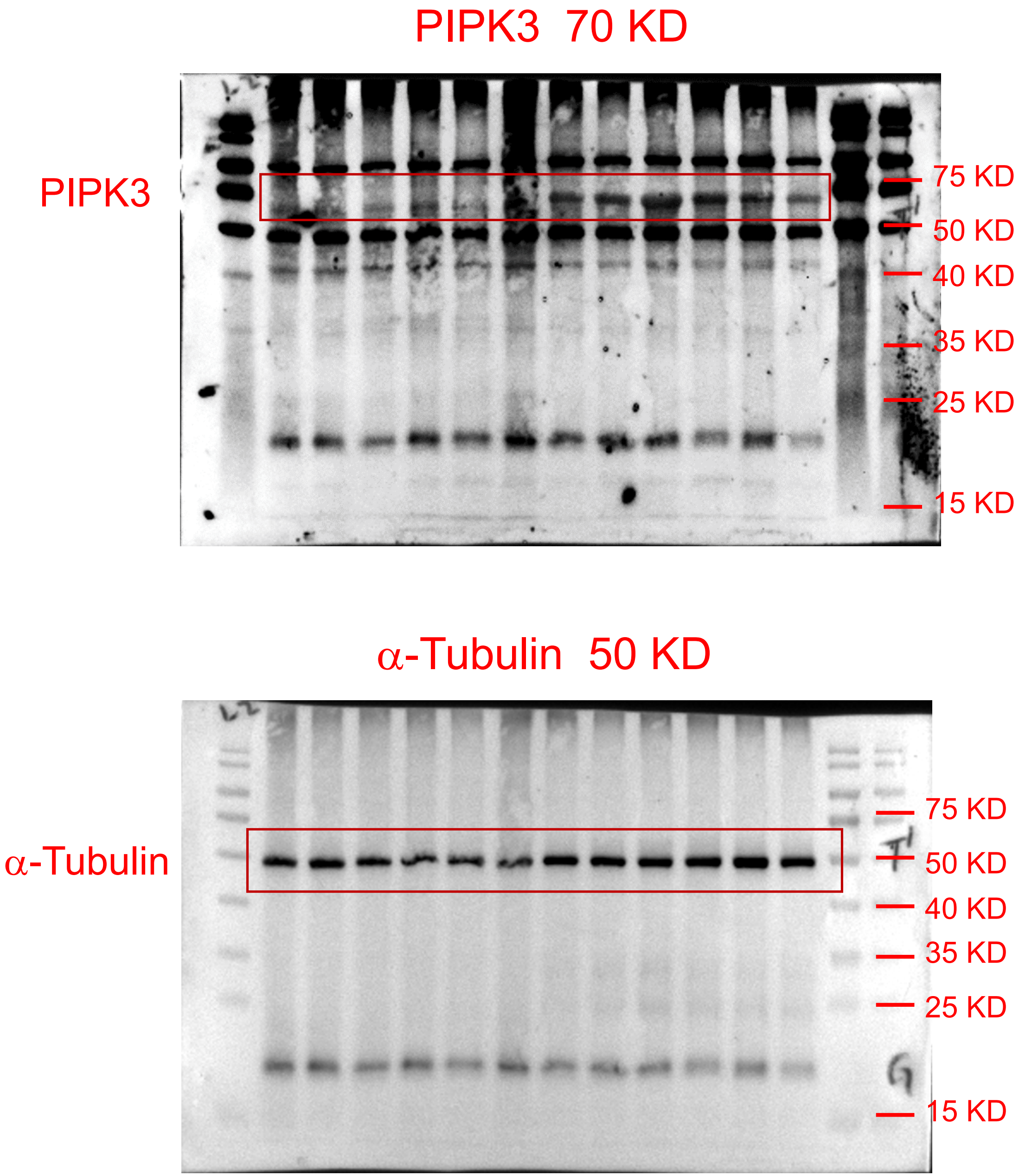
PIPK3 70 KD
PIPK3
75 KD
50 KD
40 KD
35 KD
25 KD
15 KD
α-Tubulin 50 KD
α-Tubulin
75 KD
50 KD
40 KD
35 KD
25 KD
15 KD

Figure 4E

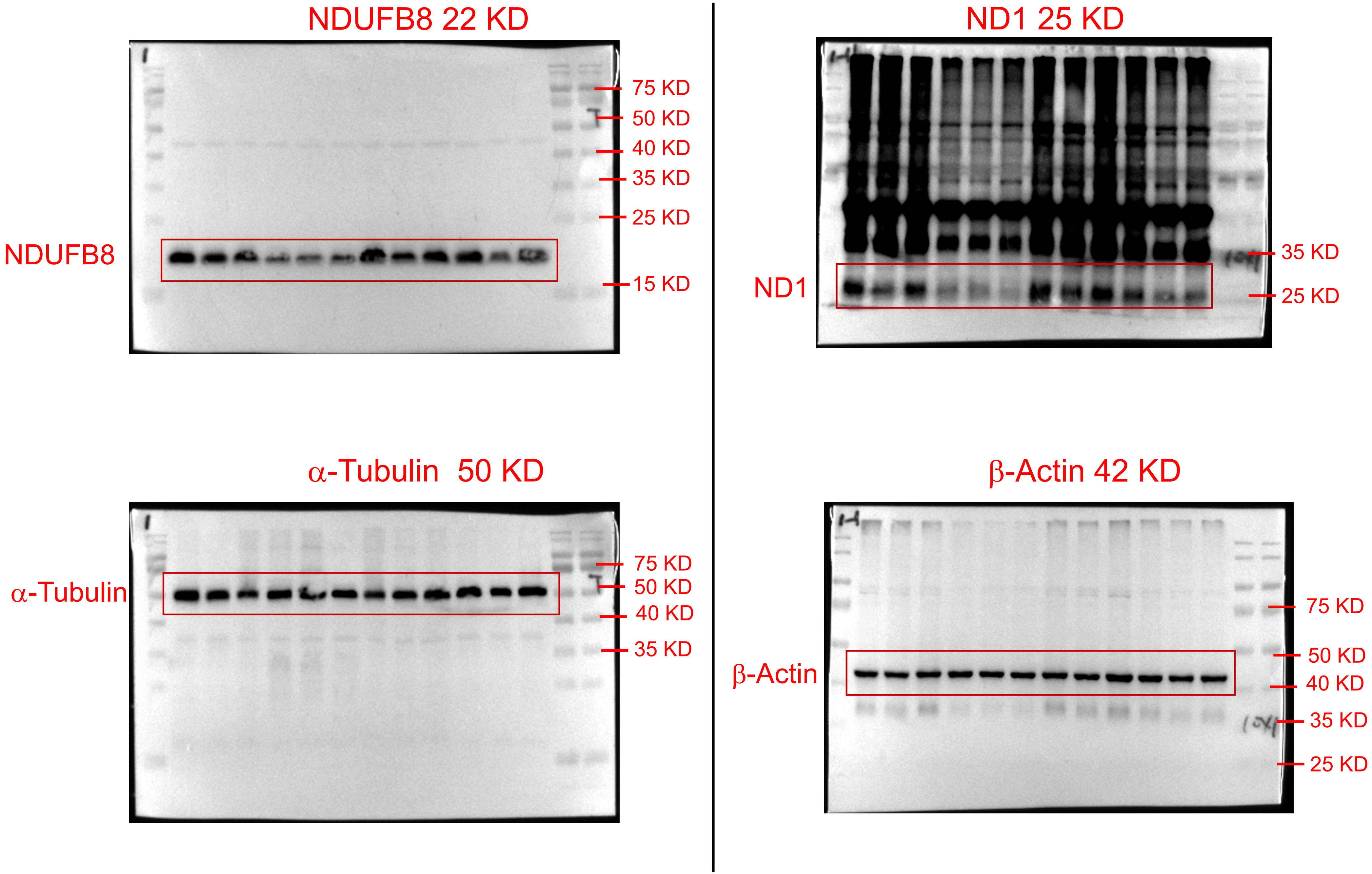
NDUFB8 22 KD
NDUFB8
75 KD
50 KD
40 KD
35 KD
25 KD
15 KD
ND1 25 KD
ND1
35 KD
25 KD
α-Tubulin 50 KD
α-Tubulin
75 KD
50 KD
40 KD
35 KD
β-Actin 42 KD
β-Actin
75 KD
50 KD
40 KD
35 KD
25 KD

Figure 4E

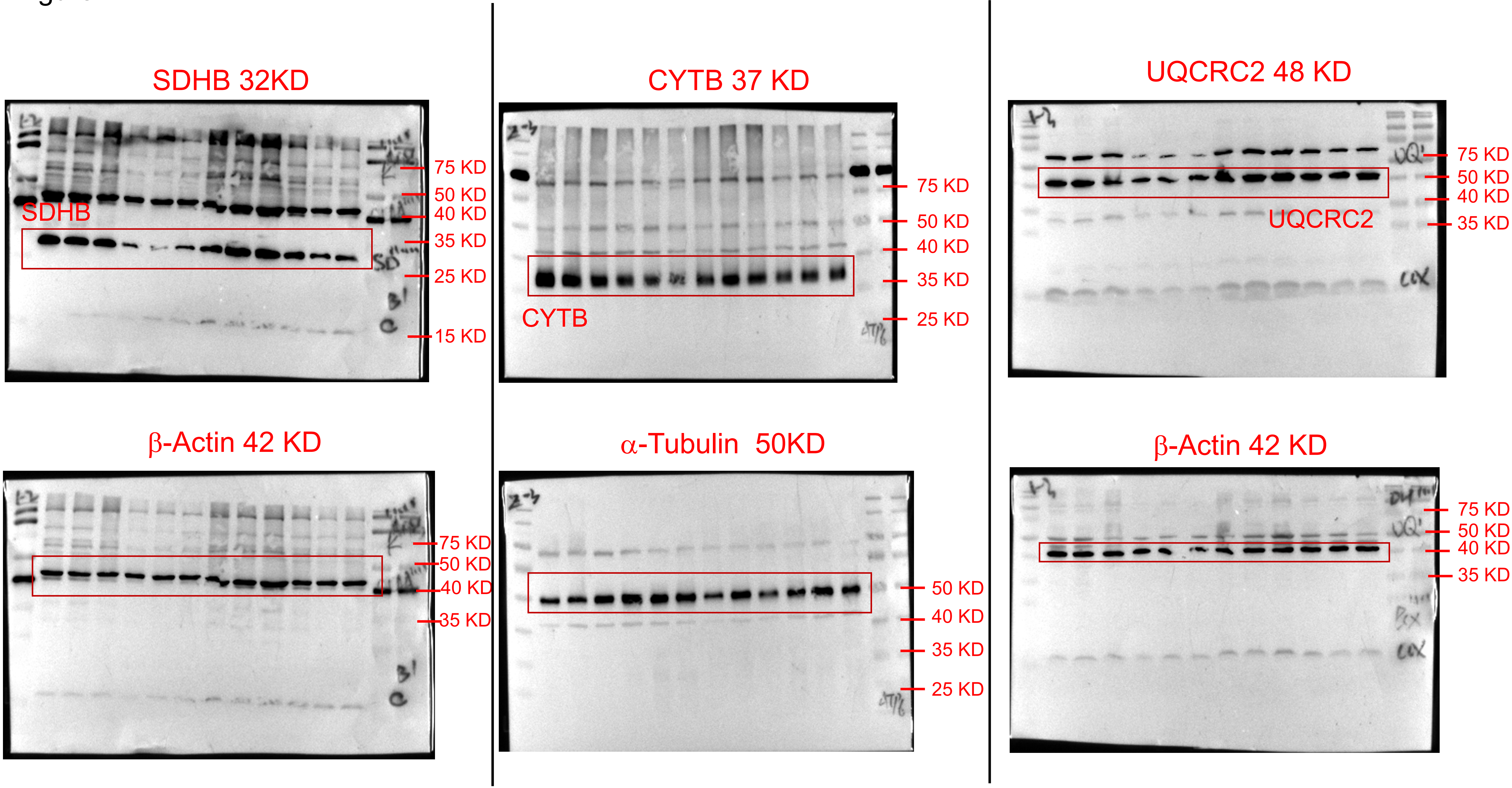
SDHB 32KD
SDHB
75 KD
50 KD
40 KD
35 KD
25 KD
15 KD
CYTB 37 KD
CYTB
75 KD
50 KD
40 KD
35 KD
25 KD
UQCRC2 48 KD
UQCRC2
75 KD
50 KD
40 KD
35 KD
β-Actin 42 KD
75 KD
50 KD
40 KD
35 KD
α-Tubulin 50KD
50 KD
40 KD
35 KD
25 KD
β-Actin 42 KD
75 KD
50 KD
40 KD
35 KD

Figure 4E

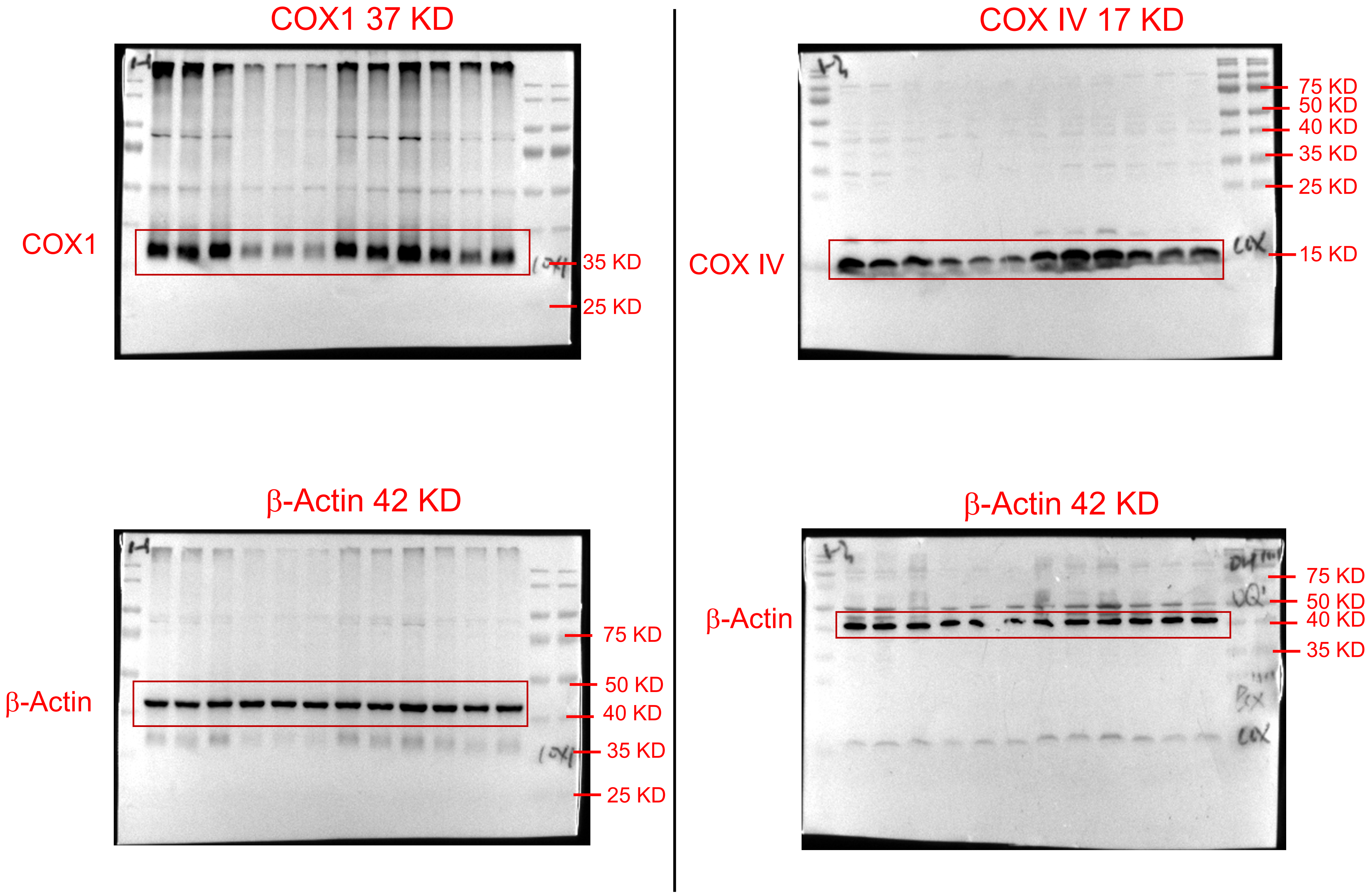

COX1 37 KD
COX1
35 KD
25 KD
COX IV 17 KD
75 KD
50 KD
40 KD
35 KD
25 KD
COX IV
15 KD
β-Actin 42 KD
β-Actin
75 KD
50 KD
40 KD
35 KD
25 KD
β-Actin 42 KD
β-Actin
75 KD
50 KD
40 KD
35 KD

Figure 4E

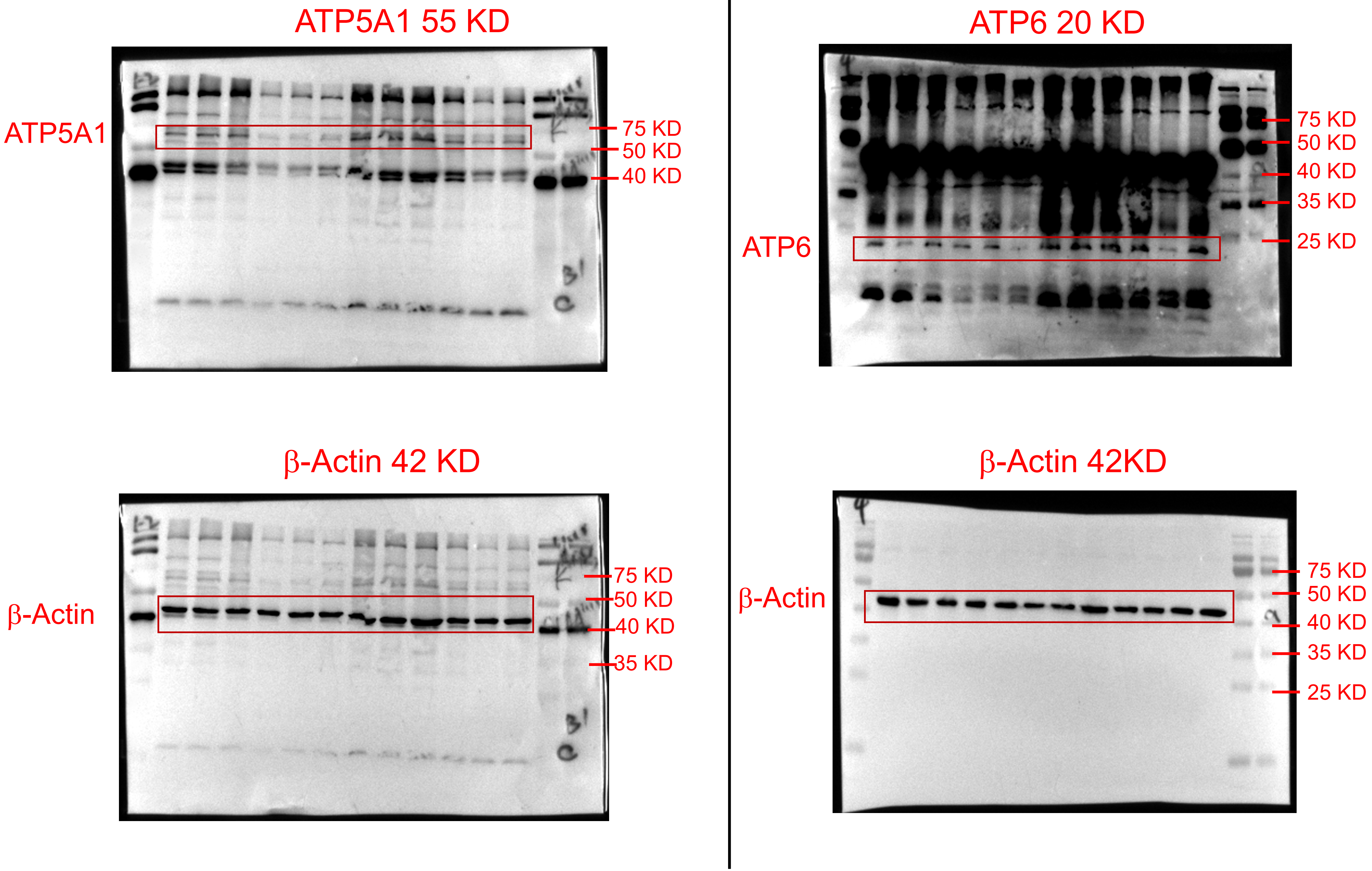
ATP5A1 55 KD
ATP5A1
75 KD
50 KD
40 KD
ATP6 20 KD
ATP6
75 KD
50 KD
40 KD
35 KD
25 KD
β-Actin 42 KD
β-Actin
75 KD
50 KD
40 KD
35 KD
β-Actin 42KD
β-Actin
75 KD
50 KD
40 KD
35 KD
25 KD

Figure 4F

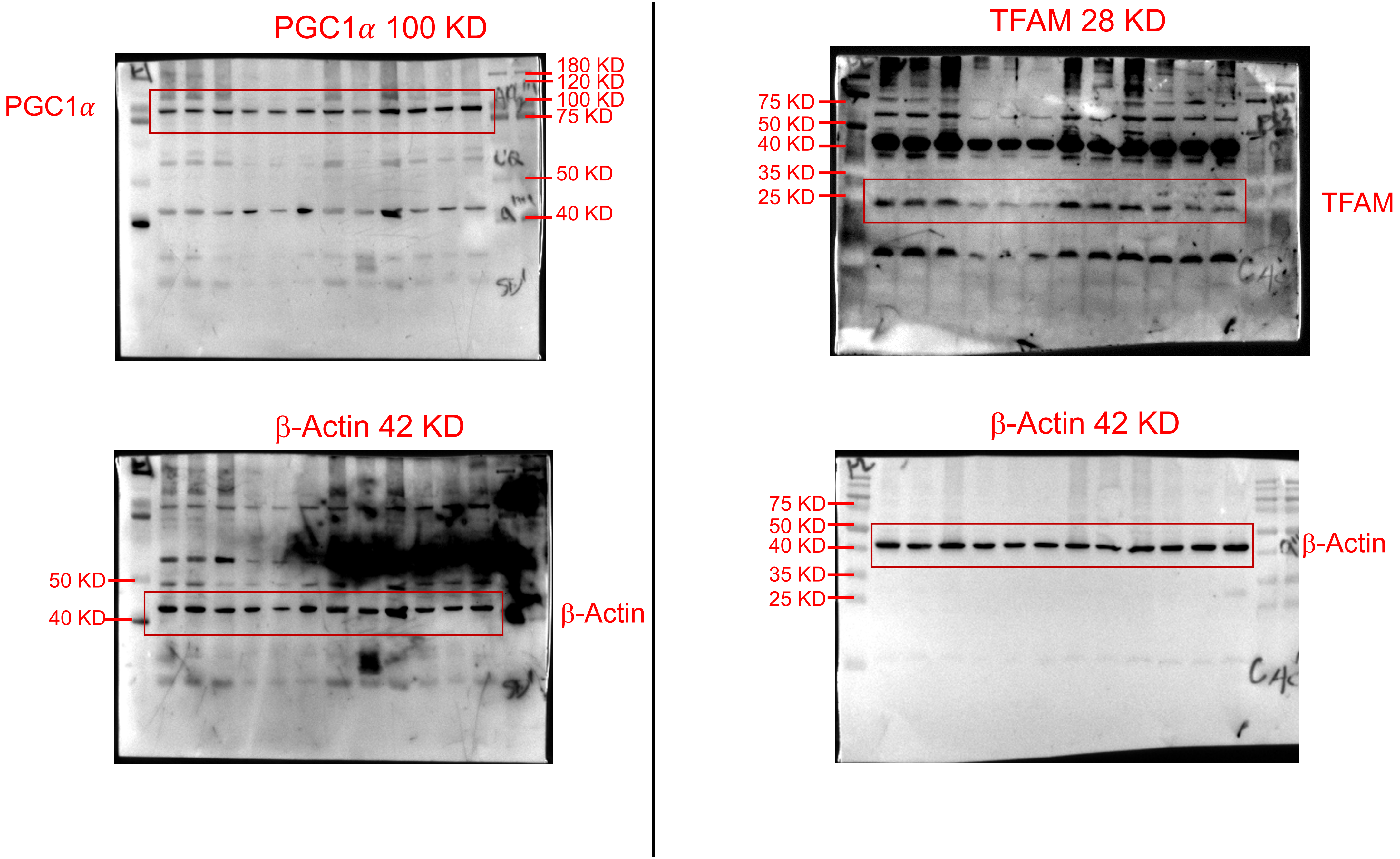
PGC1α 100 KD
PGC1α
180 KD
120 KD
100 KD
75 KD
50 KD
40 KD
TFAM 28 KD
75 KD
50 KD
40 KD
35 KD
25 KD
TFAM
β-Actin 42 KD
50 KD
40 KD
β-Actin
β-Actin 42 KD
75 KD
50 KD
40 KD
35 KD
25 KD
β-Actin

Figure 4H

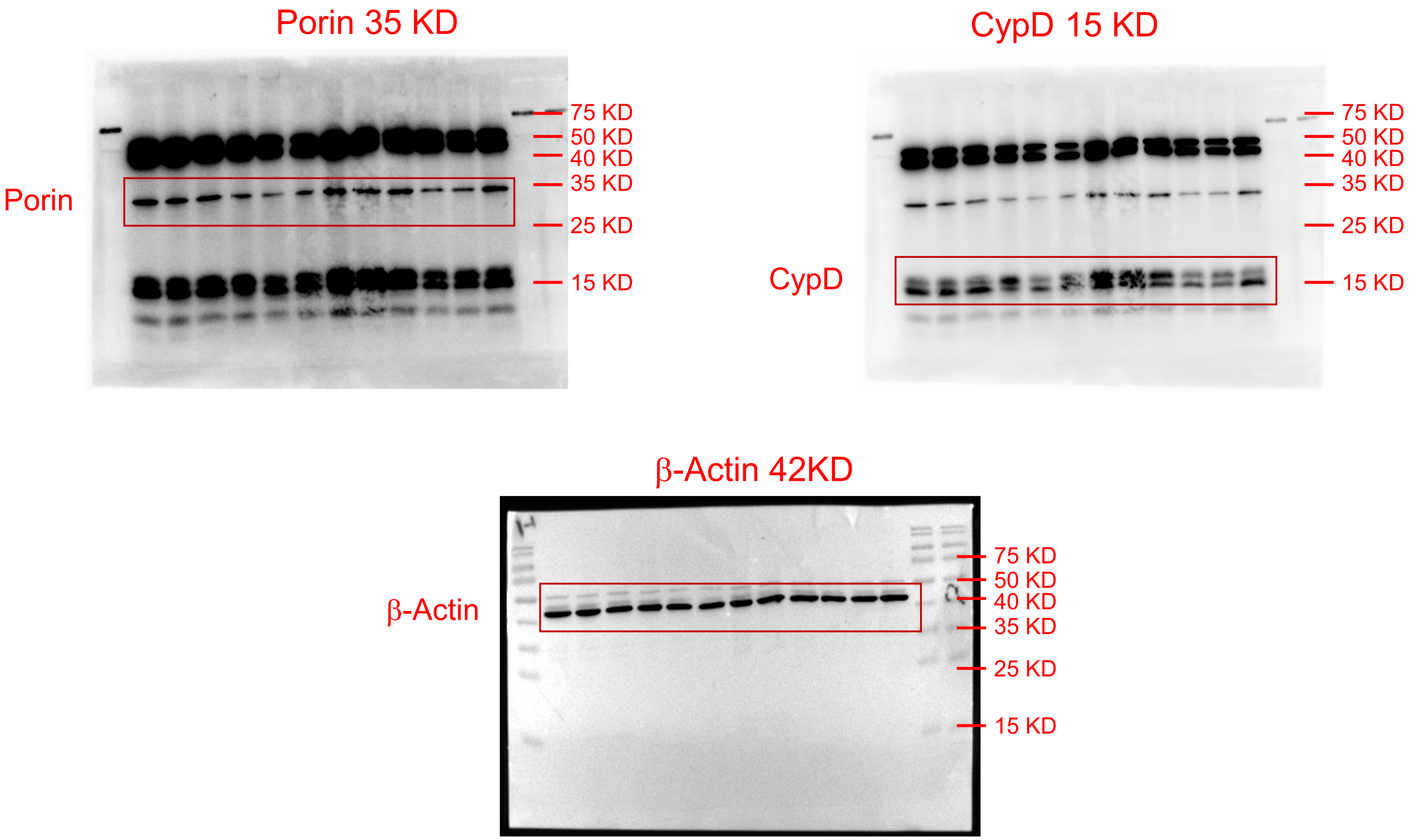

Porin 35 KD
Porin
75 KD
50 KD
40 KD
35 KD
25 KD
15 KD
CypD 15 KD
CypD
75 KD
50 KD
40 KD
35 KD
25 KD
15 KD
β-Actin 42KD
β-Actin
75 KD
50 KD
40 KD
35 KD
25 KD
15 KD

Figure 5H

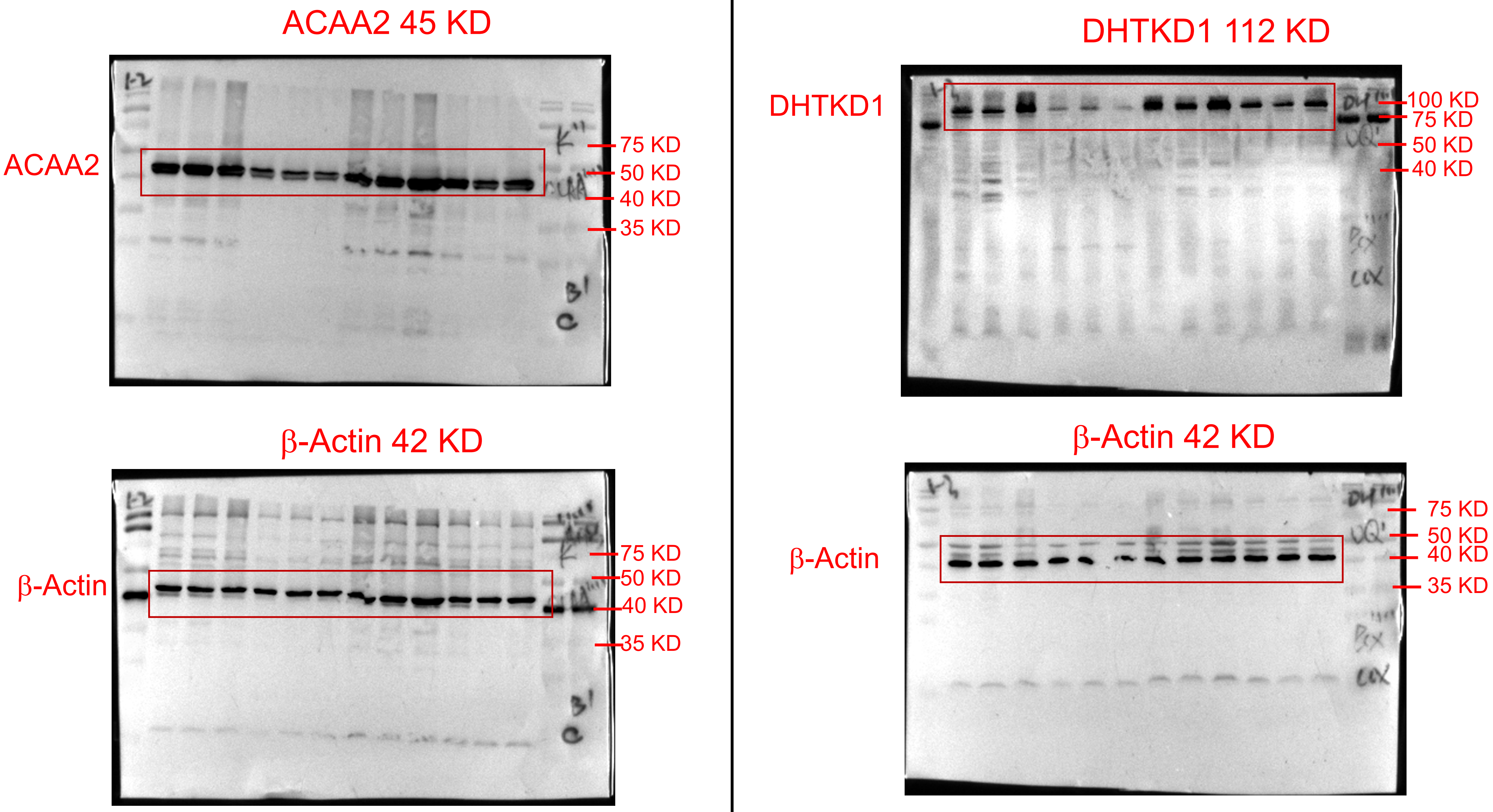
ACAA2 45 KD
ACAA2
75 KD
50 KD
40 KD
35 KD
β-Actin 42 KD
β-Actin
75 KD
50 KD
40 KD
35 KD
DHTKD1 112 KD
DHTKD1
100 KD
75 KD
50 KD
40 KD
β-Actin 42 KD
β-Actin
75 KD
50 KD
40 KD
35 KD

Figure 6E

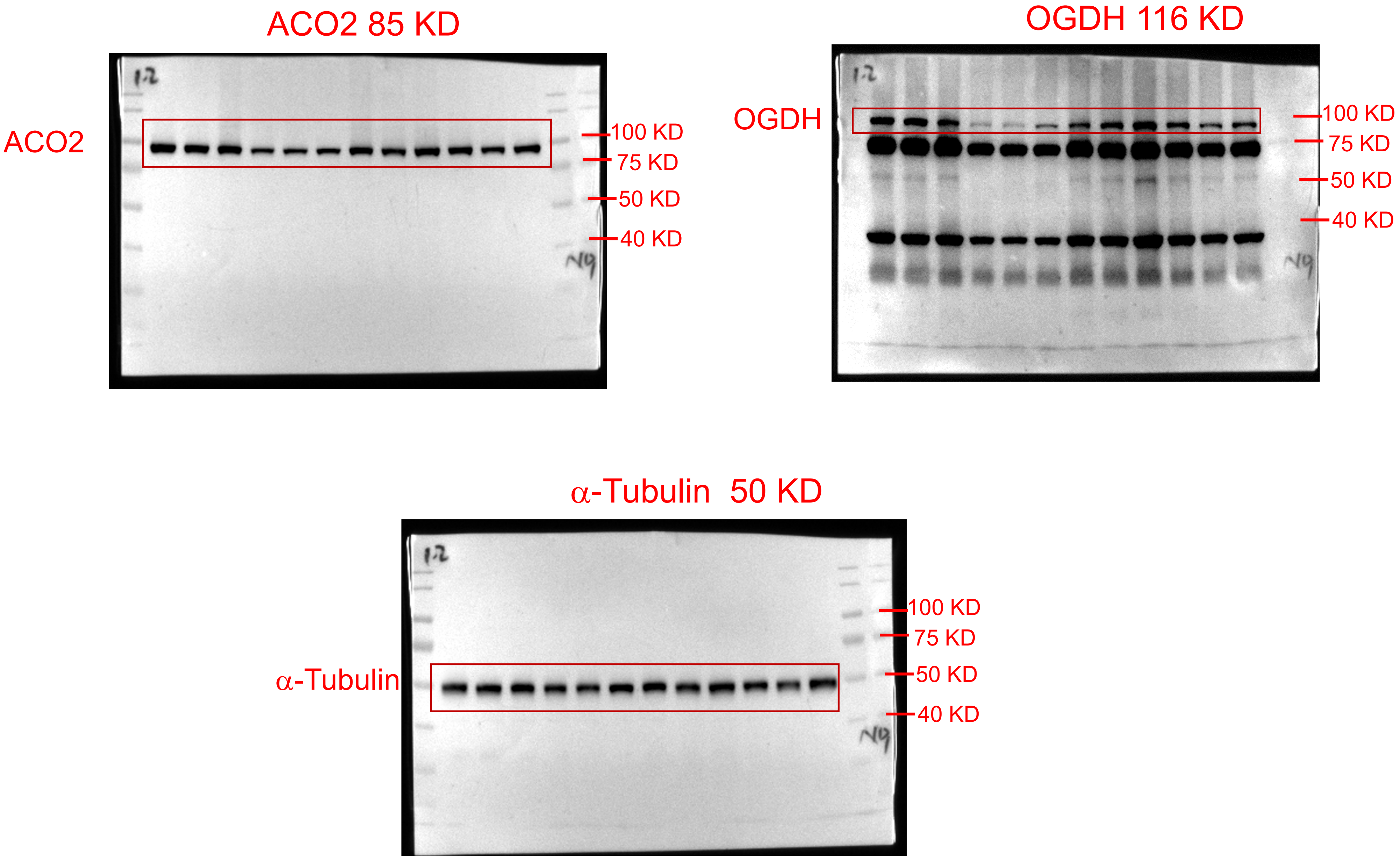
ACO2 85 KD
ACO2
100 KD
75 KD
50 KD
40 KD
OGDH 116 KD
OGDH
100 KD
75 KD
50 KD
40 KD
α-Tubulin 50 KD
α-Tubulin
100 KD
75 KD
50 KD
40 KD

Figure 6E

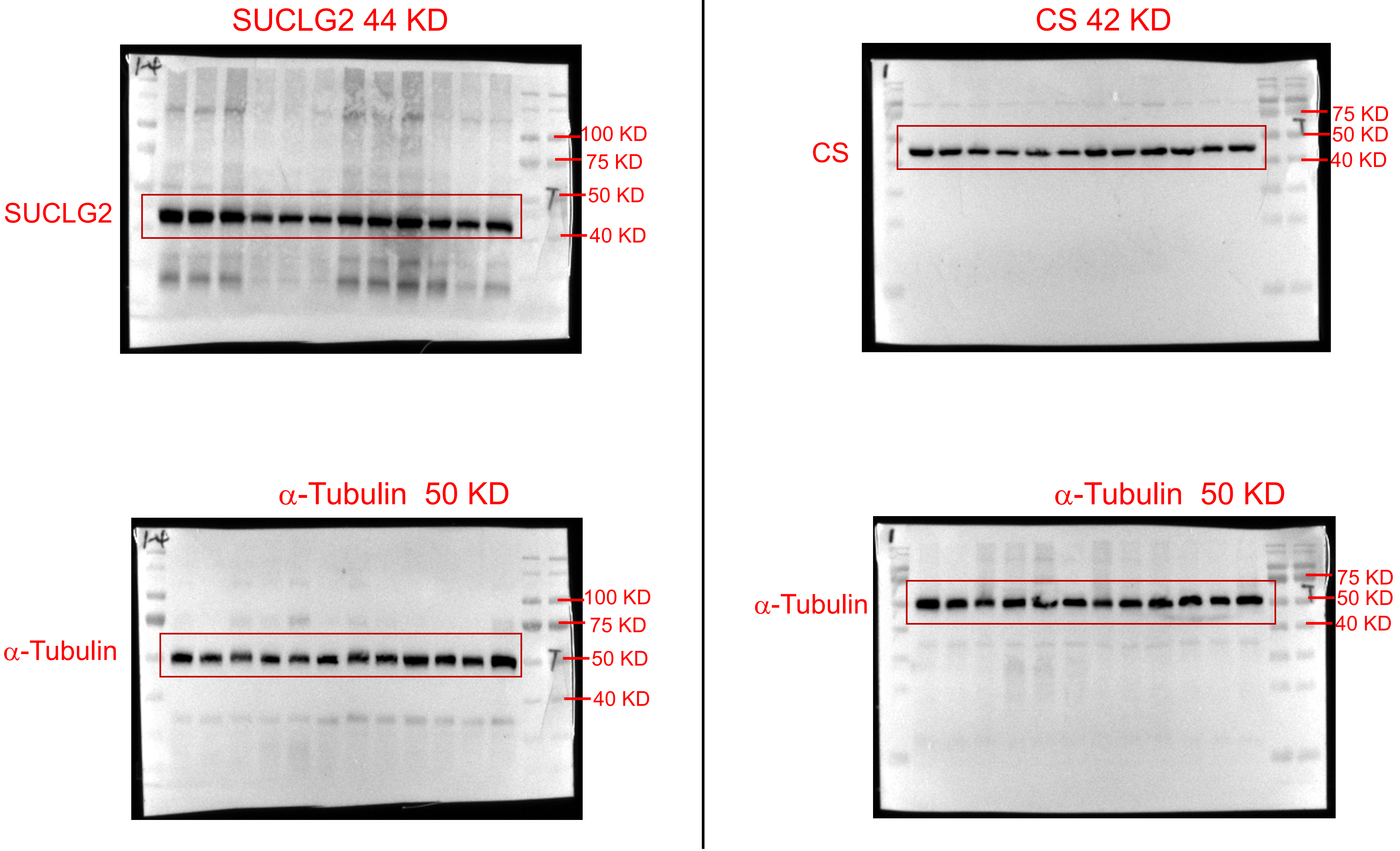
SUCLG2 44 KD
SUCLG2
100 KD
75 KD
50 KD
40 KD
CS 42 KD
CS
75 KD
50 KD
40 KD
α-Tubulin 50 KD
α-Tubulin
100 KD
75 KD
50 KD
40 KD
α-Tubulin 50 KD
α-Tubulin
75 KD
50 KD
40 KD

Figure S1G

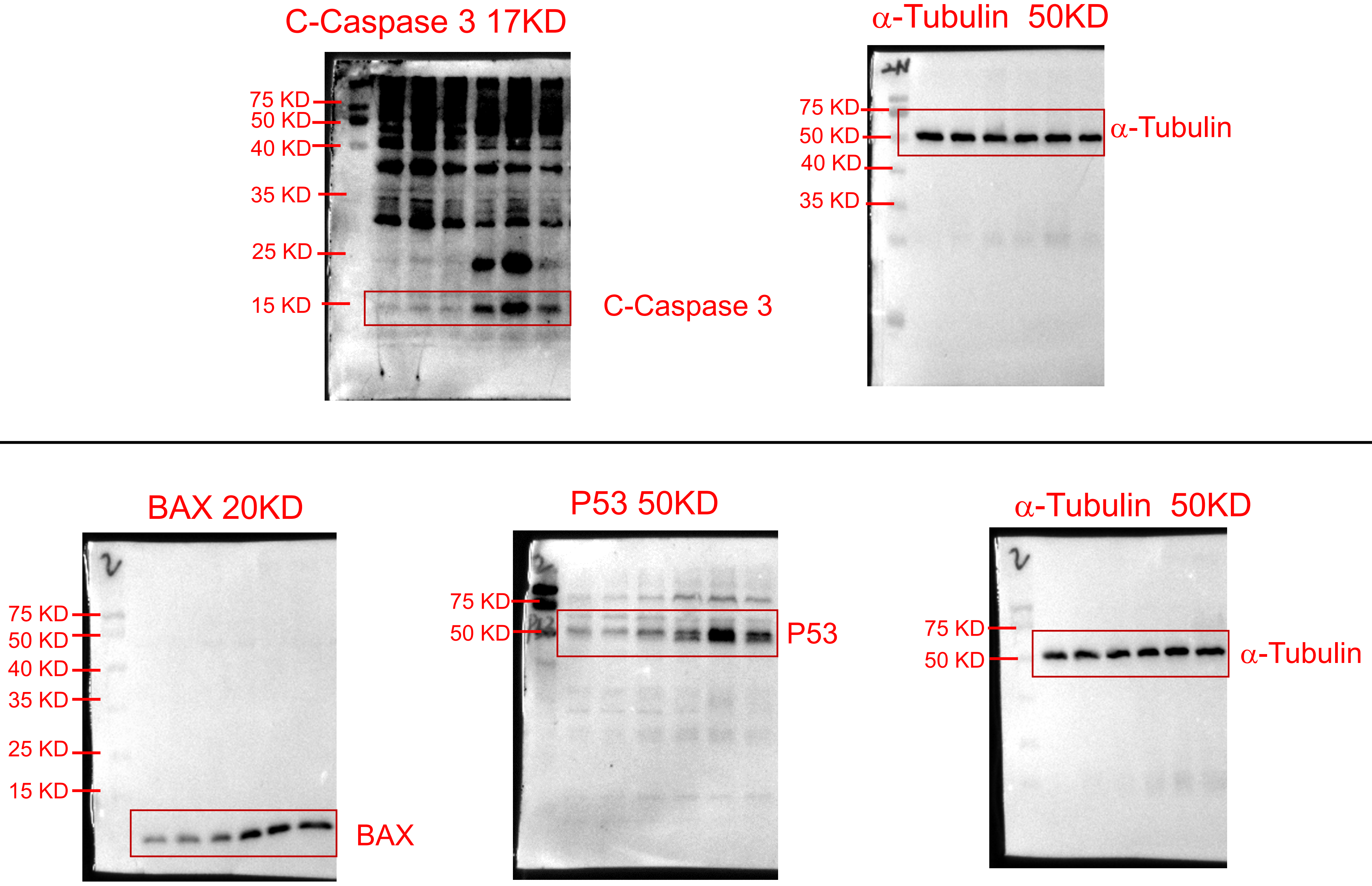
C-Caspase 3 17KD
75 KD
50 KD
40 KD
35 KD
25 KD
15 KD
C-Caspase 3
α-Tubulin 50KD
75 KD
50 KD
40 KD
35 KD
α-Tubulin
BAX 20KD
75 KD
50 KD
40 KD
35 KD
25 KD
15 KD
BAX
P53 50KD
75 KD
50 KD
P53
α-Tubulin 50KD
75 KD
50 KD
α-Tubulin

Figure S1H

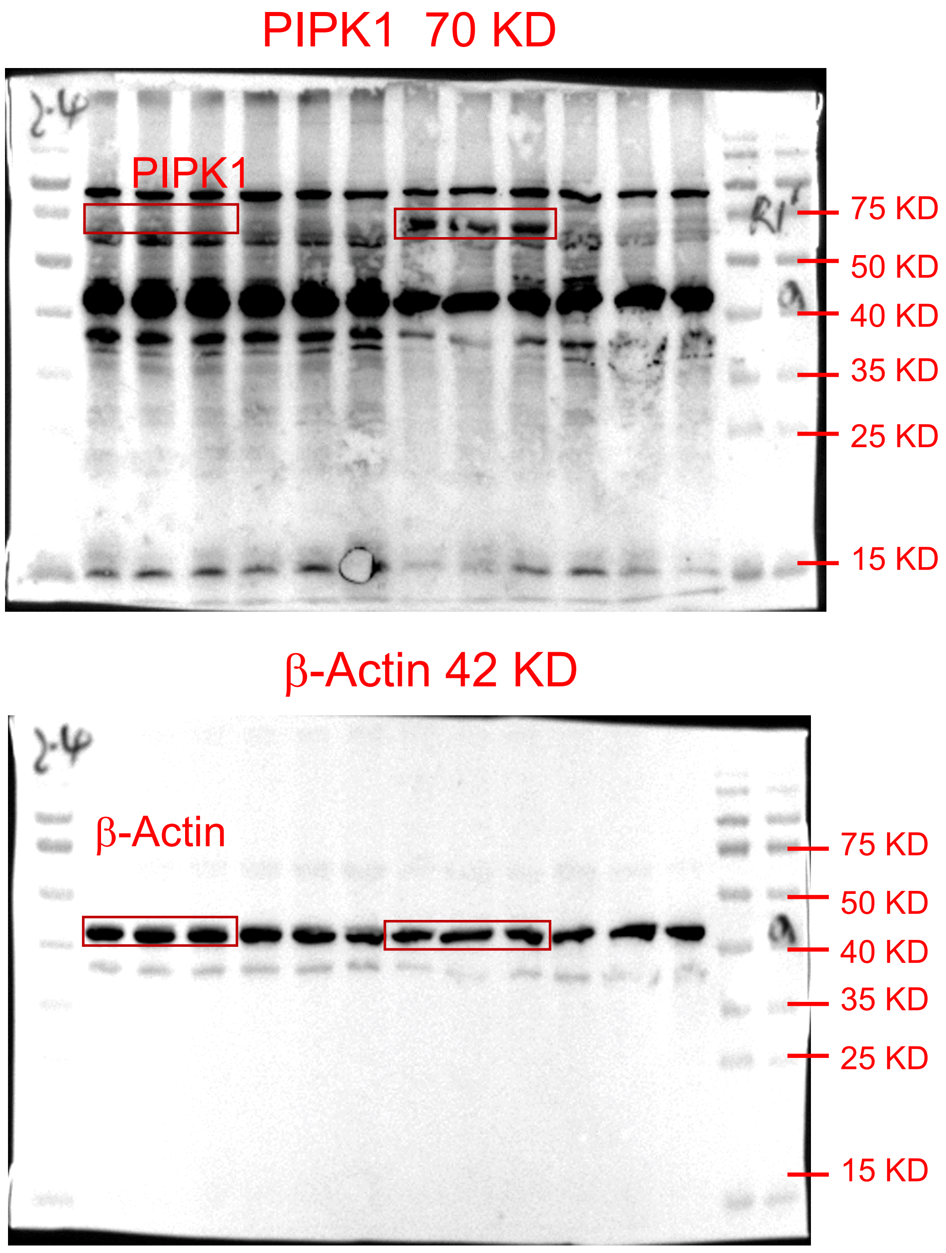

Figure S1H

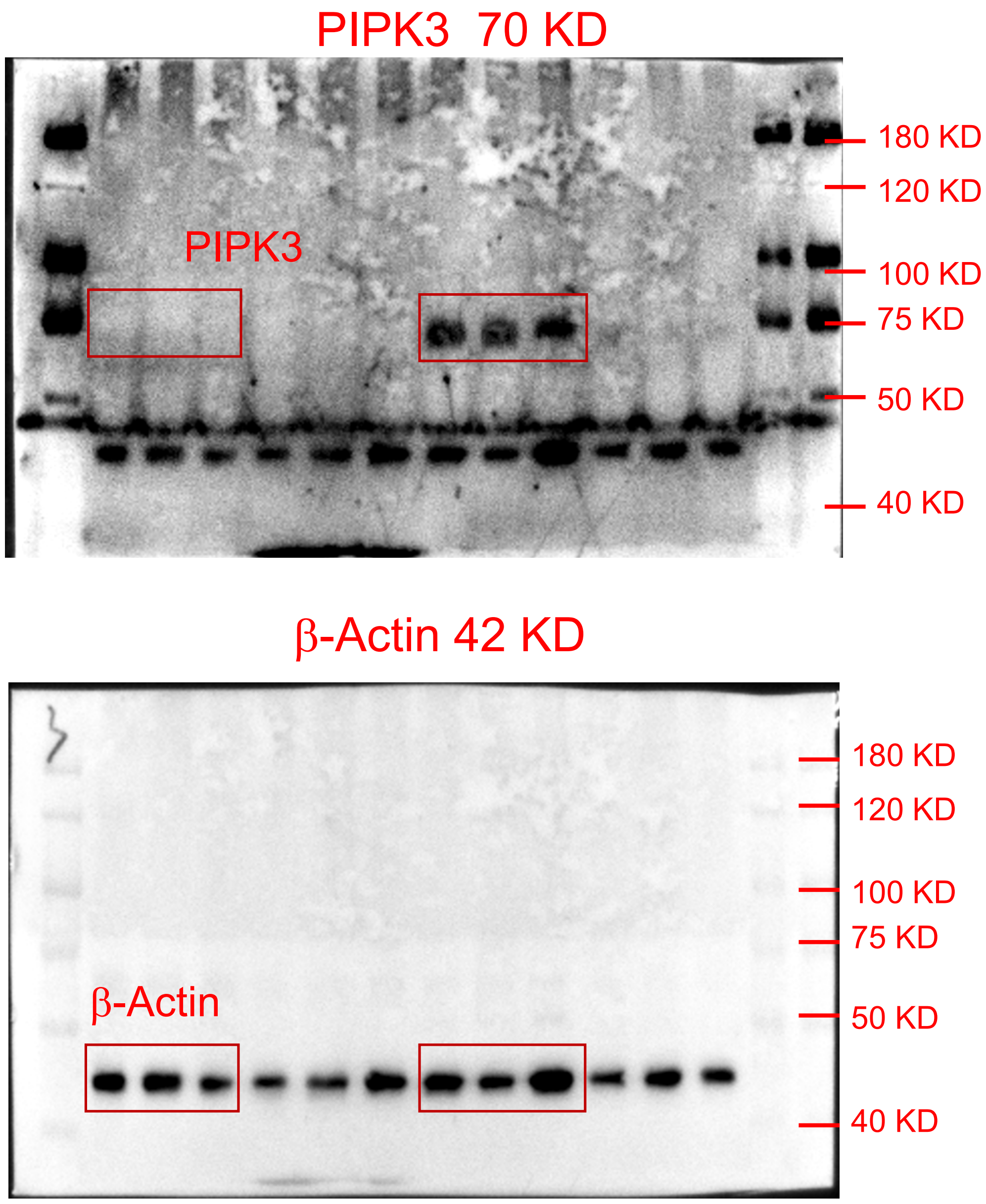

Figure S2B

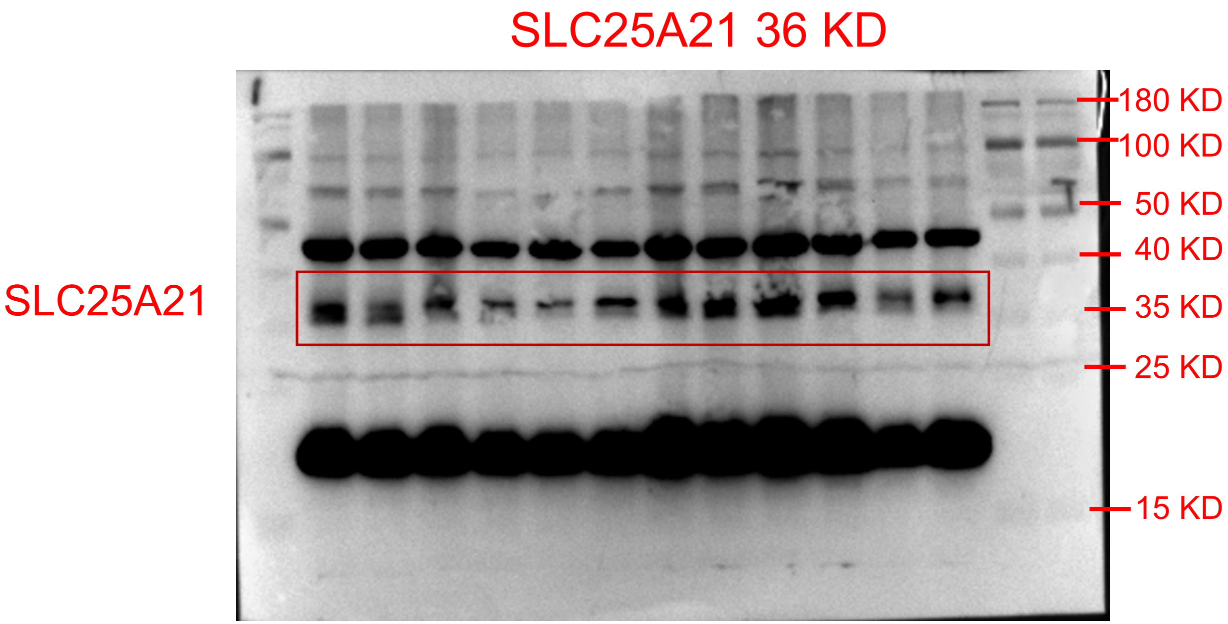
SLC25A21 36 KD
SLC25A21
180 KD
100 KD
50 KD
40 KD
35 KD
25 KD
15 KD

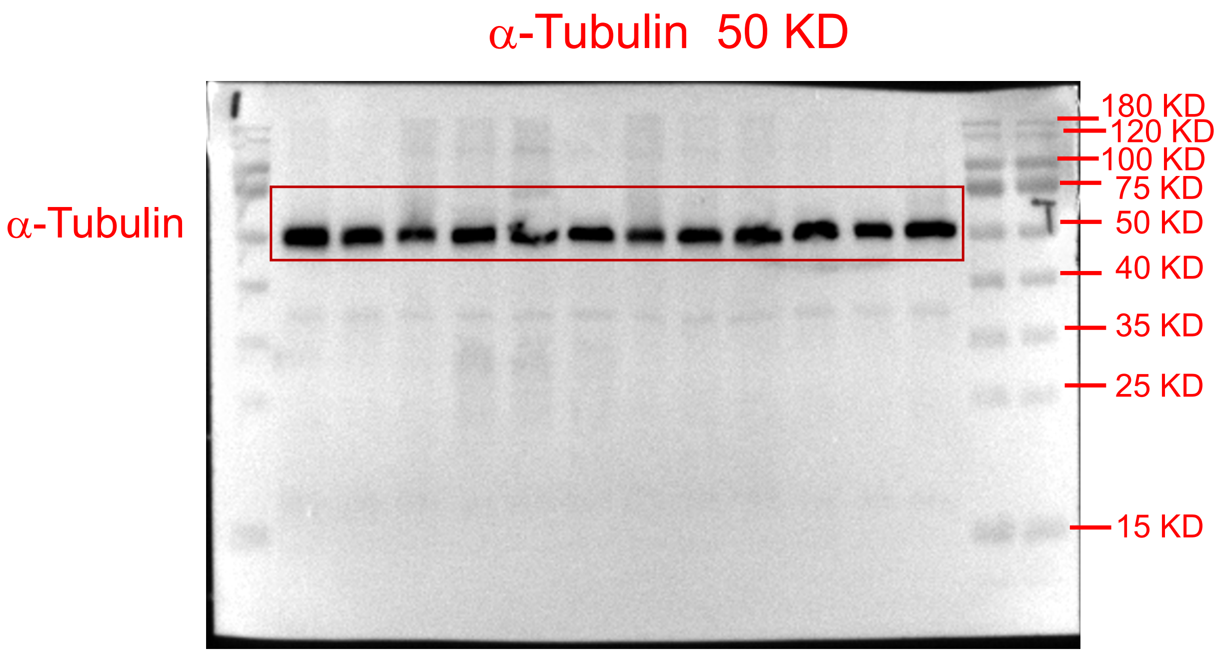
α-Tubulin 50 KD
α-Tubulin
180 KD
120 KD
100 KD
75 KD
50 KD
40 KD
35 KD
25 KD
15 KD

Figure S3B

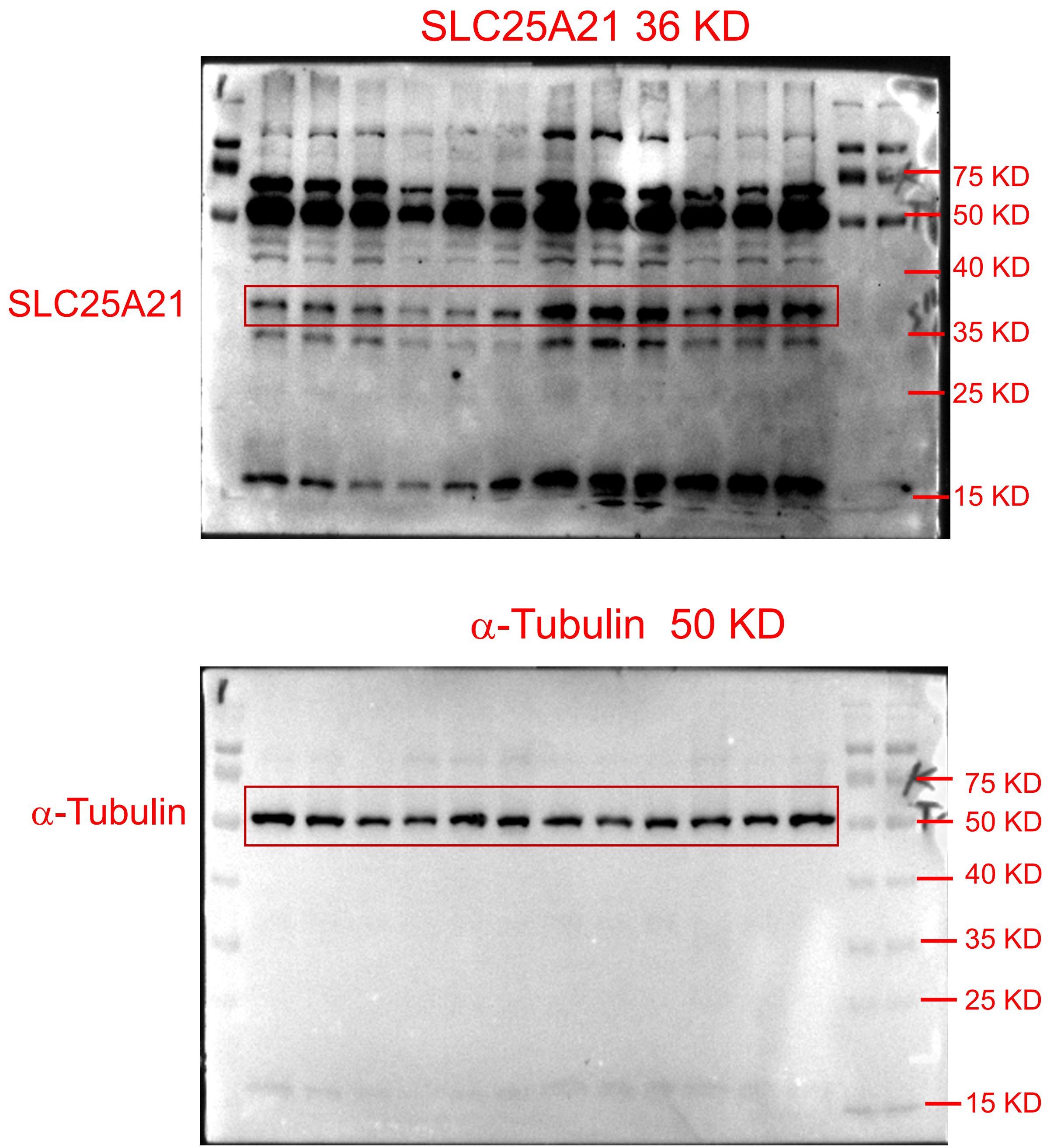

Figure S3D

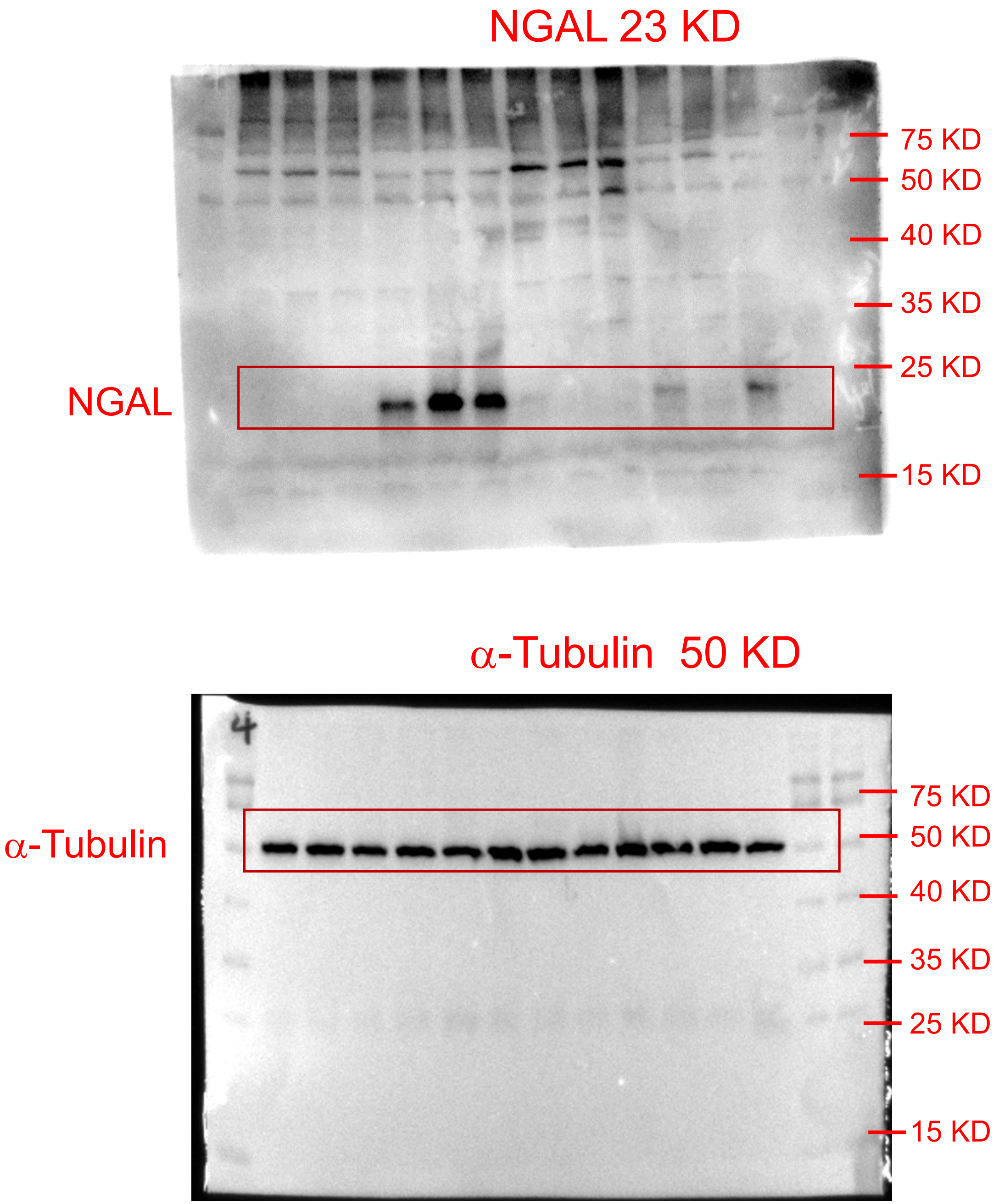

Figure S3G

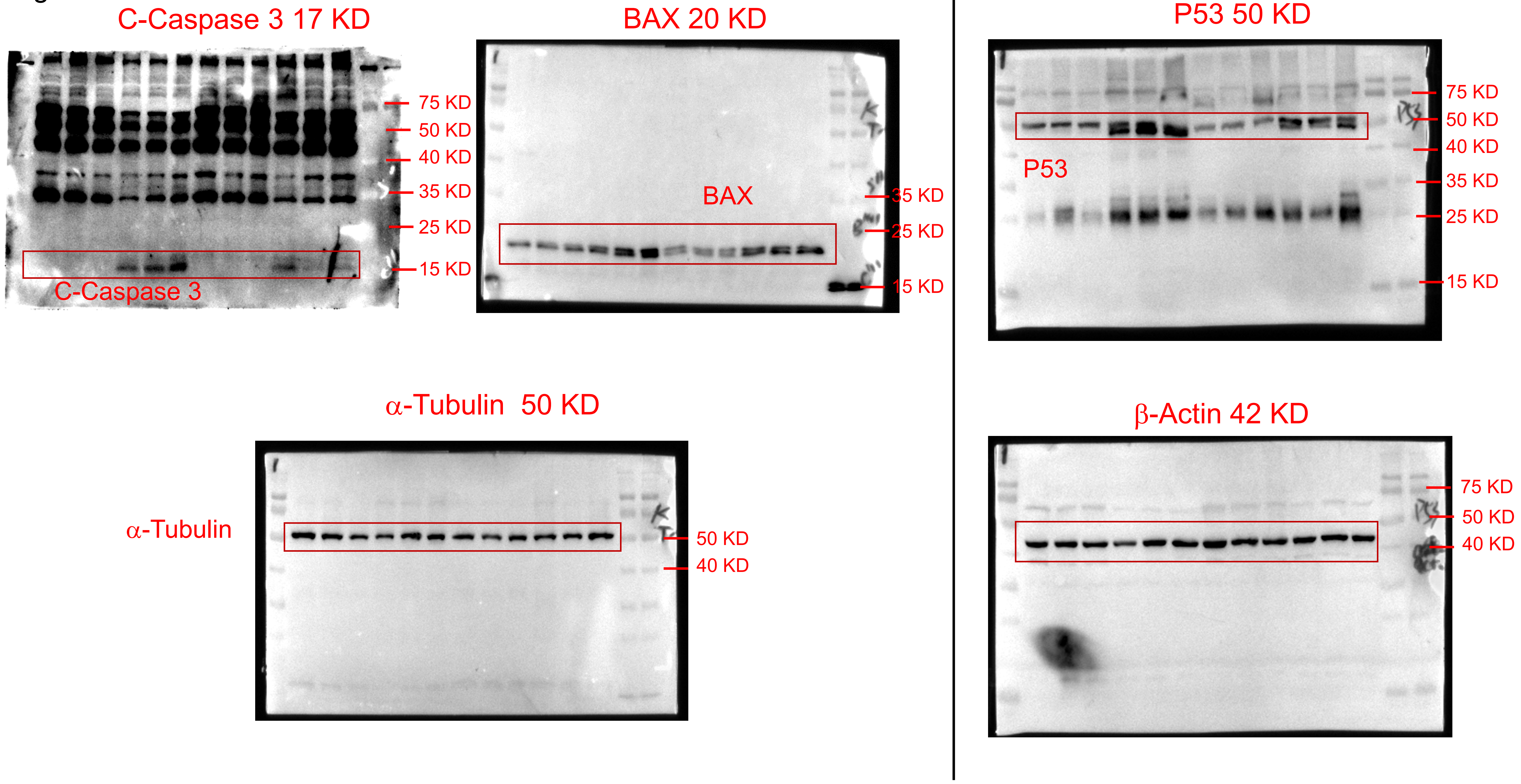

C-Caspase 3 17 KD
75 KD
50 KD
40 KD
35 KD
25 KD
15 KD
C-Caspase 3
BAX 20 KD
BAX
35 KD
25 KD
15 KD
P53 50 KD
P53
75 KD
50 KD
40 KD
35 KD
25 KD
15 KD
α-Tubulin 50 KD
α-Tubulin
50 KD
40 KD
β-Actin 42 KD
75 KD
50 KD
40 KD

Figure S3H

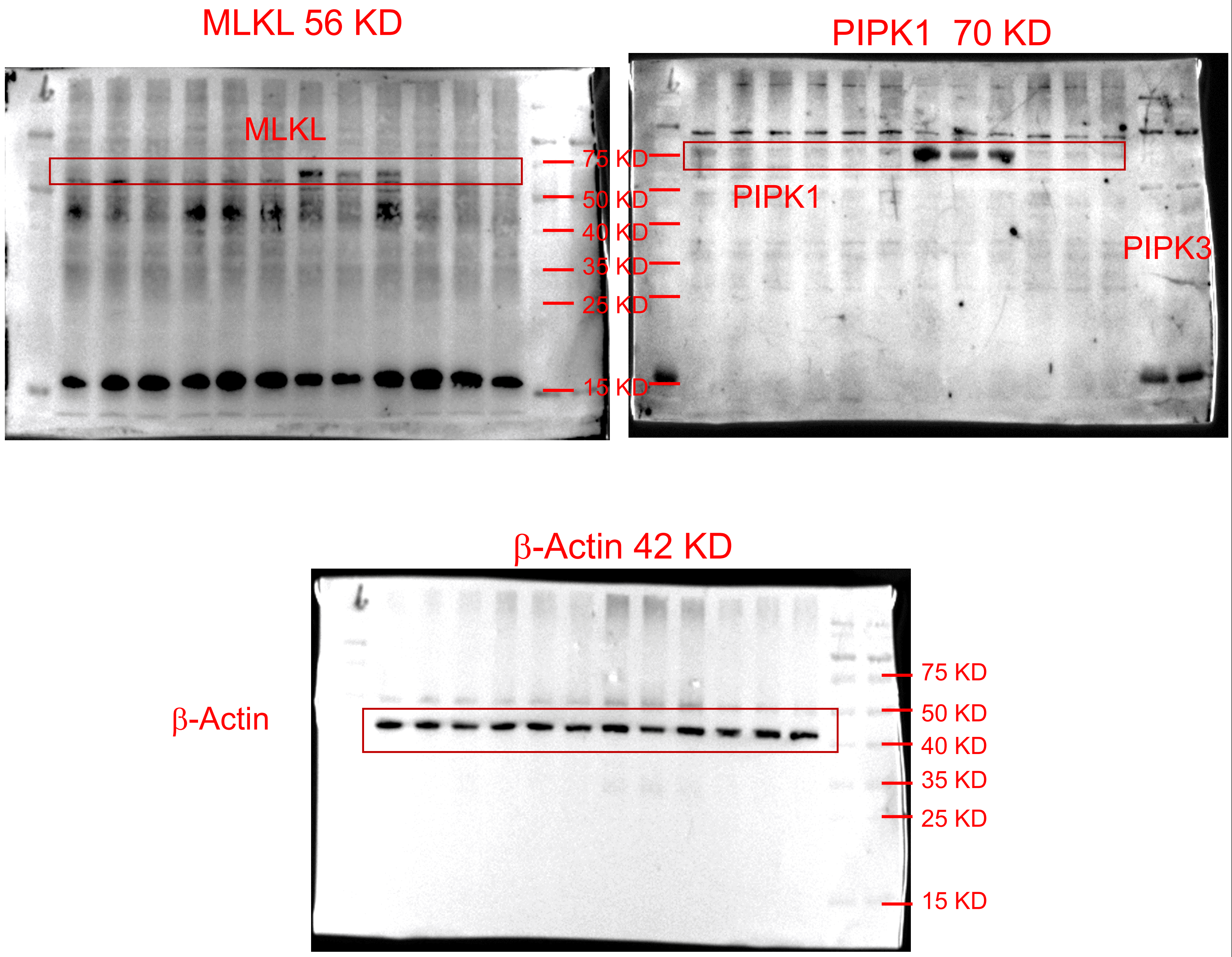

Figure S3H

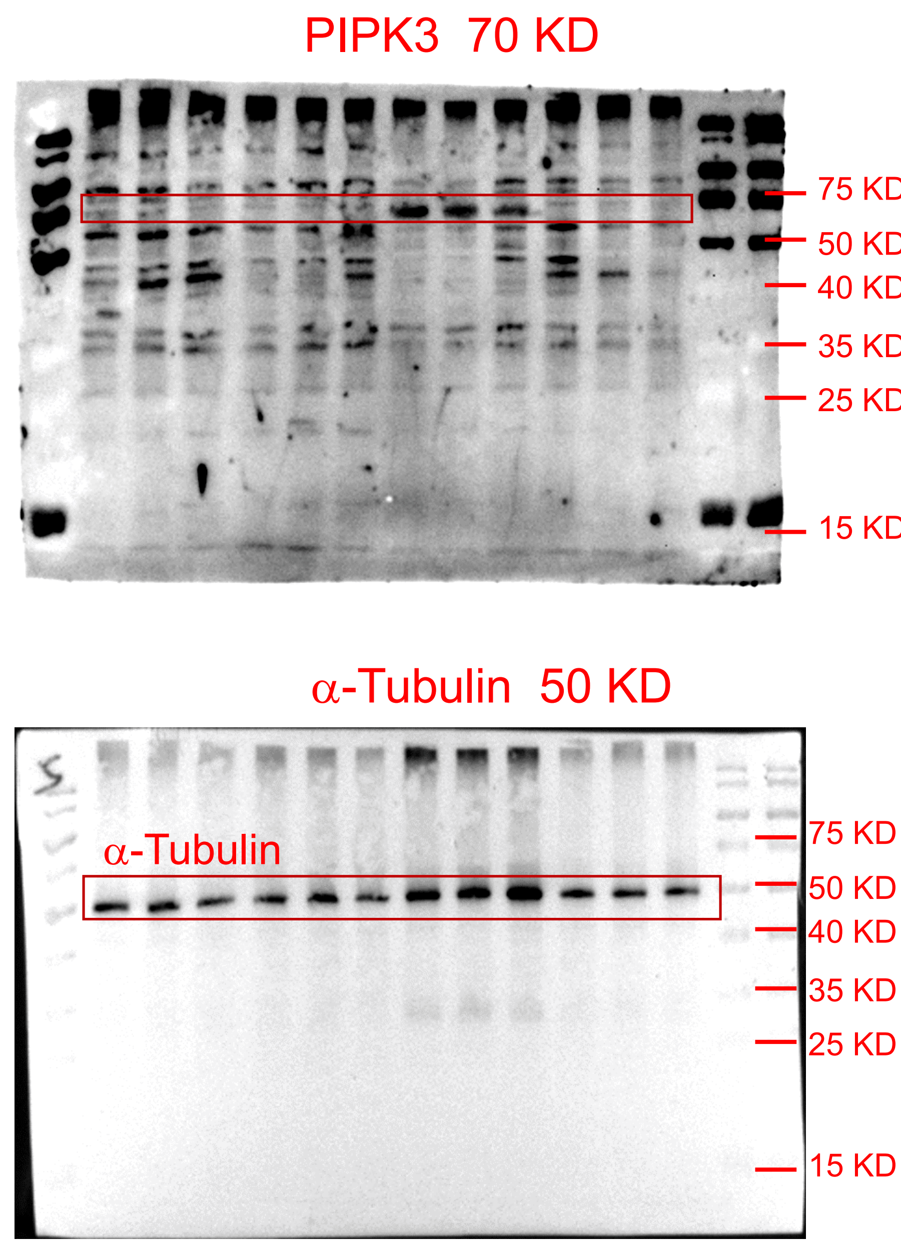

Figure S4B

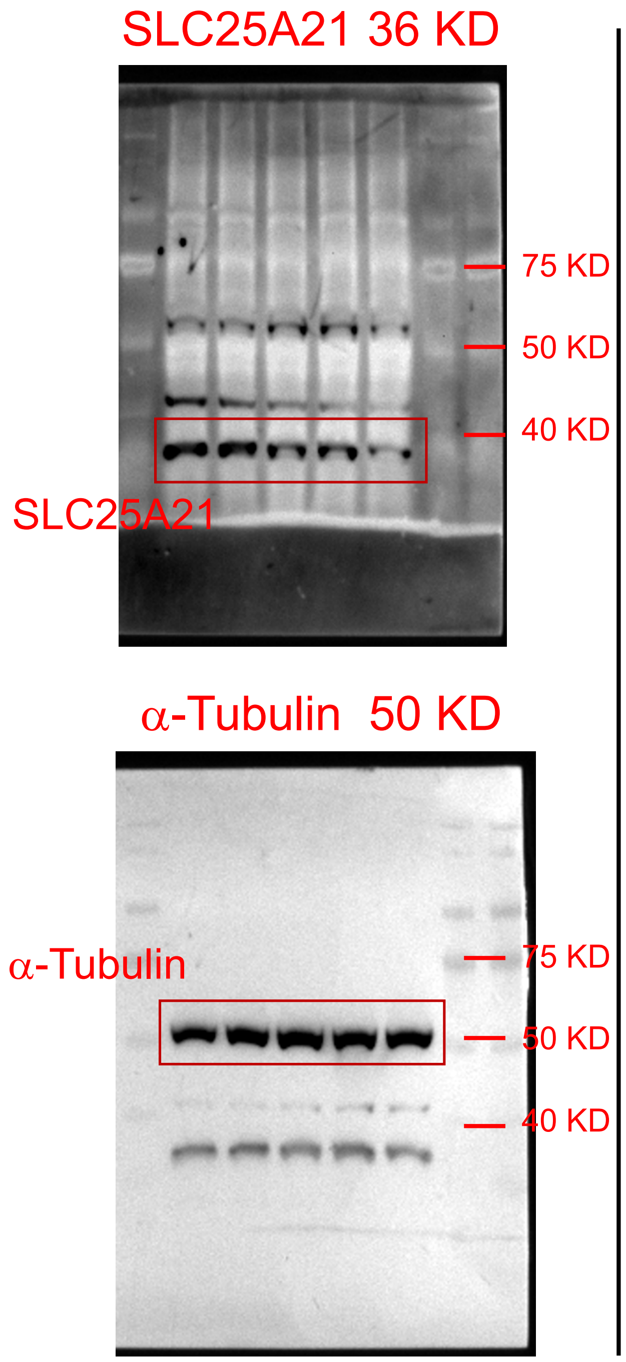

Figure S4C

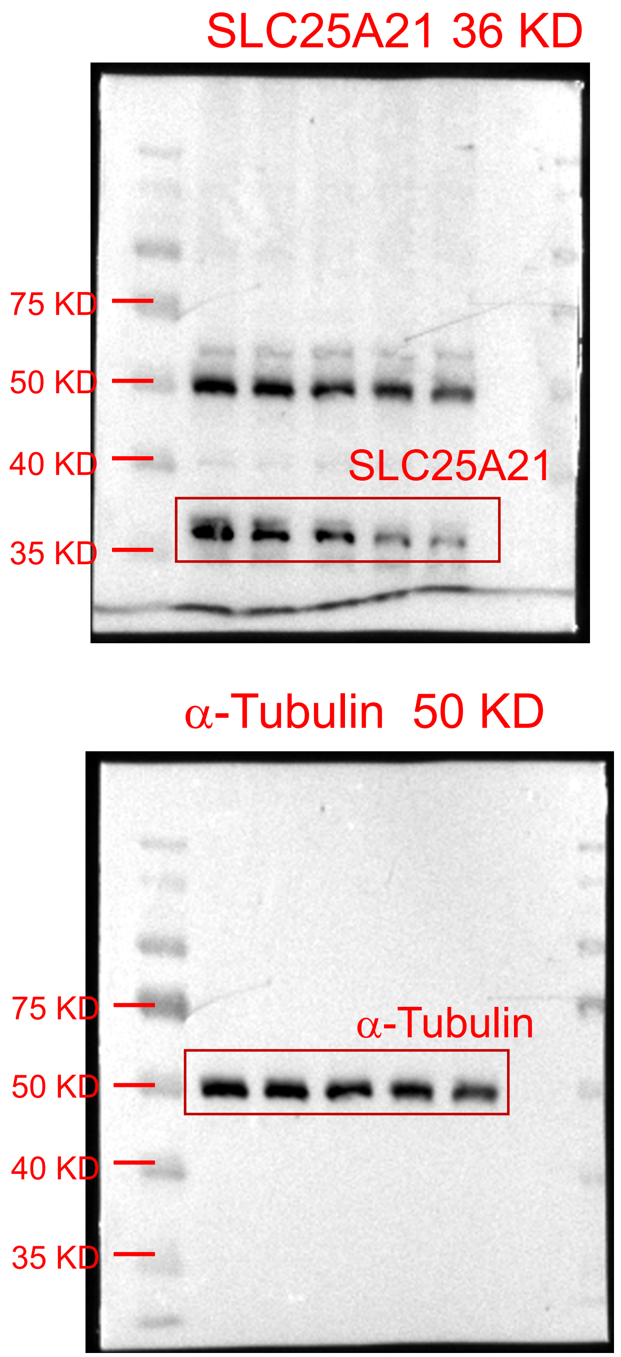

Figure S5A

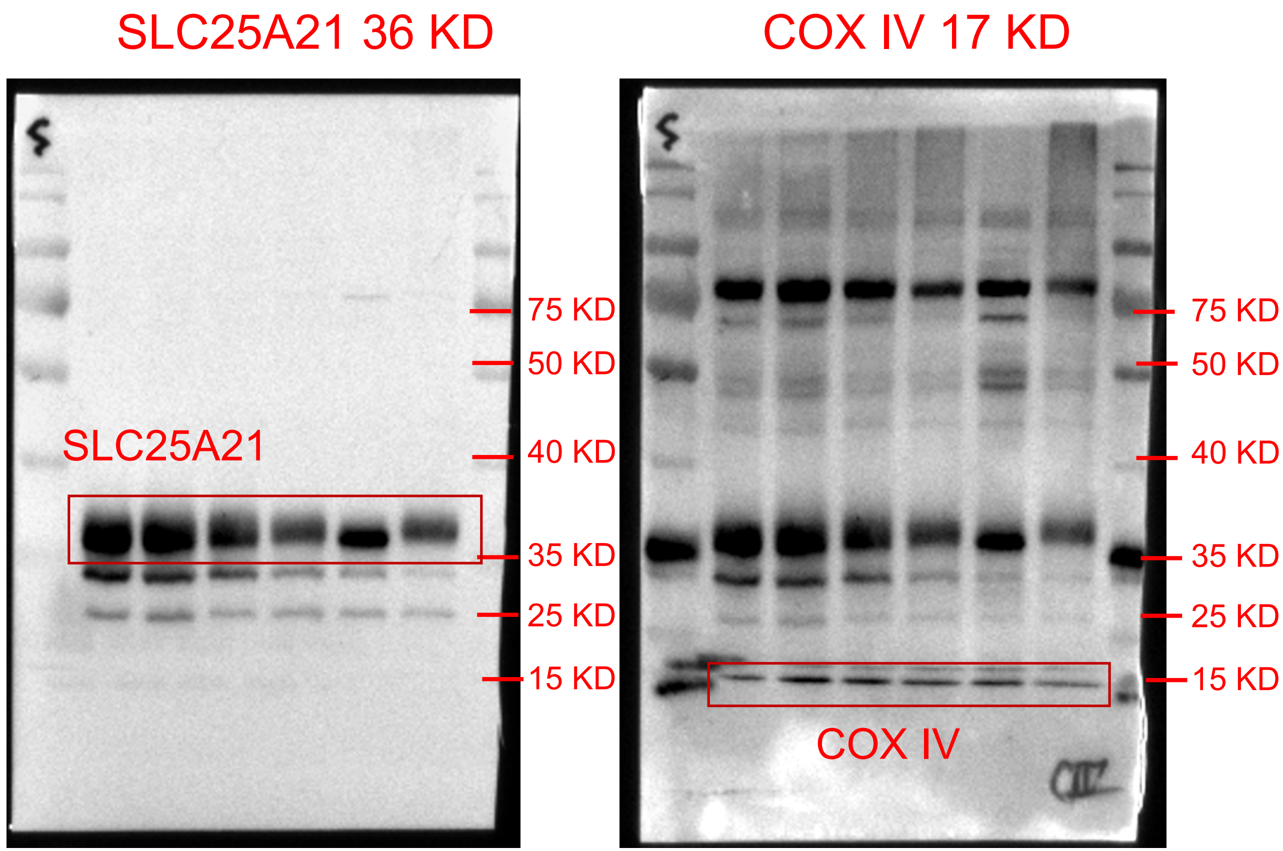

Figure S5D

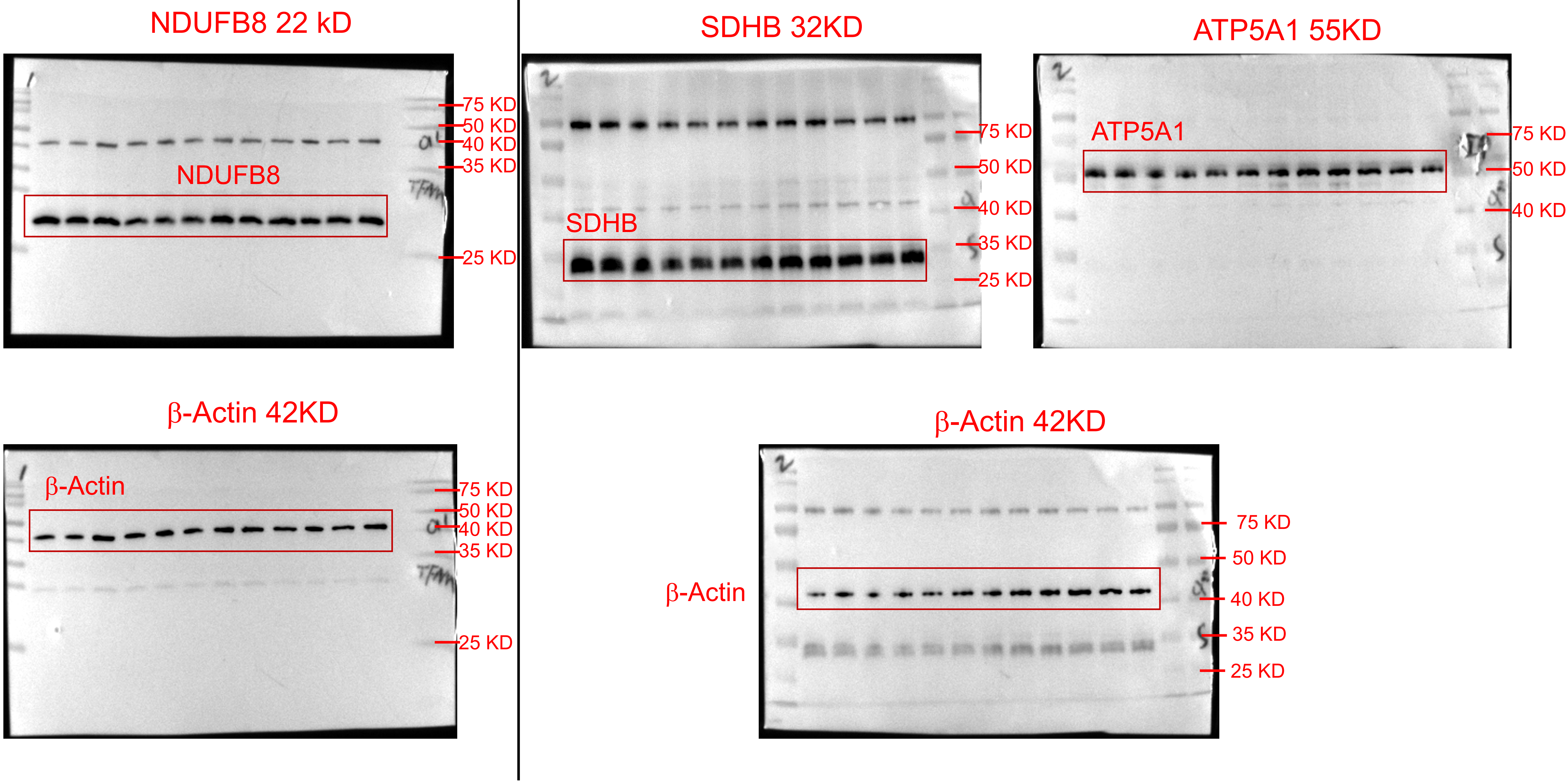

NDUFB8 22 kD
NDUFB8
75 KD
50 KD
40 KD
35 KD
25 KD
SDHB 32KD
SDHB
75 KD
50 KD
40 KD
35 KD
25 KD
ATP5A1 55KD
ATP5A1
75 KD
50 KD
40 KD
β-Actin 42KD
β-Actin
75 KD
50 KD
40 KD
35 KD
25 KD
β-Actin 42KD
β-Actin
75 KD
50 KD
40 KD
35 KD
25 KD

Figure S5D

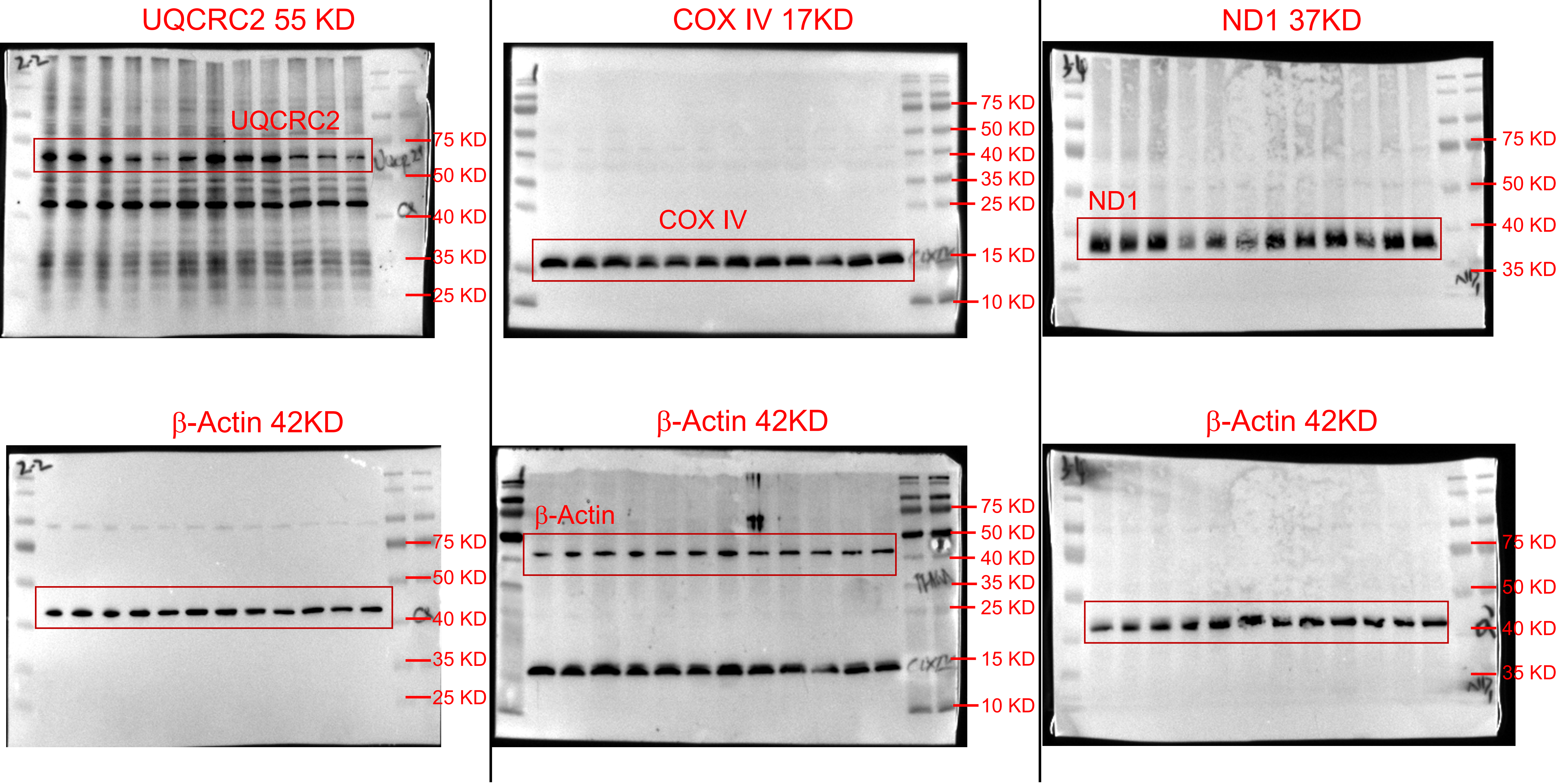
UQCRC2 55 KD
UQCRC2
75 KD
50 KD
40 KD
35 KD
25 KD
COX IV 17KD
COX IV
75 KD
50 KD
40 KD
35 KD
25 KD
15 KD
10 KD
ND1 37KD
ND1
75 KD
50 KD
40 KD
35 KD
β-Actin 42KD
75 KD
50 KD
40 KD
35 KD
25 KD
β-Actin 42KD
β-Actin
75 KD
50 KD
40 KD
35 KD
25 KD
15 KD
10 KD
β-Actin 42KD
75 KD
50 KD
40 KD
35 KD

Figure S5D

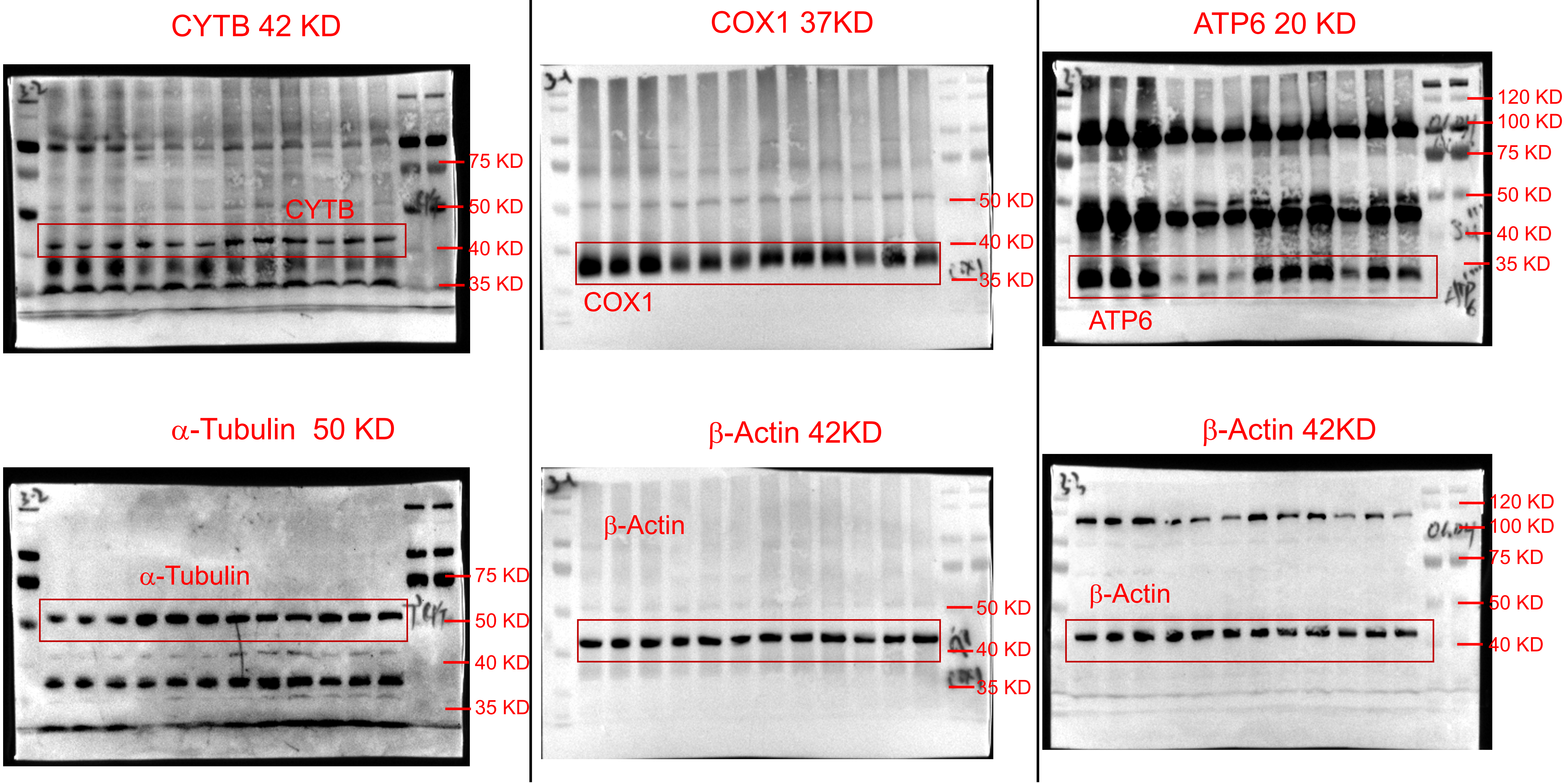

CYTB 42 KD
CYTB
75 KD
50 KD
40 KD
35 KD
COX1 37KD
COX1
50 KD
40 KD
35 KD
ATP6 20 KD
ATP6
120 KD
100 KD
75 KD
50 KD
40 KD
35 KD
α-Tubulin 50 KD
α-Tubulin
75 KD
50 KD
40 KD
35 KD
β-Actin 42KD
β-Actin
50 KD
40 KD
35 KD
β-Actin 42KD
β-Actin
120 KD
100 KD
75 KD
50 KD
40 KD

Figure S5D

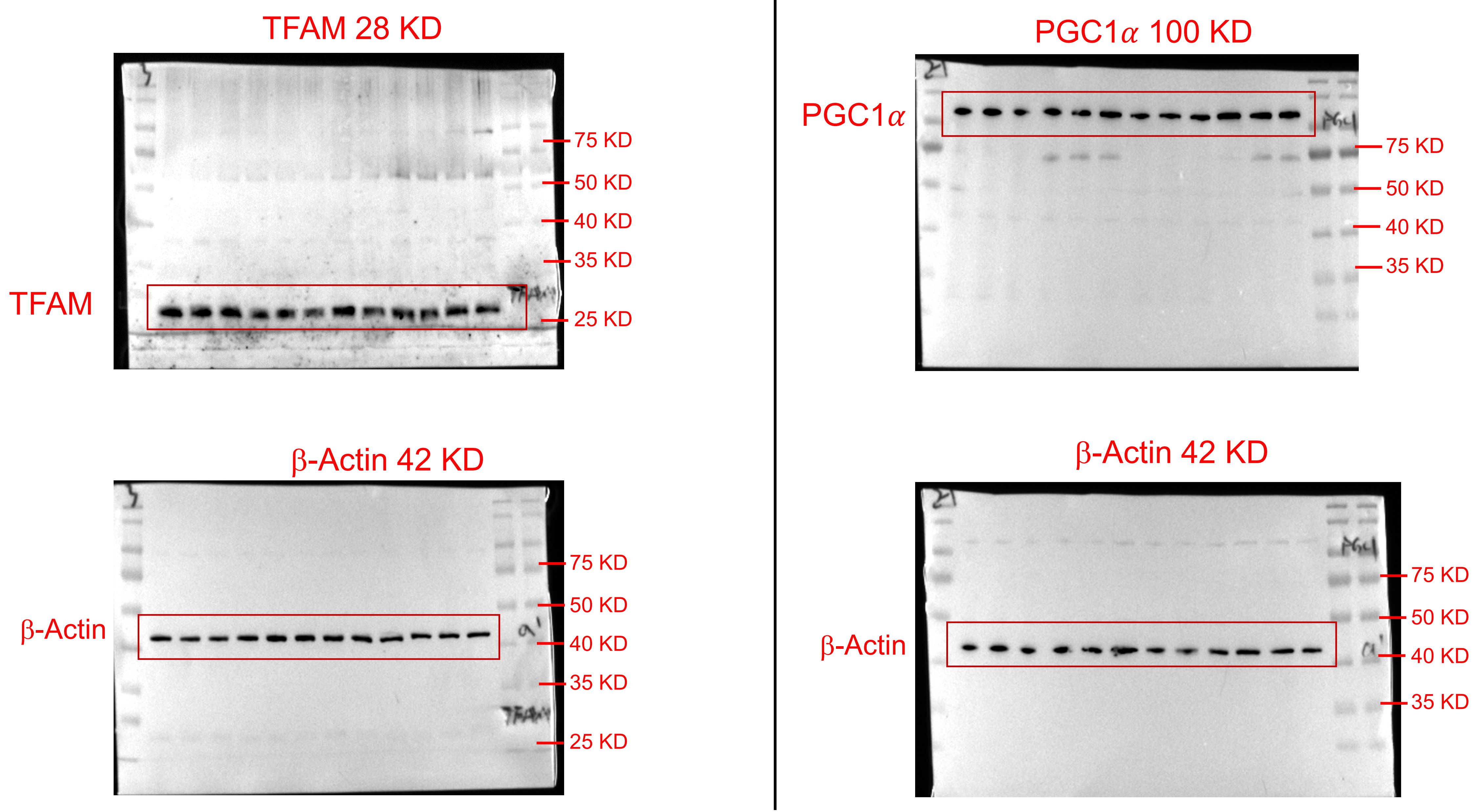
TFAM 28 KD
TFAM
75 KD
50 KD
40 KD
35 KD
25 KD
PGC1α 100 KD
PGC1α
75 KD
50 KD
40 KD
35 KD
β-Actin 42 KD
β-Actin
75 KD
50 KD
40 KD
35 KD
25 KD
β-Actin 42 KD
β-Actin
75 KD
50 KD
40 KD
35 KD

Figure S5E

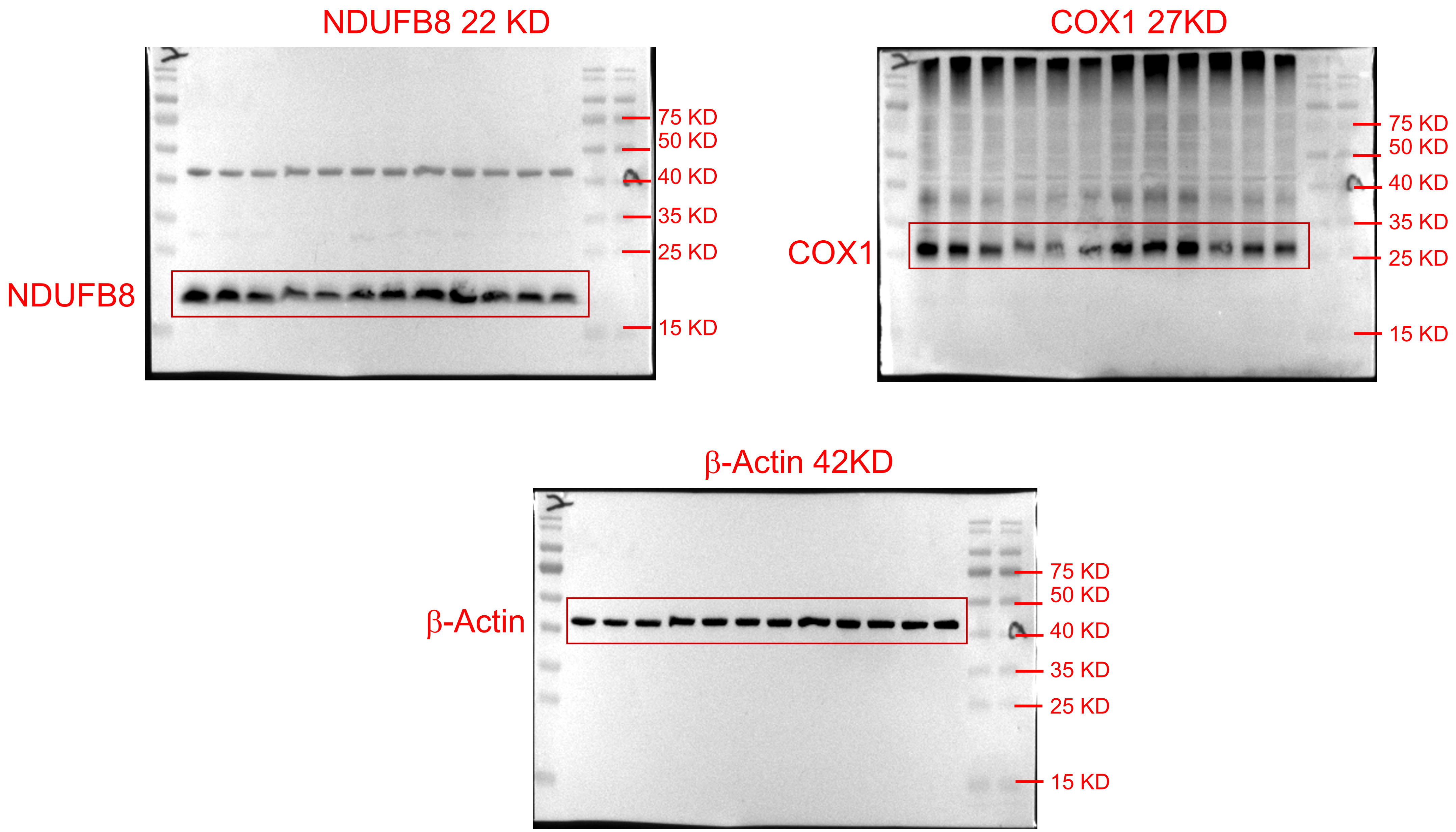
NDUFB8 22 KD
NDUFB8
75 KD
50 KD
40 KD
35 KD
25 KD
15 KD
COX1 27KD
COX1
75 KD
50 KD
40 KD
35 KD
25 KD
15 KD
β-Actin 42KD
β-Actin
75 KD
50 KD
40 KD
35 KD
25 KD
15 KD

Figure S5E

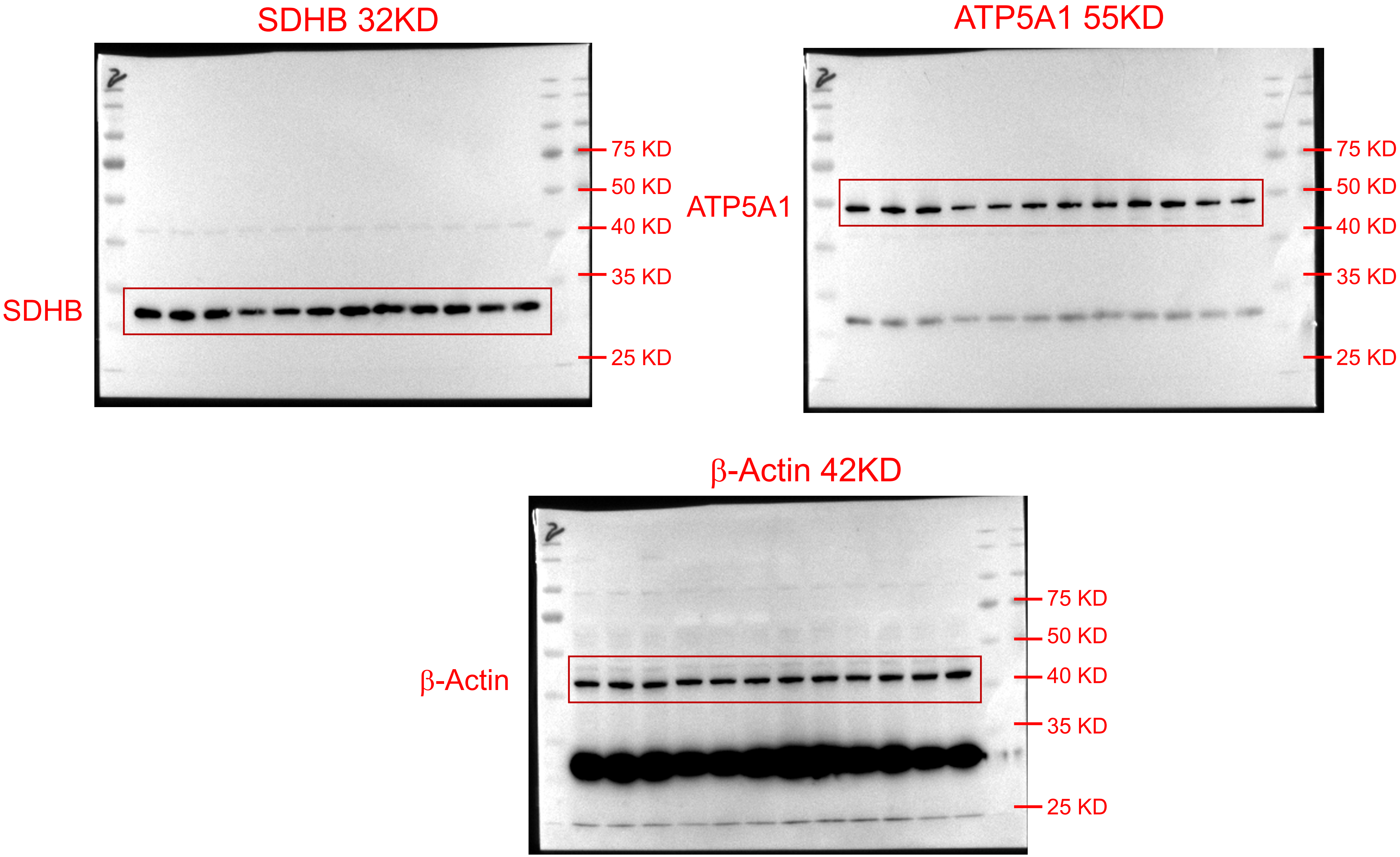
SDHB 32KD
ATP5A1 55KD
75 KD
50 KD
40 KD
35 KD
25 KD
SDHB
ATP5A1
75 KD
50 KD
40 KD
35 KD
25 KD
β-Actin 42KD
β-Actin
75 KD
50 KD
40 KD
35 KD
25 KD

Figure S5E

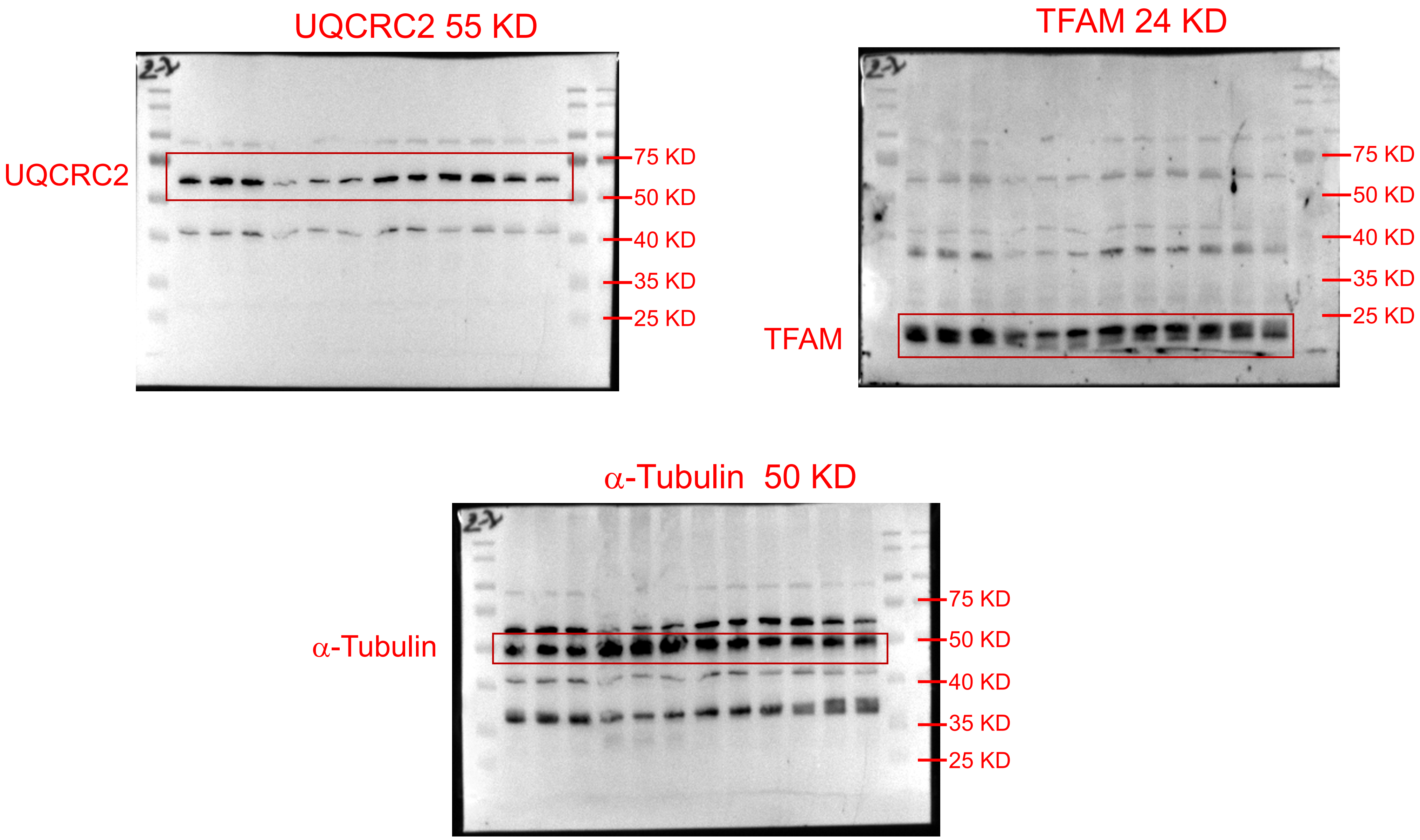
UQCRC2 55 KD
UQCRC2
75 KD
50 KD
40 KD
35 KD
25 KD
TFAM 24 KD
TFAM
75 KD
50 KD
40 KD
35 KD
25 KD
α-Tubulin 50 KD
α-Tubulin
75 KD
50 KD
40 KD
35 KD
25 KD

Figure S6B

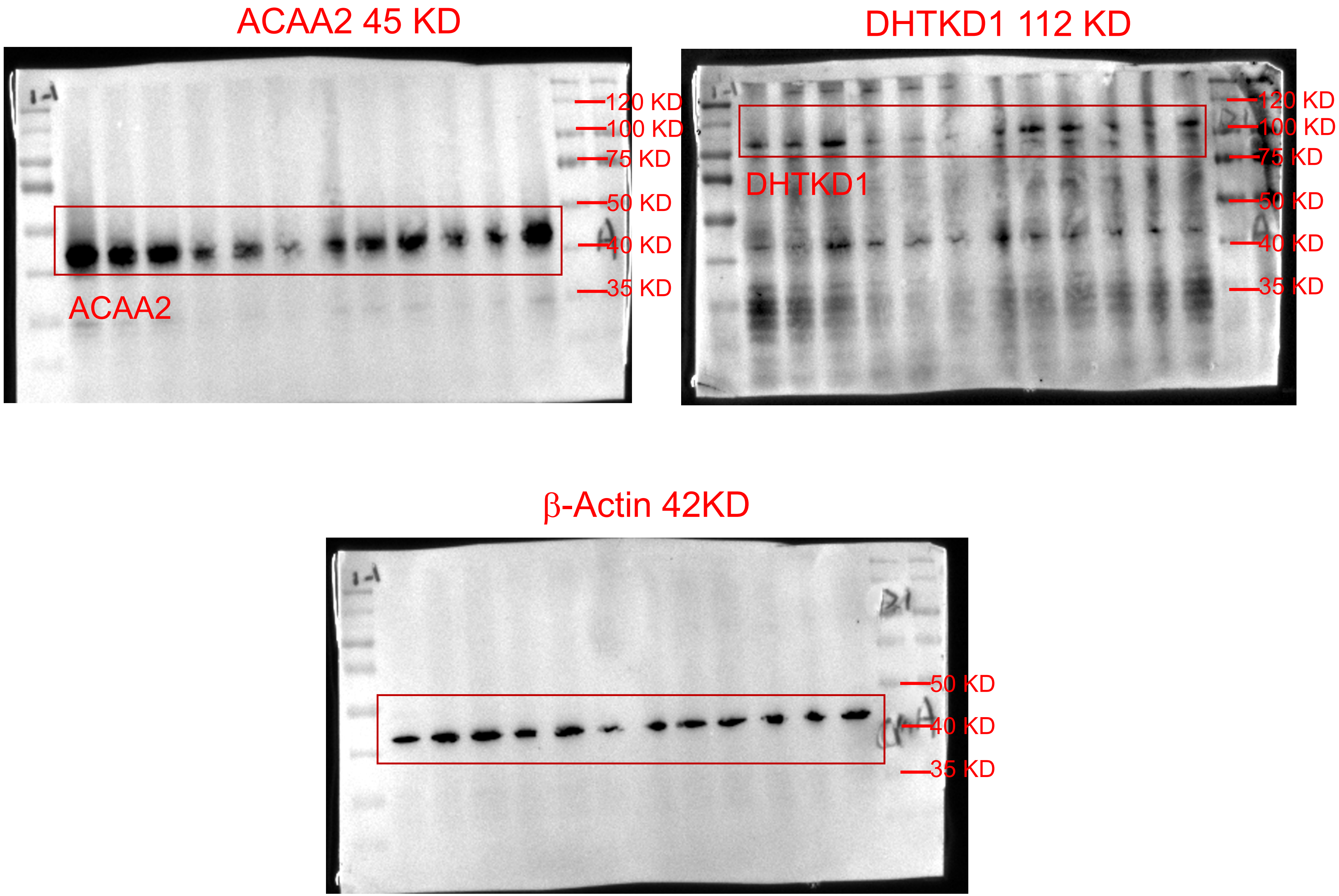
ACAA2 45 KD
120 KD
100 KD
75 KD
50 KD
40 KD
35 KD
ACAA2
DHTKD1 112 KD
120 KD
100 KD
75 KD
50 KD
40 KD
35 KD
DHTKD1
β-Actin 42KD
50 KD
40 KD
35 KD

Figure S6D

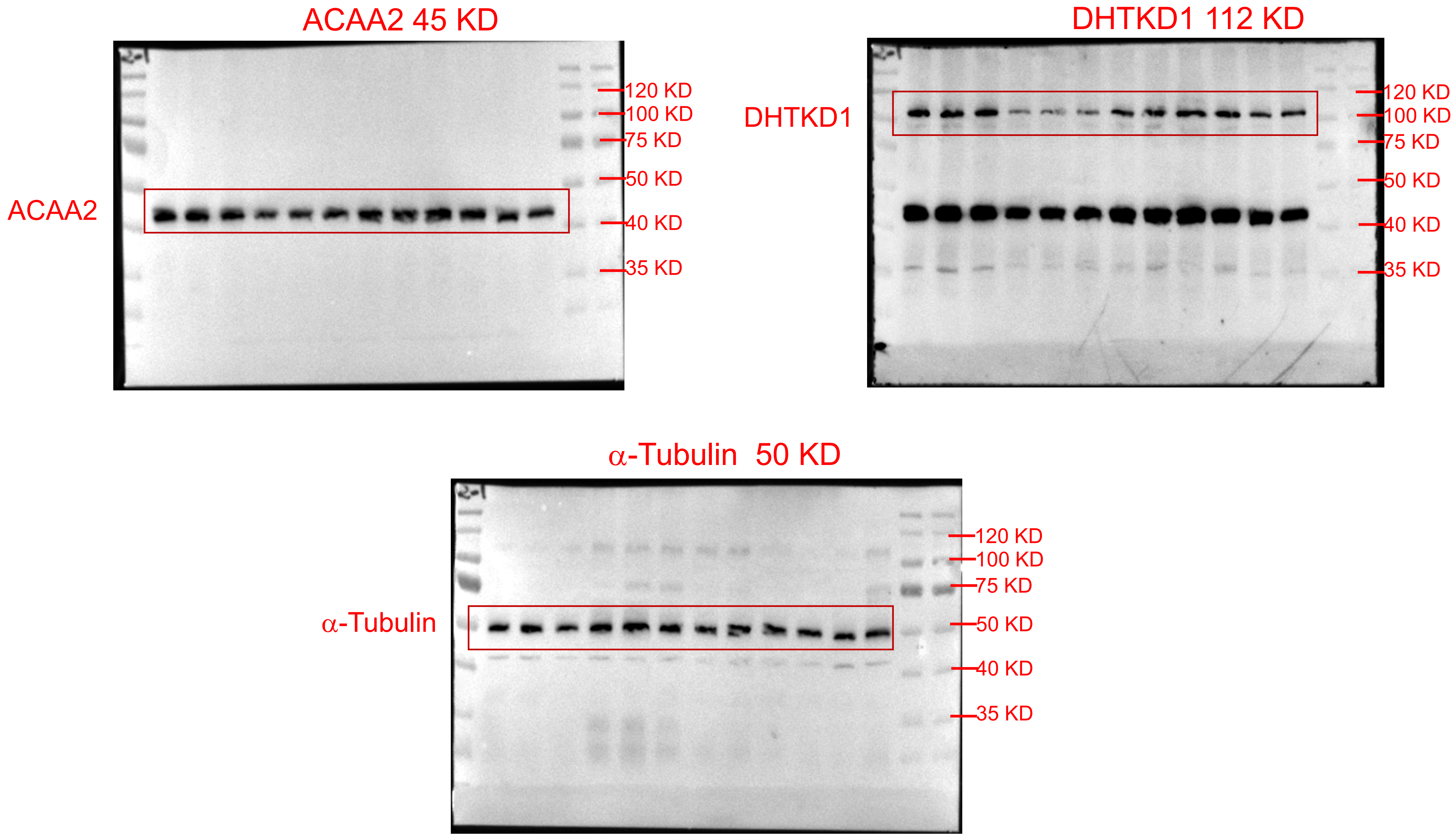
ACAA2 45 KD
ACAA2
120 KD
100 KD
75 KD
50 KD
40 KD
35 KD
DHTKD1 112 KD
DHTKD1
120 KD
100 KD
75 KD
50 KD
40 KD
35 KD
α-Tubulin 50 KD
α-Tubulin
120 KD
100 KD
75 KD
50 KD
40 KD
35 KD

Figure S7B

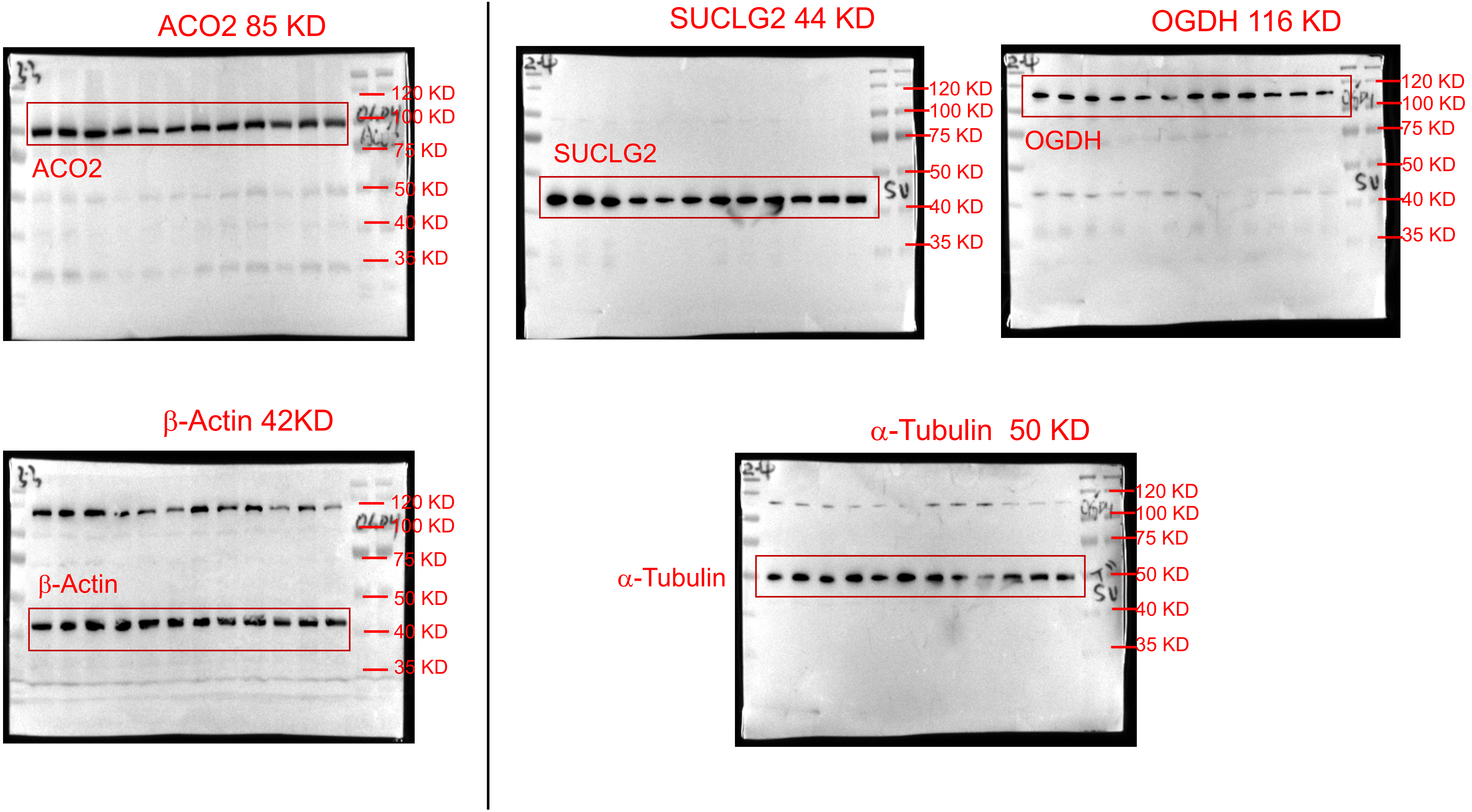

ACO2 85 KD
ACO2
SUCLG2 44 KD
SUCLG2
OGDH 116 KD
OGDH
β-Actin 42KD
β-Actin
α-Tubulin 50 KD
α-Tubulin
120 KD
100 KD
75 KD
50 KD
40 KD
35 KD

Figure S7B

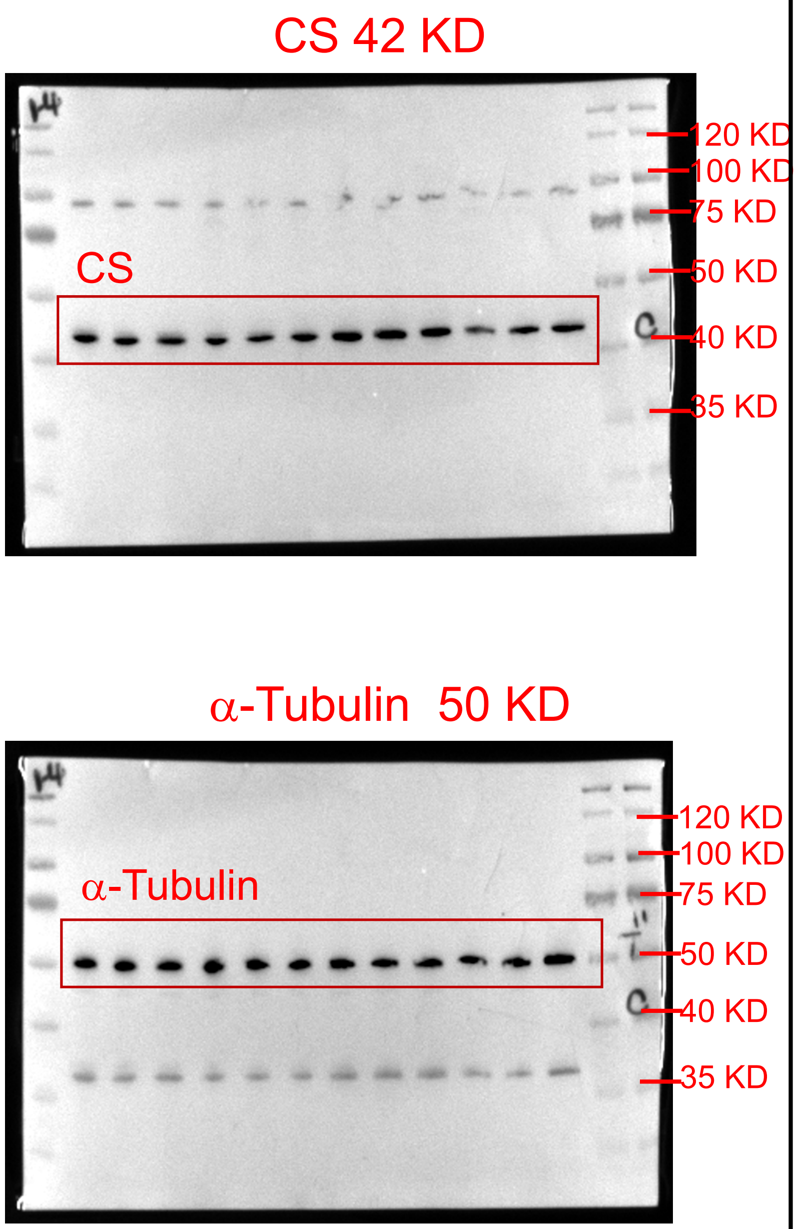

Figure S7D

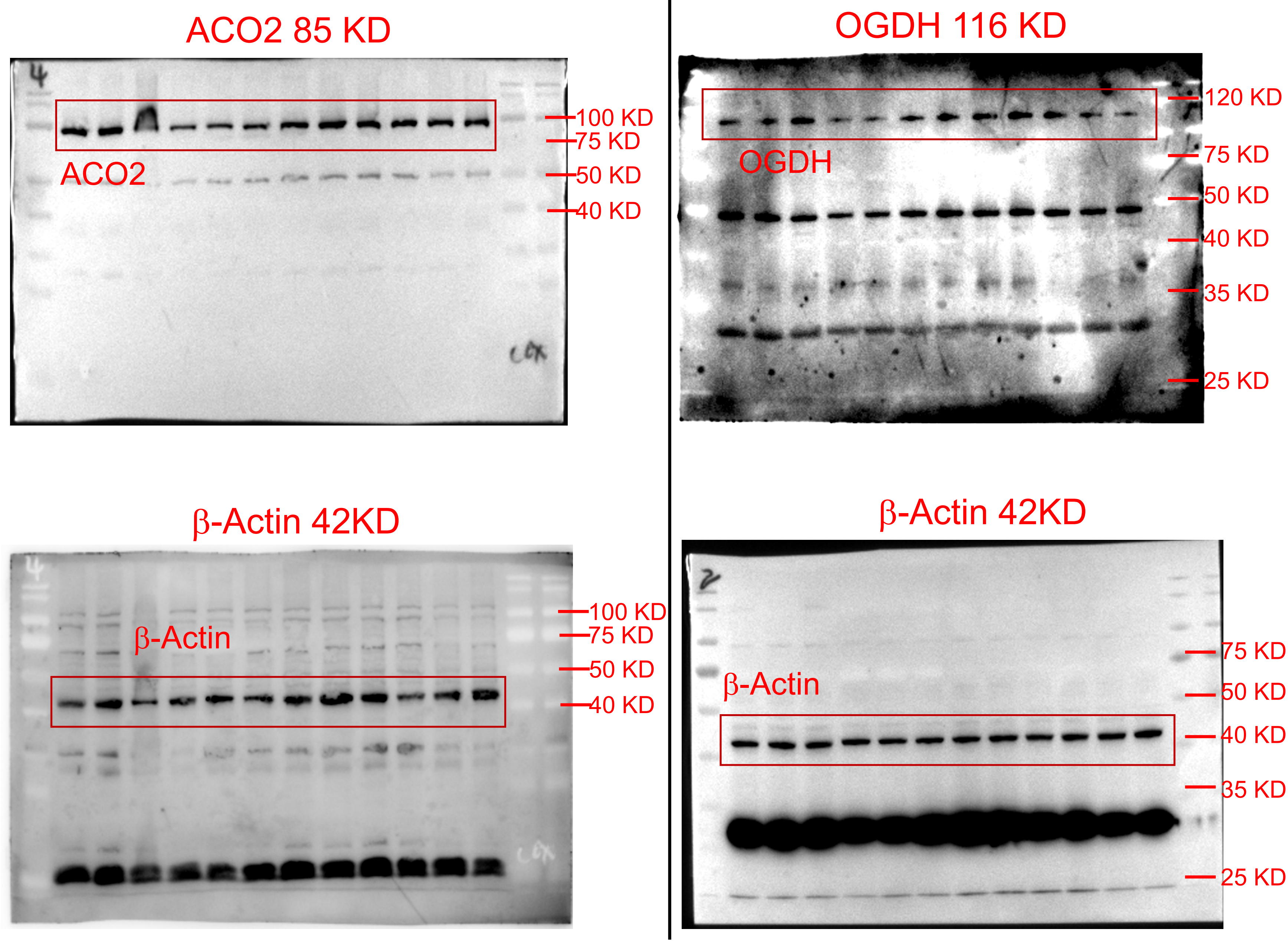

Supplement: Supplementary file 5 — Supplemental Material Original western blots [file 41419_2024_7231_MOESM5_ESM.pdf]
